# Employing complementary spectroscopies to study the conformations of an epimeric pair of side-chain stapled peptides in aqueous solution†

**DOI:** 10.1039/d0ra10167b

**Published:** 2021-01-20

**Authors:** Jonathan Bogaerts, Yoseph Atilaw, Stefan Peintner, Roy Aerts, Jan Kihlberg, Christian Johannessen, Máté Erdélyi

**Affiliations:** Department of Chemistry, University of Antwerp 2020 Antwerp Belgium; Department of Chemistry – BMC, Uppsala University SE-751 23 Uppsala Sweden mate.erdelyi@kemi.uu.se

## Abstract

Understanding the conformational preferences of free ligands in solution is often necessary to rationalize structure–activity relationships in drug discovery. Herein, we examine the conformational behavior of an epimeric pair of side-chain stapled peptides that inhibit the FAD dependent amine oxidase lysine specific demethylase 1 (LSD1). The peptides differ only at a single stereocenter, but display a major difference in binding affinity. Their Raman optical activity (ROA) spectra are most likely dominated by the C-terminus, obscuring the analysis of the epimeric macrocycle. By employing NMR spectroscopy, we show a difference in conformational behavior between the two compounds and that the LSD1 bound conformation of the most potent compound is present to a measurable extent in aqueous solution. In addition, we illustrate that Molecular Dynamics (MD) simulations produce ensembles that include the most important solution conformations, but that it remains problematic to identify relevant conformations with no *a priori* knowledge from the large conformational pool. Furthermore, this work highlights the importance of understanding the scope and limitations of the available techniques for conducting conformational analyses. It also emphasizes the importance of conformational selection of a flexible ligand in molecular recognition.

## Abstract 2

Spectroscopic evaluation of an epimeric pair of macrocycles identified their solution ensembles, and the conformation responsible for bioactivity.

## Graphical abstract

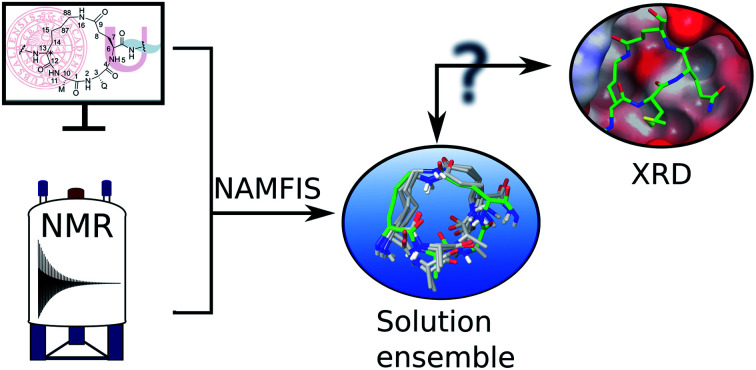

## Introduction

Understanding molecular recognition is of key importance for drug discovery. It has traditionally been explained by Fischer's ‘lock-and-key’ hypothesis,^1^ and subsequently by Koshland's ‘induced fit model’^2^ in text books. Whereas the former theory presumes the interaction of rigid bodies, the latter allows for conformational adjustment of the protein to promote the most favorable interactions with its binding partner. ‘Conformational selection’, the most recent alternative model, recognizes the simultaneous presence of several protein conformations in solution and suggests that binding alters the population of pre-existing ligand solution conformers, rather than changing the protein conformation.^3^ As a result, the ligand conformation that is bound by the protein is favored in the solution ensemble. This theory originates from the energy landscape hypothesis of protein dynamics, and accordingly recognizes the importance of protein dynamics for drug binding and considers the conformational flexibility of drug candidates to a lesser extent. As none of the past decades' models takes ligand flexibility into account, it is unsurprising that current docking algorithms typically fit rigid ligand geometries into a flexible protein binding site. Medicinal chemists have traditionally utilized ligand rigidification strategies, such as macrocyclization,^4,5^ to improve target affinity, on the premise that a ligand with a preorganized conformation ought to have higher affinity due to entropic and enthalpic reasons as compared to a molecule that can adopt multiple conformations in solution. Macrocyclization has been a successful strategy also for peptides,^6^ which in their linear form show an unusually large degree of conformational freedom. Peptides have therefore often been selected as model systems for the evaluation of the influence of conformation and flexibility on bioactivity,^7–11^ and of the dependence of ligand conformation on environment polarity.^12^ Conformational flexibility has lately been shown to be essential for both membrane permeability^13,14^ and ligand binding to larger protein surfaces.^15^ Moreover, understanding the conformational preferences of free ligands in solution has proven to be necessary for rationalization of structure–activity relationships (SAR) to enable effective structure-based drug design.^16,17^ Presuming that the bioactive conformation of a flexible ligand is present among its solution conformers, the understanding of the solution ensemble of a ligand may be a suitable, or sometimes the only route, for the elaboration of its bioactive conformation, for instance when a high-resolution X-ray structure of the protein-ligand complex is unavailable, and saturation difference- or HSQC-type experiments cannot be used.^18^

**Fig. 1:**
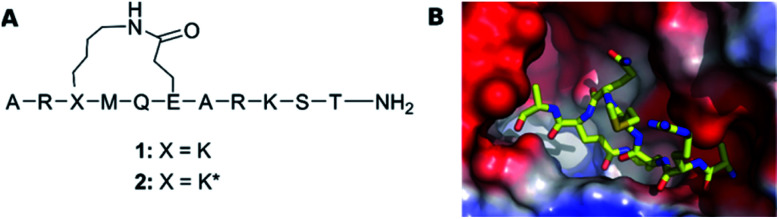
(A) The structure of LSD-1 inhibitory peptides with a macrocyclic central core structure, possessing a lactam bridge between Lys^3^ and Glu^6^. (B) The crystal structure of LSD-1-CoREST in complex with peptide 2 (PDB ID: 6S35) adapted from Yang *et al.*^19^ The surface of LSD-1 is colored reflecting its electrostatic potential (red-negative, blue-positive) whereas 2 is represented as a stick-model with N, C and O atoms colored in respectively blue, yellow and red.

We examine herein whether the bioactive conformation of a flexible ligand is identifiable in solution, for a system where the protein bound conformation was determined unambiguously by X-ray crystallography. For this purpose, similar to many previous studies we chose a peptide model system. Two macrocyclic inhibitors of the FAD dependent amine oxidase lysine-specific demethylase 1 (LSD-1) were selected (1 and 2, Fig. 1A), as they encompass a semi-flexible macrocycle and a fully flexible pentapeptide chain. Despite only differing at a single stereocenter, Lys^3^, these show a large difference in bioactivity (*K*_i_ of 1: 31 μM, and of 2: 2.3 μM), and the bioactive conformer of 2 is known (Fig. 1B).^19^ In complex with the repressor element-1 silencing transcription factor (REST) corepressor 1 (CoREST) LSD-1 plays an important role in transcription regulation, by removing methyl groups selectively from the fourth (Lys4) position of the N-terminal tail of the histone H3 protein.^20^ However, when CoREST is interchanged with the androgen receptor, LSD-1 becomes specific for the ninth (Lys9) position of H3.^21^ Dysfunction of LSD1 has been linked to the development of acute myeloid and lymphoblastic leukemia, as well as breast and prostate cancer. Therefore, LSD-1 inhibition is an emerging option for novel cancer treatments.^22–24^

We used Raman optical activity (ROA) and NMR spectroscopy to evaluate whether the bioactive conformation, determined previously by X-ray crystallography,^19^ is available among the solution conformations of 1 and 2. The former technique has been proposed but not yet demonstrated to be able to describe solution ensembles for the type of system studied here, whereas the latter is seen as the gold standard for solution conformational studies. As there is a growing interest in the computational prediction of solution conformations,^25^ not at least of macrocycles,^26^ we also evaluated the predictive ability of classical molecular dynamics (MD) simulations.

## Results and discussion

### Synthesis

**Scheme 1:**
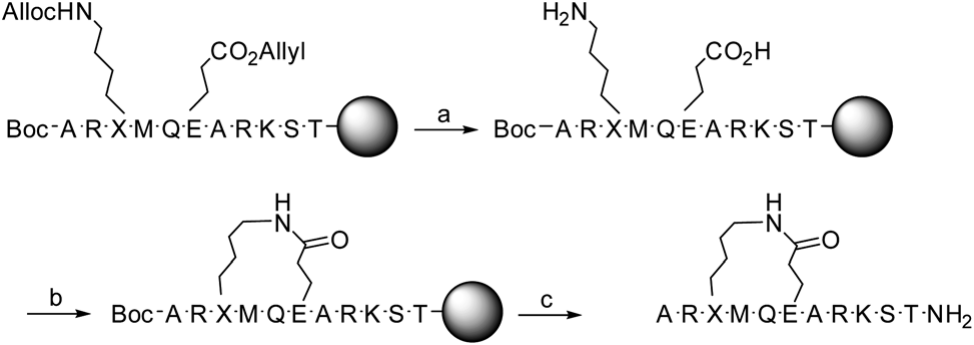
Synthetic pathway from linear precursor to lactam bridged peptides 1 (X = L–K) and 2 (X = D–K). Reagents and conditions: (a) NMP, Pd[P(Ph)_3_]_4_, AcOH, 3 h; (b) HCTU, DIPEA, DMF, 2 h; (c) TFA, Et_3_SiH, H_2_O, 1,2-ethandithiol and thioanisole (93 : 1 : 2.5 : 2.5 : 1), 1 h. Side-chain protecting groups are not shown for clarity; a detailed description of the synthesis is given in ref. 19.

Detailed description of the synthesis of 1 and 2 is given in ref. 19. In short, the linear precursors of peptides 1 and 2 were synthesized on solid-phase using the Fmoc-protection procedure. The Fmoc-protected lysine and glutamic acid were incorporated with allyloxycarbonyl (Alloc) and allyl side chain protection groups, respectively, allowing selective deprotection. The N-terminal alanine was incorporated with Boc-protection. Following selective deprotection of the allyloxycarbonyl and allyl groups with Pd[P(Ph_3_)]_4_, lactamization was achieved, using HCTU. Next, the remaining protective groups were removed with TFA simultaneous to cleavage from the solid phase (Scheme 1). Purification on reversed-phase HPLC gave 1 and 2 as trifluoroacetic acid salts in 9–10% yield, with their identity being confirmed by MALDI-TOF MS and NMR.

### Raman optical activity (ROA)

**Fig. 2:**
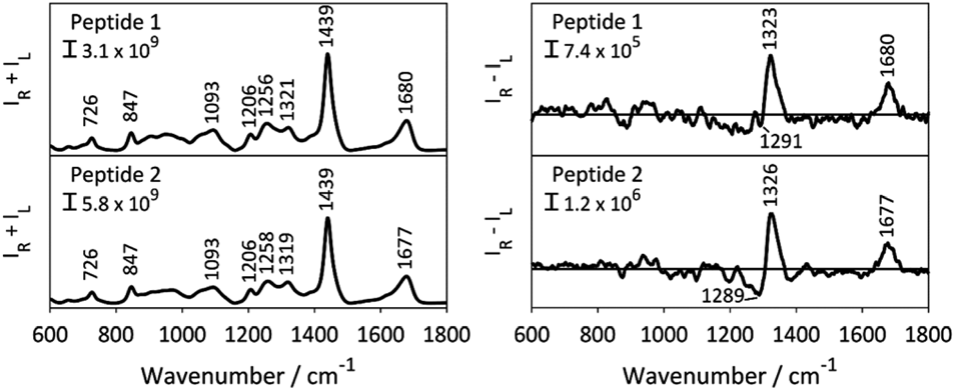
Raman (left, *I*_R_ + *I*_L_) and ROA (right, *I*_R_ − *I*_L_) spectra of peptide 1 and 2. The ROA spectra were baselined by overly smoothing of the ROA spectra using a Savitzky–Golay filter. *I*_R_ and *I*_L_ are respectively the intensities of right- and left circular polarized Raman scattered photons.

The Raman and ROA spectra of the aqueous solutions of 1 and 2 are presented in Fig. 2. During the experiment, the intensity of both right- and left circular polarized Raman scattered photons (*I*_R_ and *I*_L_) is determined, where the Raman and ROA signals are the sum (*I*_R_ + *I*_L_) of and difference (*I*_R_ − *I*_L_) between the two intensities, respectively. Both spectra show distinct spectral patterns typical for structurally disordered peptides.^27–29^ The broad band at ∼1680 cm^−1^ (amide I region) and the 1256 cm^−1^ Raman band, observed for both peptides, are diagnostic of a disordered structure or random protein/peptide conformation.^28^ The highly similar spectral pattern of the two samples is expected for epimeric peptides. The ROA spectral patterns of proteins and peptides mainly arise from their most rigid parts, their backbone, whereas the contribution of the side chains, which are prominent in the Raman spectra, mostly cancel out due to their flexible nature.^30^ Consequently, ROA spectral features are mainly observed in three distinct spectral regions: (i) the backbone skeletal stretch (870–1150 cm^−1^) which includes vibrations from C_α_–C, C_α_–C_β_ and C_α_–N bonds, (ii) the extended amide III region arising from the coupling of the in-plane N–H vibration with C–N stretching and C_α_–H bending (1230–1340 cm^−1^), and (iii) the amide I region originating from the carbonyl stretch of the polyamide backbone.^31^ The latter two regions were shown to be sensitive to the secondary structure of proteins and peptides.^29,32,33^

The ROA spectra of 1 and 2 depict a rather featureless pattern in the backbone skeletal stretch, and broad positive bands at 1323 cm^−1^ and 1677 cm^−1^, in the amide III and amide I regions, respectively. For proteins and peptides, this ROA pattern indicates a polyproline II (PPII) peptide backbone structure.^28,29,34^ A PPII helix is a common conformational element, characterized by the backbone *ϕ* and *ψ* torsion angles clustering around *ϕ* = −75° and *ψ* = 145° in the Ramachandran plot, and is observed also for sequences that do not contain proline.^35^ Negative contributions in the 1200–1300 cm^−1^ region of the ROA spectrum were previously reported for intrinsically disordered proteins with large portions of PPII structure, such α-synuclein.^28^ However, recent computational work by Mensch *et al.* suggested that this negative contribution to the ROA spectrum might arise from a helical structure.^36^ As such, the difference in intensity in the band at 1289 cm^−1^ hints a difference in the backbone conformation of 1 and 2. However, it is difficult to derive a conclusion from this observation without risking data over-interpretation. As such, the ROA spectra of the two epimeric side-chain stapled peptides exhibit highly similar patterns. This is surprising, as ROA previously has been shown to be able to distinguish between epimers.^37,38^ The reason why the ROA spectra observed in this case are so similar, is likely to be due to the observed ROA spectra being dominated by the signals arising from the linear C-terminus (Res 7–11), which is the same for both peptides (Fig. 1A), obscuring any signals originating from the epimeric macrocycle. This spectral behaviour has previously been observed for β-turns in peptides, whose signals are no longer visible due to stronger bands coming from the other secondary structure.^39^ In addition, the assignment of disordered structure of the C-terminus is in line with the X-ray structure of peptide 2 lacking electron density for these resisdues.^19^ Importantly, this indicates that the structural basis of the different binding affinity of the peptides cannot be rationalized by means of ROA spectroscopy and that atomic resolution, provided by NMR spectroscopy, might be necessary to understand their different conformational behaviour.

### NMR

For the description of the solution ensembles of peptides 1 and 2, NOE build-ups were acquired for their H_2_O/D_2_O (9 : 1) solutions using *t*_mix_ = 100–700 ms on a 600 MHz spectrometer, equipped with a TCI cryogenic probe. Inter-proton distances were determined using geminal methylene protons (1.78 Å) as internal reference. Together with dihedral angles derived from vicinal scalar couplings (^3^*J*_NH–CαH_) the inter-proton distances were deconvoluted into solution ensemble using NAMFIS (NMR Analysis of Molecular Flexibility in Solution),^40^ an algorithm that has previously been successfully applied for the determination of solution ensembles of peptidic^15,40–45^ and non-peptidic^13,18,26,46–49^ macrocycles, and also for distinguishing diastereoisomers.^42,43^

**Fig. 3:**
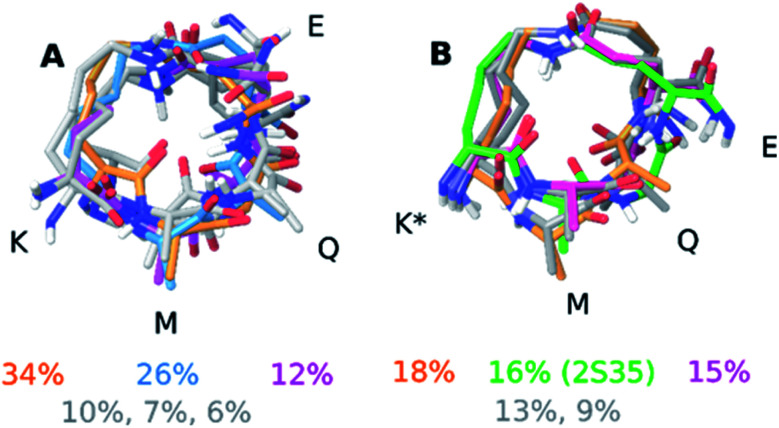
Solution ensemble of peptide 1 (A) and peptide 2 (B) as determined by the NAMFIS analysis. Overlays were generated by alignment of the heavy atoms in the macrocycle. The percentages refer to the weights that were determined by the NAMFIS analysis and correspond to the structure in the same color. The crystal structure of peptide 2 is shown in green in panel (B). The N- and C-terminal together with the nonpolar hydrogens have been omitted for clarity. The four amino acids that participate in the formation of the macrocycle are highlighted with their one letter code in black.

The computational input, *i.e.* a full ensemble covering the entire conformational space of the compounds, was generated by an unrestrained Monte Carlo conformational search using the Batchmin algorithm as incorporated into the Schrödinger software. Importantly, the NAMFIS algorithm does not rely on computed energies, which are force field dependent, but is entirely driven by experimental (NOE, *J*) data.^50,51^ The solution ensembles identified by this analysis are shown in Fig. 3. As amino acids 1-2 and 7-11 are identical and disordered for both 1 and 2, also in the solid state,^19^ our analysis focused on the conformation of the macrocycle whereas neglecting the disordered termini.

**Fig. 4:**
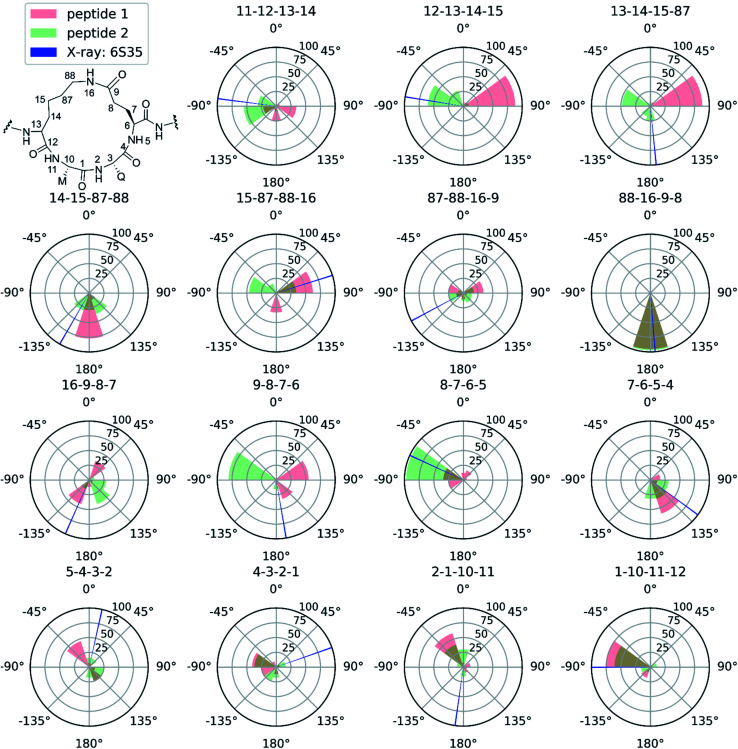
Selected torsional angles describing the macrocycle conformation of peptides 1 and 2. The amide dihedral angles of the backbone are not shown as those are all in the trans conformation. The dihedral angles are displayed as pseudo-Newman projections, with the dihedral angle specified above each polar histogram. Every wedge covers 36° and the height represents the NAMFIS conformer distribution of the specific angles. Conformers with a weight below 1% are not taken into account. Red: dihedral angles accessible for peptide 1 according to NAMFIS analysis. Green, dihedral angles accessible for peptide 2 as determined by NAMFIS analysis. Blue dihedral angle of peptide 2 bound to LSD-1 in the crystal structure (PDB ID: 6S35) with a wedge width of 1°. Overlapping red and green regions appear in brownish color.

Next to pinpointing the conformers present in solution and quantifying their population, the NAMFIS analysis identified the bioactive, LSD-1-bound conformer (Fig. 1B), to be present in the solution of 2 (16%) but not in that of 1 (ESI†). This is in line with the ten-fold lower binding affinity of 1 as compared 2 to LSD-1, and is the consequence of their different configuration at their C-13 (Fig. 4). The fact that the bound conformation of 2 is found in solution suggests that conformation selection takes place also upon protein binding of a flexible ligand, analogous to the conformation selection of the protein binding site. Whereas this may be unsurprising, the conformation selection has so far scarcely been discussed from the ligand point of view. Dihedral angles of the macrocyclic core of 1 and 2 are shown in Fig. 4, with the blue line in each subfigure highlighting the angle corresponding to the bioactive, LSD-1 bound, peptide conformation. Overall, the macrocycle is flexible, corroborating the ROA-based conclusions. Most torsional angles of the macrocyclic core of 1 and 2 are similar, indicating comparable overall orientation of the backbone of the two peptides. The most notable difference is seen for the torsional angle 12-13-14-15, *χ*_1_ of the epimeric l/d-*K*^3^, which shows mirrored preference (g^+^*vs.* g^−^) for the two peptides. Further significant differences are seen in the preference of the dihedral angles 11-12-13-14 and 13-14-15-87, which also involve *K*^3^, the epimerized amino acid. The different orientation preference of this region of the macrocycle in solution is a likely cause of the different bioactivities of peptides 1 and 2. Major differences are also observed for the orientation of the torsional angles 15-87-88-16, 16-9-8-7 and 9-8-7-6, which describe + the side-chain bridge orientation, induced by the configurational difference at C-13 (Fig. 4).

### MD simulation

We evaluated whether classical MD (MD) simulations are capable of predicting the conformational preferences of peptides 1 and 2 with no *a priori* knowledge of the conformational behaviour. Throughout the simulation snapshots were saved from the respective (combined) trajectories at 10 ps intervals, for both peptides, and the dihedral angles of the macrocycle were extracted (Fig. S2, ESI†). The predicted flexibility is in good agreement with the experimental observations. The MD trajectories indicate that some torsional angles sampled by 2 are not accessed by 1 (see Fig. S2, ESI†). Unexpectedly, these differences are found for torsional angles that do not belong to *K*^3^, the point of epimerization, but rather to Q5 and E^6^ (9-8-7-6, 8-7-6-5, 4-3-2-1 and 2-1-10-11).

**Fig. 5:**
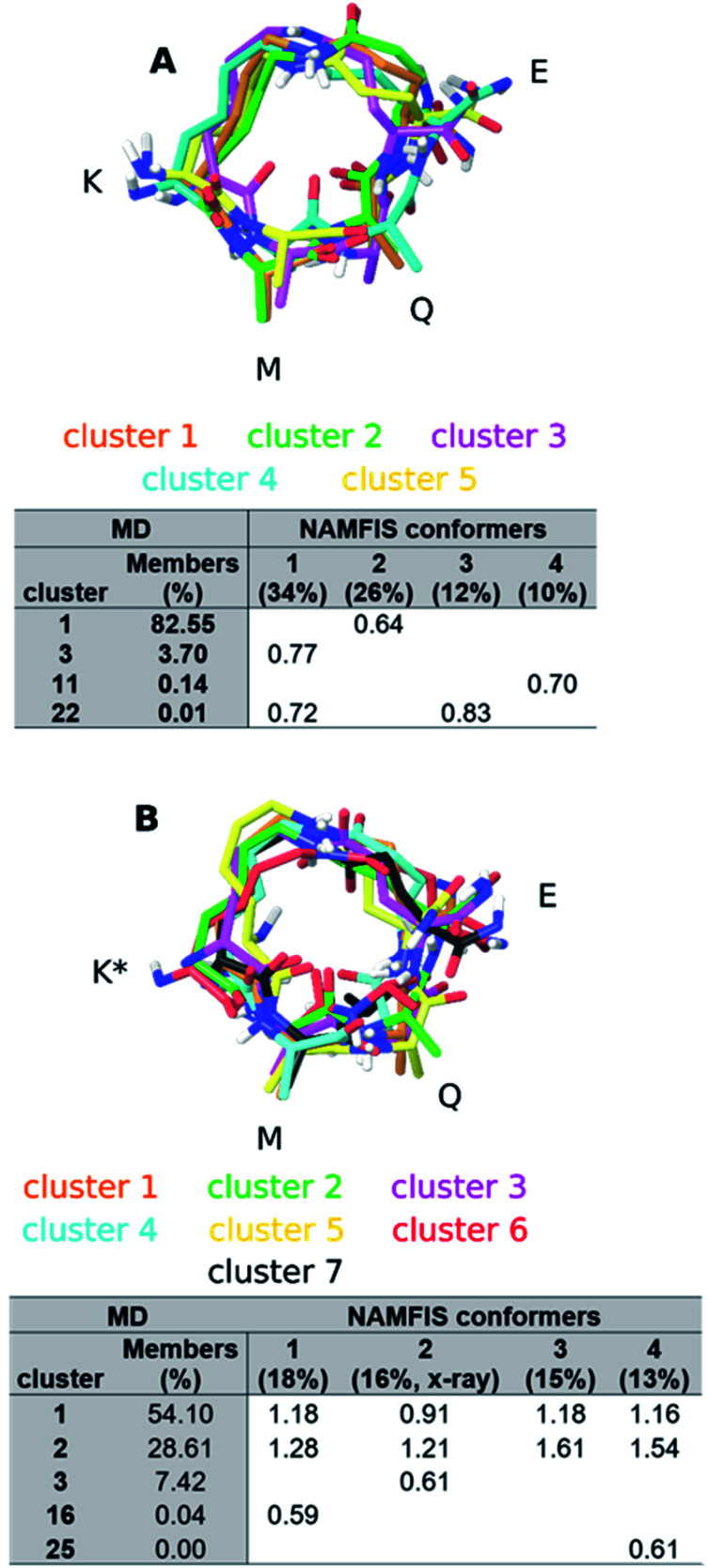
Central structures of the clusters containing 300 or more structures of peptide 1 (A) and peptide 2 (B). Overlays were created by aligning the heavy atoms of the macrocycle. The N- and C-terminal together with the nonpolar hydrogens have been omitted for clarity. The amino acids composing the macrocycle are highlighted with their one letter code. The color of the cluster name corresponds to the structure in the same color. The tables underneath each structure summarizes the RMSD analysis between the MD clusters and the NAMFIS ensemble.

In order to simplify the analysis of the conformational space accessed by both peptides during the MD trajectories, we clustered the structures of the MD trajectories using the GROMOS algorithm described by Daura *et al.*,^52,53^ employing a mass-weighted root-mean-square deviation (RMSD) cutoff of 0.7 Å on the atoms of the macrocycle as similarity criterion. This resulted in 26 clusters for both peptides. The central structure of the clusters containing 288 and 300 member structures (∼≥1.0% out of the complete MD trajectory), respectively, are shown in Fig. 5. Subsequently, the RMSD between the central structure of each cluster, which represent the accessed conformational space of the macrocycle during the MD simulations, and the conformers with a weight above 10% in the NAMFIS ensemble were calculated (Table S10 and S11†). Two structures with pairwise RMSD ≤ 0.7 Å were categorized as comparable.

For peptide 1, the center of cluster 1 (>82%, Fig. 5A) is similar to the experimentally determined conformer 2 (26%, Fig. 5A), while conformer 4 (10%) is similar to the center of cluster 11 (<1%). The most populated experimental conformer 1 (34%) is found to be quite similar to the center of cluster 3 (<4%, RMSD = 0.77 Å) or cluster 22 (<1%, RMSD = 0.72 Å), when one looks slightly beyond the cut-off of 0.7 Å. The experimental conformer 3 has the lowest mutual RMSD of 0.83 Å to cluster 22 and is therefore considered to not be present within the cluster centers representing the MD trajectory. This does not mean that there is not a single structure within the full MD trajectory that is similar to conformer 1 or 3 of 1 (RMSD < 0.7 Å), but without having the experimental data available, these two conformations would not have been considered to be present in solution. All except one experimental conformations of 2 was found to be similar to at least one of the MD cluster centers (Table S11, ESI†). The bioactive conformation 2 (16%) is found in cluster 3, representing 7% of the MD ensemble (Fig. 5B). Conformers 1 (18%) and 4 (13%) are similar only to clusters that represent less than 1% of the MD trajectory. It should be noted that none of the experimentally observed conformations coincide with clusters 1 or 2, which populate 54% and 29% of the MD trajectory, respectively.

Overall, MD along with the GROMOS clustering algorithm was able to sample almost all relevant solution conformers without *a priori* knowledge, albeit with population weights (based on the size of the clusters) largely different from experimental ones. Hence, extracting relevant conformers from an MD trajectory without prior knowledge of the experimental ensemble is cumbersome as these are buried in a large amount of non-relevant structures common to long MD runs. This would certainly affect the outcome of a virtual screening, for example. We would also like to emphasize that the outcome of the MD analysis vastly depends on the choice of RMSD cutoff. Upon choosing the cutoff of 1 Å, instead of 0.7 Å from above, peptides 1 and 2 exhibit only seven clusters, with cluster 1 incorporating >93% of the conformers (Tables S12 and S13, ESI†), which contradicts the experimentally observed flexibility, for example. This raises the question what criterion to choose when classifying two conformations comparable, and indicates the difficulty of computational prediction of the solution ensemble of flexible compounds without experimental guidance. Furthermore, this observation highlights the need of developments of reliable conformation prediction algorithms for macrocycles, which constitute a novel burgeoning compound class to tackle difficult-to-drug protein targets.^4^

## Conclusions

Conformational flexibility has been shown vital for the bioactivity of macrocyclic drugs.^12,54,55^ Investigation of an epimeric pair of flexible LSD-1 inhibitors indicates that the bioactive conformation is present to a measurable extent in solution. Detection of the presence or absence of a distinct geometry might help drug development when a high-resolution X-ray structure of the target bound structure is unavailable. Our work highlights the importance of conformational selection of a flexible ligand in molecular recognition.

We also illustrate that an improved understanding of the scope and limitations of the available techniques for conformational analysis is of great importance. Our work provides evidence that Raman and ROA analysis in itself might not be capable of detecting minor differences in the conformational ensemble of highly similar macrocycles encompassed in (flexible) peptides, and that NMR spectroscopy is better suited for this challenge. From the ROA perspective, a comparison with NMR spectroscopy is valuable in order to estimate where and how ROA can or cannot provide added value.

Whereas MD simulations may be able to capture the most important conformations present in solution, it remains delicate to identify the relevant conformers from a large conformational pool with no *a priori* knowledge. In this respect, our findings agree well with those of a previous study comparing four different methods for conformational sampling of non-peptidic macrocyclic drugs.^26^ Importantly, the presented epimeric compound pair, with thoroughly characterized solution ensembles, offers an excellent model system for developers of computational techniques.

The accurate experimental description of macrocycles' solution conformations remains a challenge. For an accurate description, one cannot assume the existence of a single solution conformer such as the target bound conformer determined by X-ray crystallography. The application of experimental constraint driven molecular dynamics simulations are thus unsuitable. Instead the time-averaged spectroscopic data has to be deconvoluted into the molar fraction weighted spectroscopic data of an ensemble of conformations. This, in practice, means the simultaneous identification of the real-life solution conformers along with their molar fractions. Several approaches exist for such deconvolution, of which we chose to apply the NAMFIS algorithm that uses both computational and NMR spectroscopic inputs, and that has been described in detail elsewhere.^40^ Herein, we illustrate that the reliable determination of solution conformers is difficult, and necessitates the careful, simultaneous use of spectroscopic and computational techniques.

## Experimental

### Raman/ROA measurements

The samples, obtained by HPLC purification, were dissolved to a 50 mg mL^−1^ concentration in demineralised-water, loaded into a micro-fluorescence quartz cell (Starna Scientific Ltd.) and placed in the ChiralRAMAN-2X (Biotools Inc.) running at a resolution of 7 cm^−1^.^56,57^ The laser was set to 800 mW at the source with a 2.57 s integration time, using a total illumination time between 38 and 54 h. The Raman and ROA spectra were measured simultaneously and the intensities displayed as the sum (*I*_R_ + *I*_L_) and difference (*I*_R_ − *I*_L_) in circular intensities of the scattered light, respectively. Solvent spectra were subtracted from the Raman spectra after which the baseline procedure by Boelens *et al.*^58^ was applied. Cosmic ray spikes were removed from the ROA spectra by means of a median filter and the final ROA spectra were smoothed using a third-order, nine-point Savitzky–Golay filter.

### NMR spectroscopy

NMR spectra were recorded at 25 °C on a 600 MHz Bruker Avance Neo NMR spectrometer equipped with a TCI cryogenic probe using sample concentrations of 10 mg mL^−1^ in 9 : 1 H_2_O/D_2_O. Assignments were deduced from 1D (^1^H and ^13^C) and 2D (COSY, TOCSY, HSQC and NOESY) NMR spectra. NOESY buildups were recorded with mixing times of 100, 200, 300, 400, 500, 600 and 700 ms, with 16 scans, 2048 and 512 points in the direct (F2) and indirect (F1) dimension, respectively. The d_1_ relaxation delay was set to 2.5 s and water suppression was applied using excitation sculpting. Interproton distances were calculated from deduced NOESY buildup rates (*r*^2^ > 0.95) set relative to the buildup rate of a pair of geminal methylene protons (1.78 Å) used as internal distance reference. NOE peak intensities were calculated using normalization of both cross and diagonal peaks for each mixing time recorded: *η*_norm_ = ([cross peak_1_ × cross peak_2_]/[diagonal peak_1_ × diagonal peak_2_])^1/2^. The corresponding buildup rates (*σ*_ij_) were then converted into interproton distances (*r*_ij_) by applying the following equation: *r*_ij_ = *r*_ref_ × (*σ*_ref_/*σ*_ij_)^1/6^.

### NAMFIS analysis

The conformational space of the two peptides were identified by an unrestrained Monte Carlo conformational search using five different force fields (OPLS-2001,^59^ OPLS-2005,^60^ OPLS3e,^61^ AMBER*,^62^ and MMFF^63^), each with the GB/SA solvation models for chloroform and water. The conformational search was carried out using intermediate torsion sampling and 50 000 MC steps, and an RMSD cut-off set to 2.0 Å. Each conformation was minimized on a molecular mechanic level of theory, using the Polak–Ribière-type conjugate gradient (PRCG) with a maximum of 5000 iterative steps. All conformations within 42 kJ mol^−1^ from the global minimum were retained. The final ensemble used for the NAMFIS analysis was created by combination of the conformers from the 8 conformational searches followed by elimination of redundant conformations by comparison of heavy atom coordinates applying an RMSD cut-off set to 2.0 Å. The extensive conformational search was performed to ensure complete coverage of the available conformational space.

The solution ensembles of the two peptides were identified by fitting the experimentally measured dihedral angles (^3^*J*) and interproton distances (*r*_ij_ from NOEs) to those back-calculated for computationally predicted conformations, fitting the populations of the conformers with the NAMFIS algorithm, following literature protocols. Validation of the NAMFIS-derived ensemble analyses was performed by the addition of 10% random noise, random removal of individual restraints and by comparison of the experimentally observed and back-calculated distances.

### Molecular dynamics simulations

The initial structure of peptide 1 was generated by building the linear peptide using the xPEPZ module of MCPRO 3.2.^64^ Subsequently, the lactam bridge was manually created using Gaussview6 (ref. 65) after which the structure was pre-optimized using the “clean” function incorporated in the software. The initial structure of peptide 2 was generated by converting the chiral center of Lys^3^ of the previously built peptide 1. In this way, any prior knowledge of the macrocycle conformation is avoided. The MD simulations were performed with GROMACS 2020.2 (ref. 66 and 67) using the OPLS-2001 force field.^59^ The force field parameters for the unusual macrocycle, formed through the connection between de side chains of the Lys^3^ and Glu^6^ residue, were determined based on the force field parameters of their standard residue counterparts. The nitrogen of Lys^3^ was covalently attached to the carbon of Glu^6^, similar to the procedure used for simulations of disulfide bridged systems. The peptides were solvated, using TIP3P waters as implemented in GROMACS 2020.2, in a cubic box with periodic boundary conditions that extended 10 Å beyond any peptide atom. Solvent molecules were replaced with counter ions (Cl^−1^) using the ‘genion’ subroutine implemented in the Gromacs, to neutralize the net charge within the complete simulation box. Next, for both peptides the same procedure was followed. First, a steepest descent energy minimization, using the convergence criteria of 500 000 steps or a maximum force <10 kJ mol^−1^ nm^−1^, was conducted. Secondly, two 100 ps equilibration MD runs were carried out. One in the constant particle number, volume, temperature ensemble (NVT ensemble, using the modified Berendsen thermostat with velocity rescaling at 300 K and 0.1 ps time step and separate heat baths for peptide and solvent). The second in the constant particle number, pressure, temperature ensemble (NPT ensemble, with the use of Parinello–Rahman pressure coupling at 1 bar, a compressibility of 4.5 × 10^−5^ bar^−1^ and a time constant of 2 ps). A position restraint potential (force constant of 1000 kJ mol^−1^ nm^−2^) was added to all peptide atoms during both equilibration runs. Subsequently, coordinates and velocities at every 10 ps were saved during the production run (100 ns) using the same temperature and pressure schemes as applied in the equilibration runs. This simulation procedure was repeated another 2 times. For all MD simulations the leap-frog integrator was used with a time step of 2 fs and coordinates were saved every 2 ps. All bonds to hydrogen atoms were constrained using the linear constrained solver (LINCS) with an order of four and one iteration. A grid-based neighbor list with a threshold of 10 Å was used and updated every five steps (10 fs). The particle-mesh Ewald (PME) method was used for long-range electrostatic interactions above 10 Å with a fourth order interpolation and a maximum spacing for the FFT grid of 1.6 Å. Lennard–Jones interactions were cutoff above 10 Å. A long range dispersion correction for energy and pressure was used to compensate for the Lennard–Jones interaction cutoff.

## Conflicts of interest

There are no conflicts to declare.

## Supplementary Material

RA-011-D0RA10167B-s001
