# Supplementary material for: Employing complementary spectroscopies to study the conformations of an epimeric pair of side-chain stapled peptides in aqueous solution
Source: RSC Adv. 2021 Jan 20;11(7):4200–8. doi: 10.1039/d0ra10167b (PMC8694311; doi:10.1039/d0ra10167b)
Supplement: RA-011-D0RA10167B-s001 [file RA-011-D0RA10167B-s001.pdf]

Electronic Supplementary Material (ESI) for RSC Advances.
This journal is © The Royal Society of Chemistry 2021

Electronic Supporting Information (ESI)

Employing complementary spectroscopies to study the conformations of two highly similar peptides in aqueous solution

Jonathan Bogaerts, Yoseph Atilaw, Stefan Peintner, Roy Aerts, Jan Kihlberg, Christian Johannessen, Máté Erdélyi

Table of Contents

1. NMR analysis of peptides **1** and **2** S2
1.1. ^{1}H-NMR chemical shift assignments S2
1.2. NOE Build-up derived distances and *J* couplings S4
2. Monte Carlo molecular mechanics (MCMM) conformational search S6
3. X-ray crystal structure used for NAMFIS analysis S7
4. NAMFIS analysis S7
5. MD analysis of peptides **1** and **2** S10
6. NMR Spectra S14
References S19

1. NMR analysis of peptides **1** and **2**

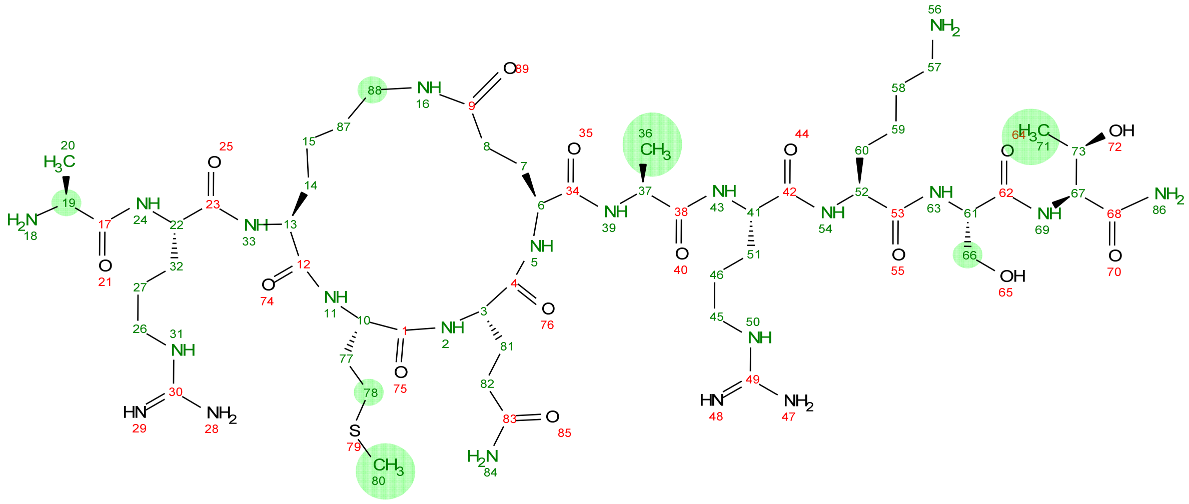

Figure S1: Structure of peptide **1**. The structure of peptide **2** is identical to that of **1** with the only difference in the epimerization at C-13 (L (**1**) to D (**2**)). The atom numbering shown here is used in the Tables below.

1.1. ^{1}H-NMR chemical shift assignments

NMR spectra of peptide **1** and peptide **2** were recorded at 25°C on a 600 MHz Bruker Neo NMR spectrometer equipped with a TCI cryogenic probe in D_2O:H_2O (1:9). Assignments were deduced based on 1D (^{1}H and ^{13}C) and 2D (COSY, TOCSY, HSQC and NOESY) NMR spectra. The chemical shifts assignments of protons that are used in the NAMFIS analysis are summarized in Table S1. Original FIDs are available, free of charge open access, at Zenodo with DOI: 10.5281/zenodo.4299784.

Table S1. ^{1}H-NMR chemical shift assignments (δ, ppm) for peptides **1** and **2** in H_2O:D_2O (1:9)

Peptide **1**		Peptide **2**	
[1]H no.	δ, D_2O:H_2O (1:9)	[1]H no.	δ, D_2O:H_2O (1:9)
2	7.94	2	7.94
3	4.43	3	4.43
5	8.56	5	8.48
6	4.26	6	4.29
7\`	2.22	7\`	2.19
7\`\`	2.16	7\`\`	1.78
8\`	2.38	8\`	2.35
10	4.5	8\`\`	2.29
11	8.77	10	4.83
14\`	1.85	11	8.17
15\`\`	1.32	13	4.2
16	7.97	14\`	1.87
60\`	1.77	14\`\`	1.74
77\`	2.04	15\`	1.26
77\`\`	2.15	15\`\`	1.35
78\`	2.56	16	8.01
82	2.32	77\`	2.11
87\`	1.49	81\`	2.04
87\`\`	1.62	81\`\`	1.95
88\`	3.01	87\`	1.49
88\`\`	3.46	87\`\`	1.52
		88\`	3.5
		88\`\`	2.93

1.2. NOE Build-up derived distances and *J* couplings

Interproton distances have been determined using the initial build-up rate approximation and NOESY spectra with mixing times 100, 200, 300, 400, 500, 600 and 700 ms.

Table S2. Interproton distances (Å) for peptide **1** derived from NOE build-ups in D_2O:H_2O (1:9) (δ in ppm)

No.	^1H_A	^1H_B	δ_A	δ_B	σ	R^2	Distance r_{AB}(Å)
1	11	2	8.77	7.94	8.01E-05	0.99	2.07
2	11	10	8.77	4.50	2.34E-05	0.98	2.54
3	2	10	7.94	4.50	4.43E-05	0.99	2.28
4	11	78`	8.77	2.56	1.98E-05	0.97	2.61
5	16	88`	7.97	3.01	2.07E-05	0.96	2.59
6	16	88``	7.97	3.46	2.13E-05	0.98	2.58
7	88``	87`	3.46	1.49	3.59E-05	0.98	2.37
8	88``	87``	3.46	1.62	2.19E-05	0.95	2.57
9	8`	60`	2.38	1.77	4.51E-05	0.97	2.28
10	2	3	7.95	4.43	2.01E-05	0.97	2.61
11	2	77`	7.95	2.04	1.93E-05	0.96	2.62
12	11	77`	8.77	2.04	2.88E-05	0.98	2.45
13	78`	77`	2.56	2.04	4.86E-05	0.95	2.25
14	16	8`	7.97	3.38	6.29E-05	0.99	2.15
15	16	82	7.97	2.32	3.91E-05	0.99	2.33
Ref.	88`	88``	3.01	3.46	0.000198	0.98	1.78

Table S3. Interproton distances (Å) for peptide **2** derived from NOE build-ups in D_2O:H_2O (1:9) (δ in ppm)

No.	^1H_A	^1H_B	δ_A	δ_B	σ	R^2	Distance r_{AB}(Å)
1	11	13	8.37	4.2	2.5E-05	0.99	3.01
2	11	10	8.37	4.83	3.07024E-05	0.94	2.91
3	11	2	8.37	7.94	8.1952E-05	0.95	2.47
4	16	88`	8.01	3.50	4.87576E-05	0.97	2.70
5	16	87`	8.01	1.49	3.5315E-05	0.99	2.85
6	16	88``	8.01	2.93	8.21197E-05	0.99	2.47
7	2	3	7.94	4.43	2.93719E-05	0.98	2.93
8	2	10	7.94	4.83	4.06727E-05	0.99	2.78
9	13	14``	4.2	1.74	5.98922E-05	0.98	2.61
10	13	87`	4.20	1.49	1.57269E-05	0.96	3.26
11	13	15``	4.20	1.35	7.86189E-06	0.98	3.66
12	13	15`	4.20	1.26	6.17974E-05	0.98	2.59
13	2	81``	7.94	1.95	3.58754E-05	0.95	2.84
14	2	81`	7.94	2.04	3.73568E-05	0.96	2.82
15	7`	8`	2.19	2.35	0.000115131	0.95	2.34
Ref.	88`	88``	3.50	2.93	0.000589811	0.97	1.78

Table S4. $^3J_{HH}$ coupling constants for Peptides **1** and **2** in D_2O:H_2O (1:9) (δ in ppm)

Peptide **1**					Peptide **2**				
^1H_A	^1H_B	δ_A	δ_B	$^3J_{HH}$ (Hz)	^1H_A	^1H_B	δ_A	δ_B	$^3J_{HH}$ (Hz)
2	3	7.94	4.43	6.5	2	3	7.94	4.43	7.3
5	6	8.56	4.26	8.9	11	10	8.37	4.83	6.8
11	10	8.77	4.5	7.0					

2. Monte Carlo Molecular Mechanics (MCMM) conformational search

The theoretical conformation ensembles of peptides **1** and **2** were identified by Monte Carlo conformational analysis using five (OPLS-2001, OPLS-2005, OPLS3e, AMBER,* and MMFF) force fields, each with the GB/SA solvation models for chloroform and water.[1] These conformational searches were performed using the Monte Carlo algorithm with intermediate torsion sampling with 50 000 Monte Carlo steps and an RMSD cut-off set to 2.0 Å. A Molecular Mechanics energy minimization was performed at each Monte Carlo step, as implemented in the Macromodel BatchMin V12.1 of the Schrödinger Package. Each conformation was energy minimized using Polak-Ribière type conjugate gradient (PRCG) with a maximum of 5000 steps. All conformations within 42 kJ/mol from the global minimum were saved. Results of all the different conformational searches are given in Table S5. All ensembles generated by the conformational searches were combined and elimination of redundant conformations was performed by comparison of heavy atom coordinates applying an RMSD cutoff set to 2.0 Å to give the final ensemble used for NAMFIS-analysis.

Table S5. Result of the MCMM conformational analysis. The number of conformations from each run using different force fields and solvent models are shown, along with the number of conformers in the combined conformational pool, following redundant conformation elimination.

	Force Field	Solvent		[a]Final
		H_2O	$CHCl_3$	ensemble
	AMBER*	85	161	
	MMFF	55	49	
Peptide **1**	OPLS_2005	489	22	92
	OPLS-2001	99	60	
	OPLS3e	625	39	
	AMBER*	188	118	
	MMFF	24	30	
Peptide **2**	OPLS_2005	583	65	75
	OPLS-2001	74	34	
	OPLS3e	180	77	

[a]Final ensemble obtained after redundant conformation elimination of the combined conformations.

3. X-ray crystal structure used for NAMFIS analysis

Table S6. Peptide **2** crystal structure from the Protein Data Bank (PDB)

	PDB code
Peptide **2**	2S35

4. NAMFIS analysis

NMR analysis of molecular flexibility in solution (NAMFIS) uses experimentally derived distances and dihedral angles (coupling constants) and fits them to back-calculated values of computationally generated conformations in order to identify the conformers present in solution, along with identifying their molar fractions.[1-2] For the NOE derived distances and ^3J-coupling derived dihedral angles, CH_2-to-H distances were averaged according to equation 1 and CH_3-to-H distances according to equation 2.

$$d_{average} = \left(\frac{(d_1^{-6}+d_2^{-6})}{2}\right)^{-(1/6)} \quad \text{Eq. 1}$$

$$d_{average} = \left(\frac{(d_1^{-6}+d_2^{-6}+d_3^{-6})}{3}\right)^{-(1/6)} \quad \text{Eq. 2}$$

The degree of matching of the population-weighted back-calculated data and the experimental values is expressed as RMSD error. The validation of NAMFIS ensemble analyses were performed using standard methods, that is, through evaluation of the reliability of conformational restraints by the additions of 10% random noise to the experimental data, and by the random removal of 10% of individual restrains, comparing the experimentally observed and back-calculated distances. No significant change in the output ensembles upon the above tests is indicative of a robust solution to the problem.

Table S7. Experimentally determined and back-calculated interproton distances (Å) of peptides **1** and **2** (NAMFIS output)

	Interproton distances Peptide **1**				Interproton distances Peptide **2**			
No.	^{1}H A	^{1}H B	Exp.$_{dist(Å)}$	Calc.$_{dist(Å)}$	^{1}H A	^{1}H B	Exp.$_{dist(Å)}$	Calc.$_{dist(Å)}$
1	11	2	2.07	2.24	11	13	3.01	3.10
2	11	10	2.54	2.88	11	10	2.91	2.67
3	2	10	2.28	2.60	11	2	2.47	2.44
4	11	78\`	2.61	2.44	16	88\`	2.70	2.63
5	16	88\`	2.59	2.52	16	87\`	2.85	2.71
6	16	88\`\`	2.58	2.55	16	88\`\`	2.47	2.66
7	88\`\`	87\`	2.37	2.43	2	3	2.93	2.66
8	88\`\`	87\`\`	2.57	2.63	2	10	2.78	3.18
9	8\`	60\`	2.28	2.38	13	14\`\`	2.61	2.49
10	2	3	2.61	2.73	13	87\`	3.26	3.33
11	2	77\`	2.62	2.81	13	15\`\`	3.66	3.34
12	11	77\`	2.45	2.64	13	15\`	2.59	2.84
13	78\`	77\`	2.25	2.55	2	81\`\`	2.84	2.95
14	16	8\`	2.15	2.40	2	81\`	2.82	2.89
15	16	82	2.33	2.65	7\`	8\`	2.34	2.44
			RMSD	0.21			RMSD	0.20

Table S8. Experimentally determined and back-calculated *J* Couplings of peptides **1** and **2** (NAMFIS output)

	Coupling constants Peptide **1**				Coupling constants Peptide **2**			
No.	^1H_A	^1H_B	Exp.$_{dist(Å)}$	Calc.$_{dist(Å)}$	^1H_A	^1H_B	Exp.$_{dist(Å)}$	Calc.$_{dist(Å)}$
1	2	3	6.5	6.2	2	3	7.3	7.3
2	5	6	8.9	8.8	11	10	6.8	7.5
3	11	10	7.0	7.2				
			RMSD	0.22			RMSD	0.52

Table S9. Results of the NAMFIS-analyses for peptides **1** and **2** in $D_2O:H_2O$ (1:9) solution

Peptide 1		Peptide 2	
Conf. No.	Popl. (%)	Conf. No.	Popl. (%)
1	34	1	18
2	26	2(2S35)	16
3	12	3	15
4	10	4	13
5	7	5	9
6	6	6	9
		7	9
		8	6
		9	2

5. MD analysis of peptides 1 and 2

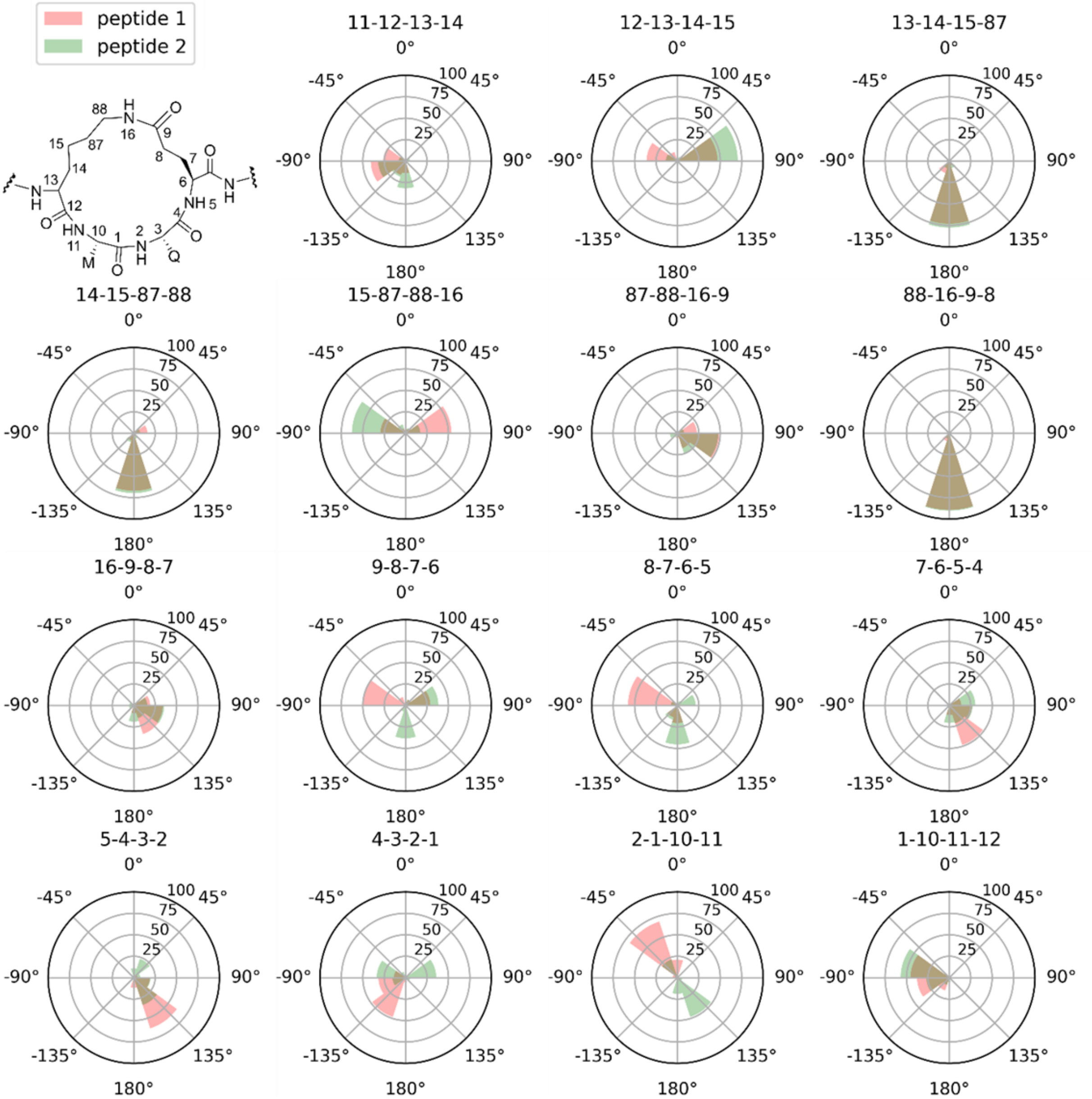

Figure S2: Selected torsional angles describing the macrocycle conformation of peptide **1** and **2** according to the MD simulations. The amide dihedral angles of the backbone are not shown as those are all in the trans conformation. The dihedral angles are displayed as pseudo-Newman projections, with the dihedral angle specified above each polar histogram. Every wedge covers 36° and the height represents the distribution of the specific angles in the MD trajectories. Red: dihedral angles of accessible for peptide **1** according to the MD trajectories. Green, dihedral angles accessible for peptide **2** according to the MD trajectories. Overlapping red and green regions appear in brownish color.

Table S10. Clusters using a cutoff of 0.7Å on the heavy atoms of the macrocycle and RMSD analysis of MD simulation of peptide **1**

MD		NAMFIS[a]			
cluster	Members (%)	Conformer 1 (34%)	Conformer 2 (26%)	Conformer 3 (12%)	Conformer 4 (10%)
**1**	**82.55**	1.21	0.64	1.27	1.63
**2**	**6.96**	1.47	0.72	1.38	1.70
**3**	**3.70**	0.77	1.11	1.33	1.82
**4**	**3.09**	1.39	1.19	1.20	0.86
**5**	**0.96**	1.26	1.19	1.25	1.71
**6**	**0.75**	1.59	0.69	1.56	1.70
**7**	**0.54**	0.95	0.80	1.20	1.46
**8**	**0.43**	0.92	1.50	0.96	1.70
**9**	**0.22**	1.53	1.01	1.39	1.77
**10**	**0.19**	1.29	0.99	1.41	1.34
**11**	**0.14**	1.61	1.33	1.02	0.70
**12**	**0.11**	1.14	0.90	1.00	1.47
**13**	**0.08**	1.08	1.26	1.47	2.00
**14**	**0.06**	1.13	1.03	1.43	1.53
**15**	**0.05**	1.68	0.90	1.65	1.87
**16**	**0.04**	1.26	0.98	0.92	1.39
**17**	**0.04**	0.83	1.22	1.47	1.79
**18**	**0.03**	1.24	1.38	1.06	0.81
**19**	**0.02**	1.57	1.32	1.35	1.83
**20**	**0.01**	1.79	1.43	1.45	0.92
**21**	**0.01**	1.43	0.83	1.43	1.49
**22**	**0.01**	0.72	1.44	0.83	1.35
**23**	**0.01**	1.36	1.01	1.02	1.11
**24**	**0.01**	1.46	1.14	1.73	1.72
**25**	**0.00**	1.14	1.20	1.35	1.85
**26**	**0.00**	1.15	0.98	1.69	1.92

[a]The RMSD (heavy atoms of macrocycle in Ångström) between the central structure of the clusters and the NAMFIS conformations.

Table S11. Clusters using a cutoff of 0.7Å on the heavy atoms of the macrocycle and RMSD analysis of MD simulation of peptide **2**

MD		NAMFIS[a]			
**cluster**	**Members (%)**	**Conformer 1 (18%)**	**Conformer 2 (16%, x-ray)**	**Conformer 3 (15%)**	**Conformer 4 (13%)**
**1**	54.10	1.18	0.91	1.18	1.16
**2**	28.61	1.28	1.21	1.61	1.54
**3**	7.42	1.65	0.61	1.37	1.34
**4**	2.71	0.74	1.26	1.01	0.96
**5**	2.39	1.67	1.28	1.78	1.76
**6**	1.44	1.06	1.48	1.75	1.67
**7**	1.07	1.43	0.72	0.91	0.89
**8**	0.90	1.52	0.83	1.48	1.42
**9**	0.39	1.57	0.96	1.12	1.10
**10**	0.22	1.43	0.84	1.20	1.15
**11**	0.21	1.44	1.03	1.08	1.09
**12**	0.18	1.85	0.88	1.69	1.65
**13**	0.10	1.18	1.13	1.00	0.98
**14**	0.05	1.02	1.04	1.25	1.18
**15**	0.04	1.64	1.26	1.76	1.71
**16**	0.04	0.59	1.59	1.19	1.15
**17**	0.04	0.81	1.53	1.66	1.58
**18**	0.03	1.32	0.80	0.99	0.92
**19**	0.02	1.51	1.21	1.81	1.77
**20**	0.01	1.59	0.70	1.21	1.18
**21**	0.01	1.56	1.31	1.68	1.64
**22**	0.01	1.44	1.14	1.08	1.09
**23**	0.01	1.15	1.18	1.54	1.44
**24**	0.00	0.77	1.50	1.58	1.51
**25**	0.00	1.16	1.05	0.74	0.61
**26**	0.00	1.97	1.04	1.57	1.54

[a]The RMSD (heavy atoms of macrocycle in Ångström) between the central structure of the clusters and the NAMFIS conformations.

Table S12. Clusters using a cutoff of 1A on the heavy atoms of the macrocycle and RMSD analysis of MD simulation of peptide **1**

MD		NAMFIS[a]			
**cluster**	**Members (%)**	**Conformer 1 (34%)**	**Conformer 2 (26%)**	**Conformer 3 (12%)**	**Conformer 4 (10%)**
**1**	96.22	1.21	0.64	1.27	1.63
**2**	2.41	1.48	1.31	1.25	0.80
**3**	1.20	0.76	1.27	0.98	1.68
**4**	0.10	1.71	0.90	1.63	1.85
**5**	0.05	0.91	1.23	1.59	1.94
**6**	0.01	1.26	0.89	1.46	1.67
**7**	0.01	1.67	1.43	1.44	1.90

[a]The RMSD (heavy atoms of macrocycle in Ångström) between the central structure of the clusters and the NAMFIS conformations.

Table S13. Clusters using a cutoff of 1A on the heavy atoms of the macrocycle and RMSD analysis of MD simulation of peptide **2**

MD		NAMFIS[a]			
**cluster**	**Members (%)**	**Conformer 1 (18%)**	**Conformer 2 (16%, x-ray)**	**Conformer 3 (15%)**	**Conformer 4 (13%)**
**1**	93.21	1.26	0.87	1.27	1.23
**2**	4.86	1.09	1.39	1.68	1.61
**3**	1.43	1.69	0.58	1.29	1.25
**4**	0.23	0.70	1.48	1.11	1.07
**5**	0.19	1.44	1.53	1.96	1.88
**6**	0.07	1.77	1.47	2.01	1.99
**7**	0.01	1.96	0.97	1.54	1.51

[a]The RMSD (heavy atoms of macrocycle in Ångström) between the central structure of the clusters and the NAMFIS conformations.

6. *NMR Spectra*

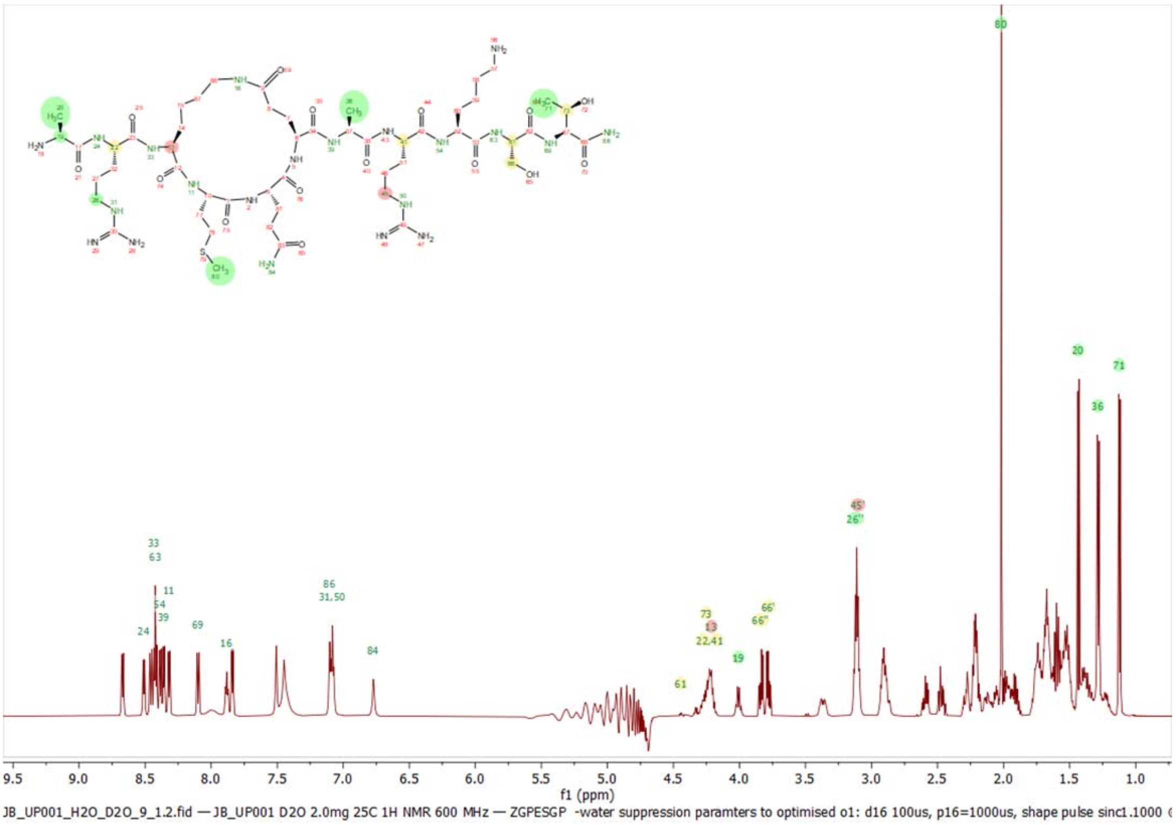

Figure S3: ^{1}H NMR Spectrum of Peptide **1**

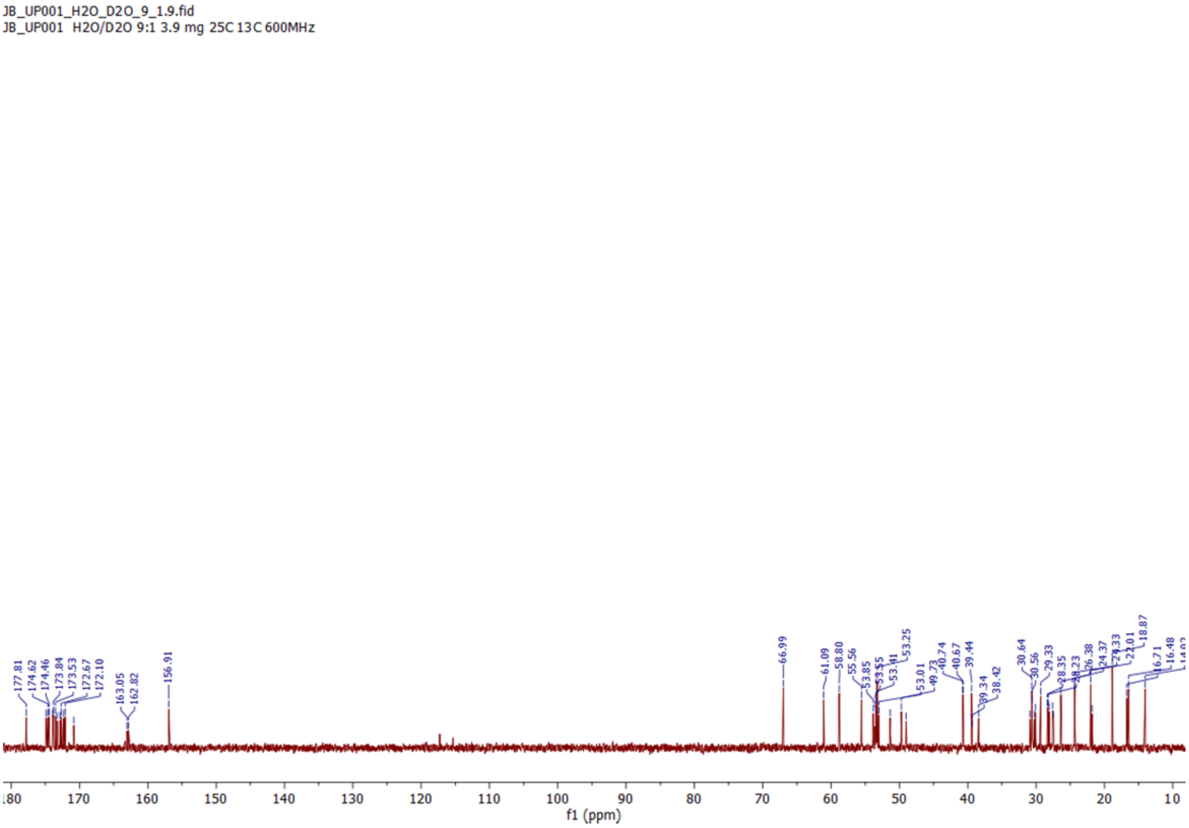

Figure S4: ^{13}C NMR Spectrum of Peptide **1**

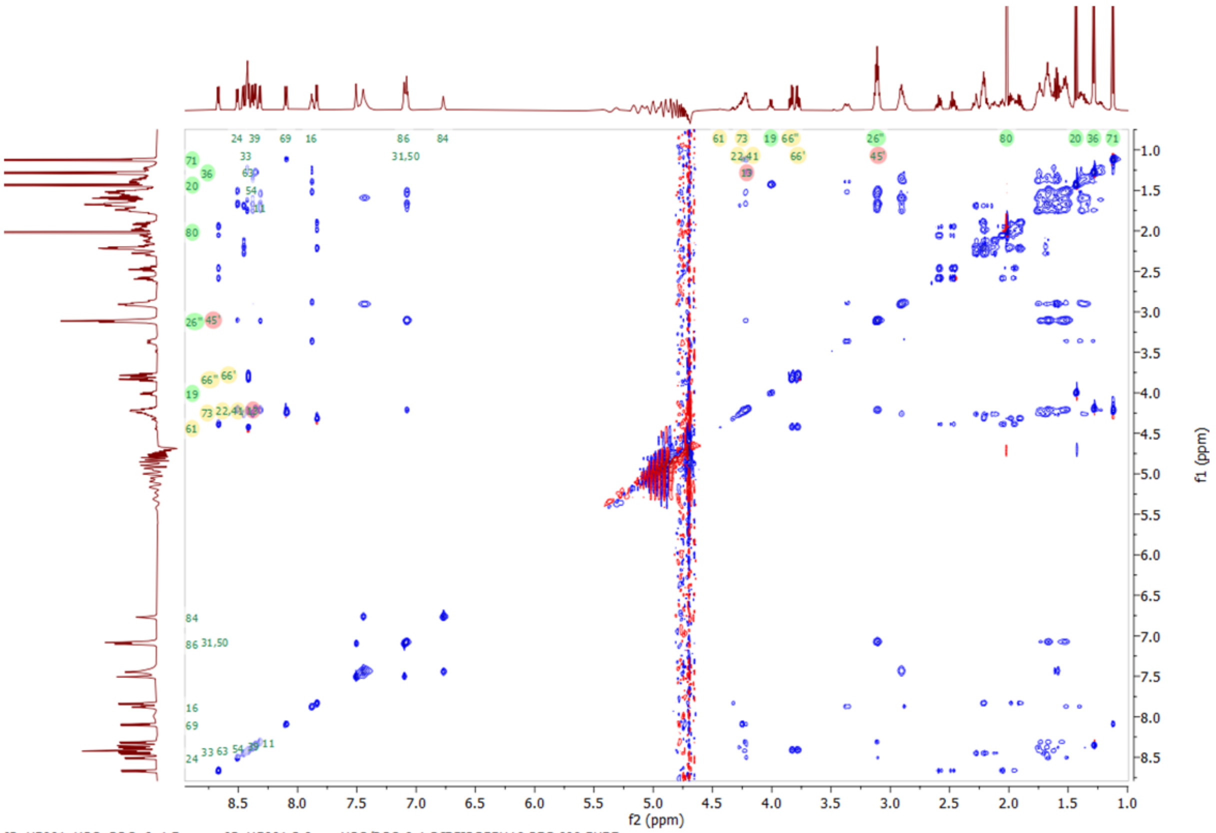

JB_UP001_H2O_D2O_9_1.5.ser — JB_UP001 3.9 mg H2O/D2O 9:1 DIPSI2GPPH19 25C 600 PURE

Figure S5: TOCSY Spectrum of Peptide **1**

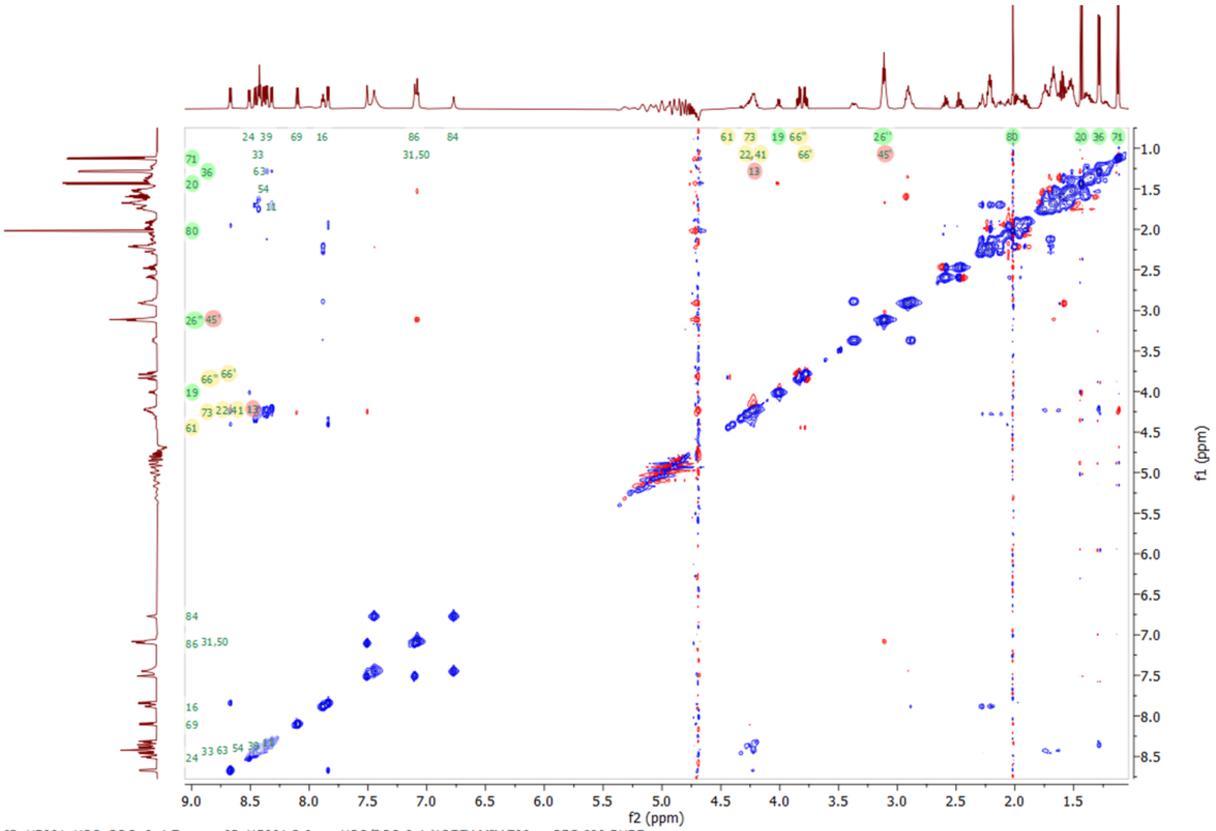

JB_UP001_H2O_D2O_9_1.7.ser — JB_UP001 3.9 mg H2O/D2O 9:1 NOESY MIX 700ms 25C 600 PURE

Figure S6: NOESY Spectrum of Peptide **1**

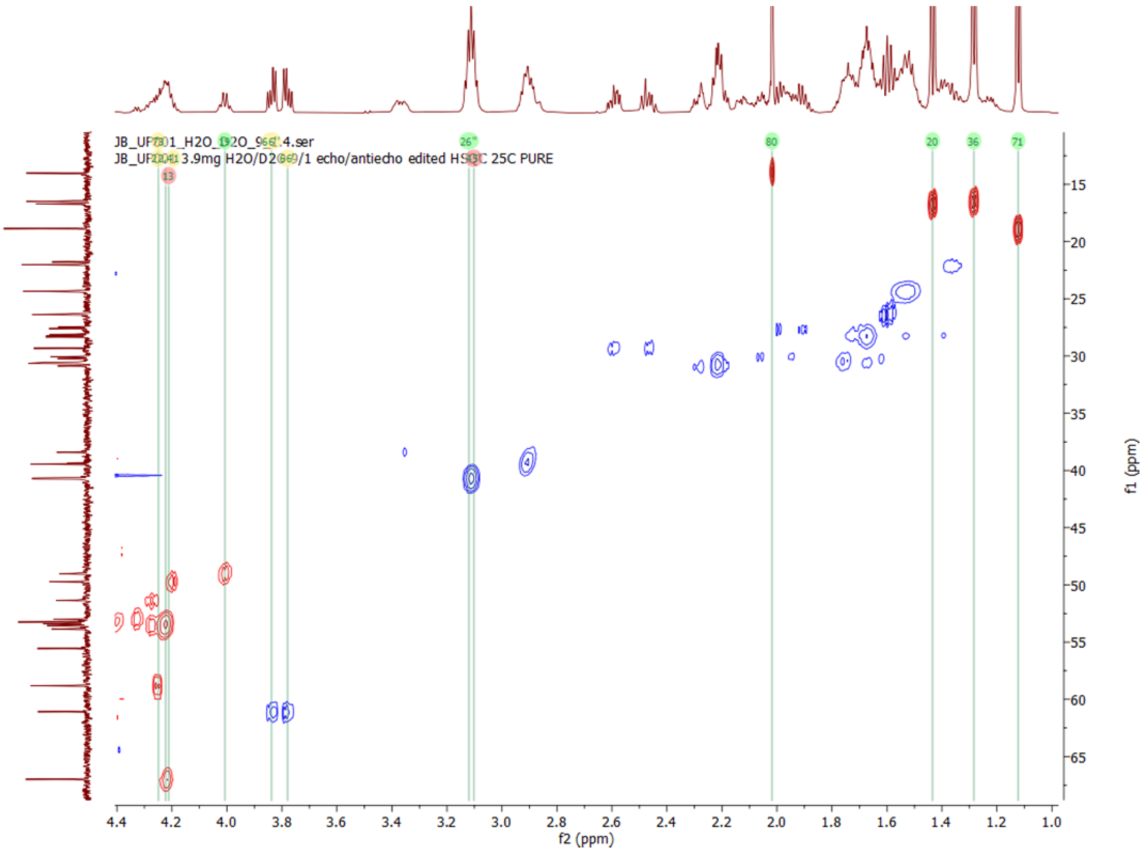

Figure S7: HSQC Spectrum of Peptide **1**

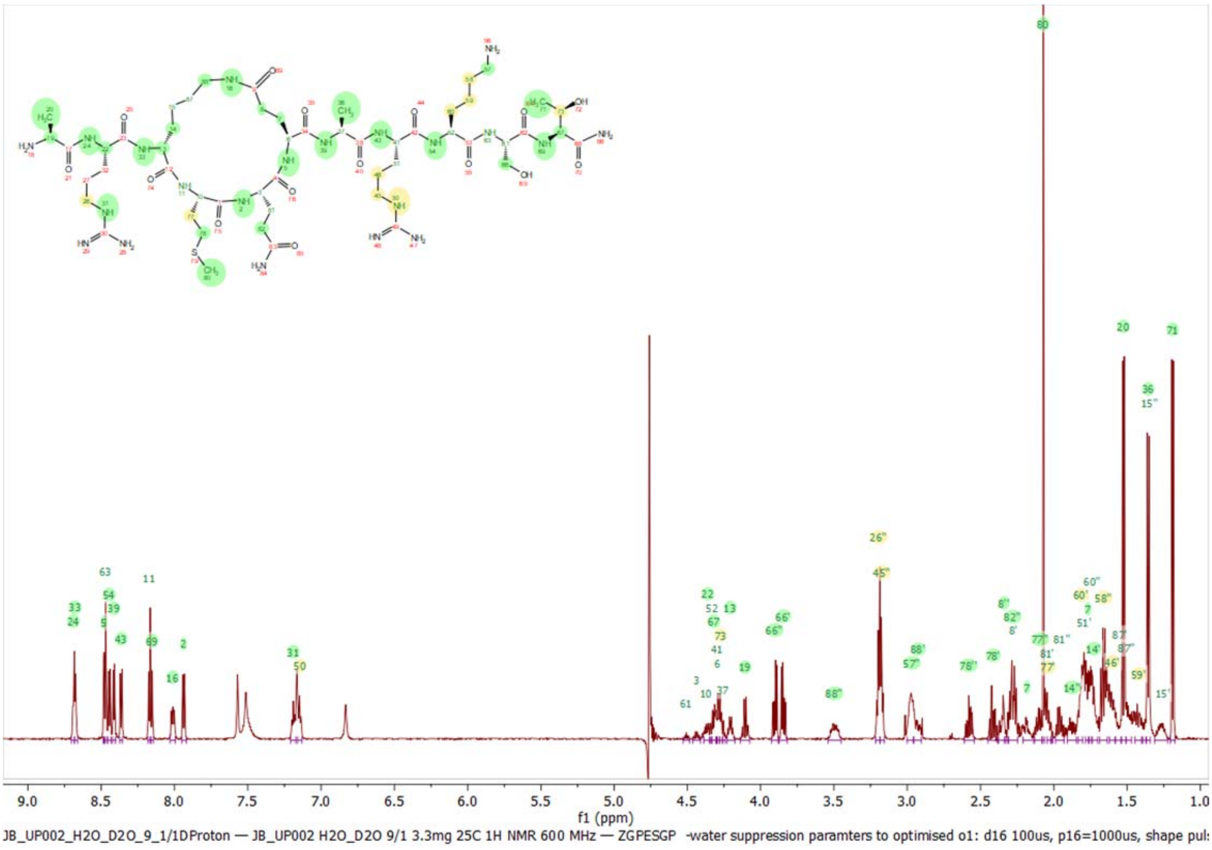

Figure S8: ^{1}H NMR Spectrum of Peptide **2**

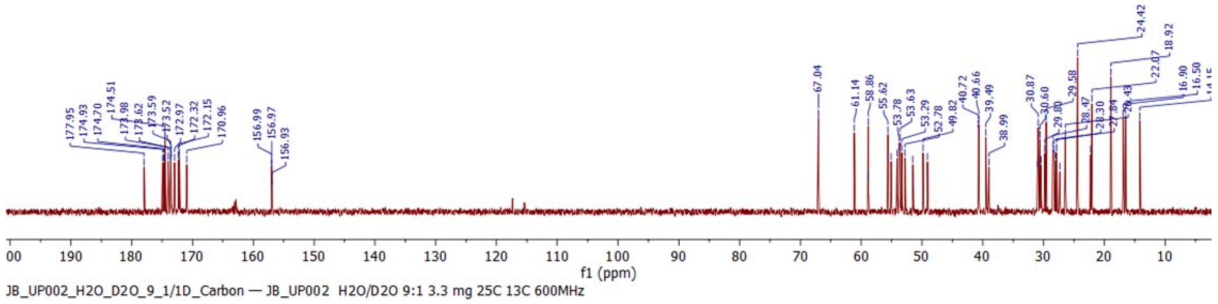

Figure S9: ^{13}C NMR Spectrum of Peptide **2**

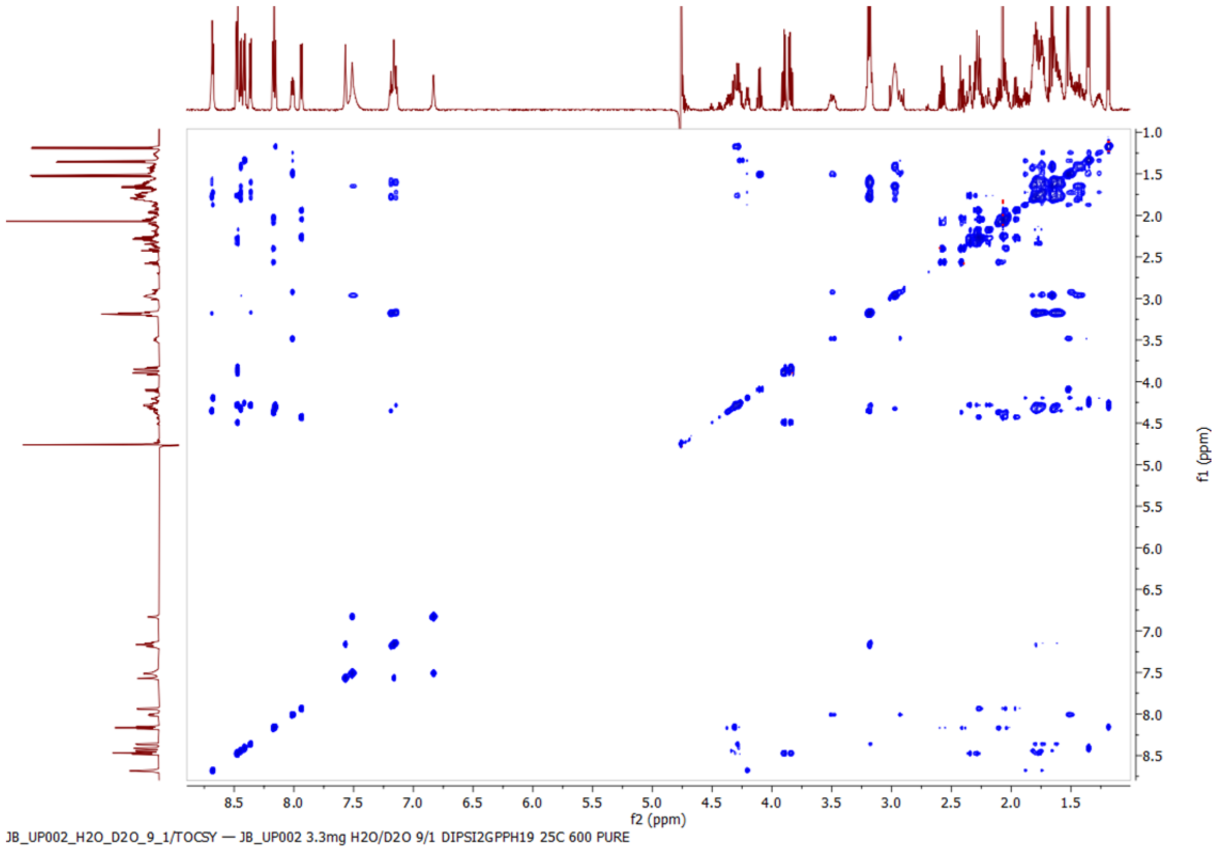

Figure S10: TOCSY Spectrum of Peptide **2**

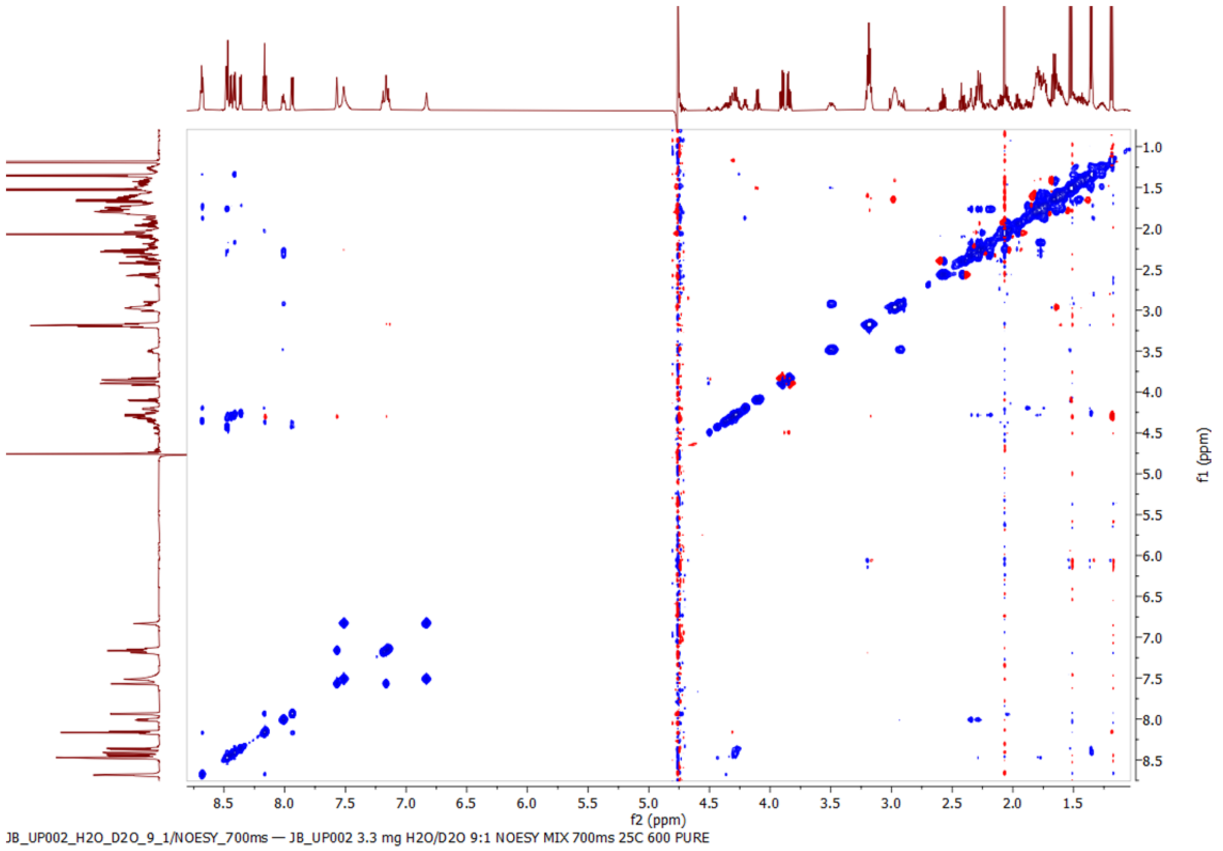

Figure S11: NOESY Spectrum of Peptide **2**

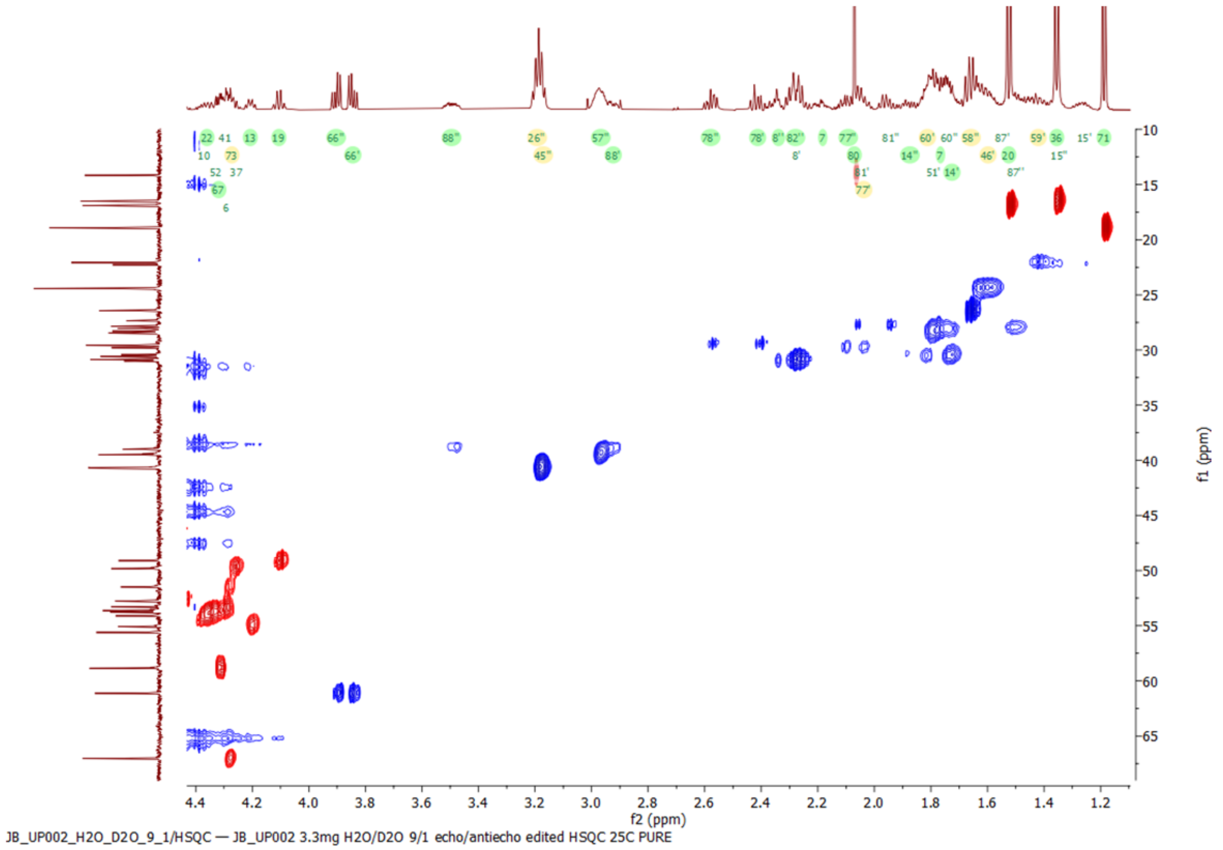

Figure S12: HSQC Spectrum of Peptide **2**

Coordinates of the Ensembles of Peptide 1 and 2

Peptide 1

JB_UP001_MMFF_WATER_23_26%

C	-8.26220	-61.02600	5.44190
N	-9.25560	-60.28420	4.83930
C	-10.64740	-60.72290	4.75470
C	-10.94470	-61.15490	3.29530
N	-12.04240	-61.97230	3.14900
C	-12.54100	-62.37650	1.83390
C	-13.11330	-63.80760	1.90380
C	-12.41930	-64.76890	0.93770
C	-10.95550	-64.99360	1.27490
C	-6.82040	-60.50990	5.19560
N	-6.62300	-60.39100	3.75300
C	-6.26680	-61.47870	2.97410
C	-6.59650	-61.29630	1.46800
C	-5.97550	-62.34450	0.53630
C	-6.61370	-63.74310	0.60780
N	-10.14490	-65.10820	0.16790
C	-6.79950	-57.41380	-1.16500
N	-9.11680	-58.29460	-0.95570
C	-8.29030	-57.19280	-1.51140
C	-8.78990	-55.83590	-1.03270
O	-5.95910	-57.66570	-2.03030
C	-5.18660	-57.79490	0.67970
C	-5.13830	-59.34140	0.82320
N	-6.49260	-57.36230	0.17620
O	-4.08030	-59.96890	0.86190
C	-4.51270	-55.03120	3.36910
C	-4.78520	-55.64430	1.99410
N	-1.35430	-55.20420	5.10310
N	-3.33170	-54.61290	6.07300
C	-2.69010	-55.04680	5.05000
N	-3.18730	-55.39740	3.84400
C	-4.88950	-57.17260	2.04920
N	-6.36640	-59.91920	1.04200
C	-13.63790	-61.38270	1.36480
O	-14.26140	-60.66100	2.14400
C	-16.37300	-61.47280	-0.07650
C	-15.09140	-60.82410	-0.59110
C	-15.14300	-59.27950	-0.51890
N	-13.89040	-61.41150	0.00700
O	-16.19800	-58.64660	-0.45500
C	-13.79780	-57.20180	-0.73960
C	-13.83830	-56.67840	-2.19200
N	-13.92370	-58.65850	-0.69540
O	-14.45570	-55.65540	-2.49530
C	-11.31760	-56.50050	2.18050
C	-12.63560	-56.81510	1.47500
N	-8.53210	-56.50740	2.86150
N	-8.44850	-58.74710	2.45350
C	-9.12140	-57.64390	2.46620
N	-10.37850	-57.59050	1.99530
C	-12.50890	-56.74540	-0.05130
C	-12.84860	-56.90400	-4.47430
C	-13.01850	-58.10370	-5.43790
N	-13.04420	-57.35670	-3.09160
O	-12.35010	-58.26370	-6.45680

N	-8.57690	-52.92240	-5.68600
C	-9.84480	-53.64610	-5.58380
C	-10.04560	-54.16620	-4.15980
C	-11.39440	-54.86690	-3.95550
C	-11.48450	-56.22190	-4.66510
C	-14.31160	-60.23320	-5.75440
C	-13.16140	-61.21760	-5.44010
N	-14.07280	-58.93420	-5.10510
O	-12.81390	-62.12940	-6.18790
O	-15.73240	-59.29490	-7.50830
C	-14.60290	-60.14310	-7.25680
C	-11.46080	-61.74890	-3.66060
C	-11.82030	-62.89620	-2.69450
N	-12.64790	-61.06110	-4.17160
O	-10.98580	-63.59980	-2.12490
C	-9.63230	-60.05340	-4.03030
O	-11.25990	-59.71080	-2.28670
C	-10.50770	-60.73610	-2.98250
O	-5.87520	-62.53700	3.46410
O	-8.50150	-61.97420	6.19070
O	-10.27510	-60.77130	2.33350
C	-6.62310	-59.15970	5.89770
C	-5.18690	-58.63770	5.85910
S	-4.06170	-59.63110	6.89830
C	-2.55420	-58.65560	6.64750
C	-11.60060	-59.58880	5.15710
C	-11.49120	-59.20800	6.63430
C	-12.05640	-60.26510	7.56200
N	-11.84500	-60.01380	8.88380
O	-12.66380	-61.26510	7.19040
N	-13.15760	-63.11920	-2.49630
C	-8.12730	-63.74110	0.38860
C	-8.69790	-65.15290	0.27150
O	-10.54660	-65.10140	2.42860
H	-8.96240	-59.60040	4.14170
H	-10.78700	-61.60300	5.39080
H	-12.69660	-62.02960	3.92530
H	-11.71640	-62.31060	1.11400
H	-14.18430	-63.80560	1.66490
H	-13.02900	-64.20870	2.92200
H	-12.52730	-64.39930	-0.08290
H	-12.91180	-65.74770	0.97940
H	-6.13670	-61.26990	5.58820
H	-7.15030	-59.65670	3.27070
H	-7.68570	-61.38180	1.40850
H	-4.90430	-62.44770	0.74690
H	-6.05570	-61.99300	-0.50090
H	-6.38830	-64.20930	1.57320
H	-6.13770	-64.37110	-0.15570
H	-10.51850	-64.78770	-0.73000
H	-8.52670	-59.13110	-0.90160
H	-9.31680	-58.07300	0.02660
H	-8.37390	-57.22970	-2.60340
H	-8.73940	-55.73210	0.05490
H	-8.18270	-55.03160	-1.46170
H	-9.82840	-55.67540	-1.33450
H	-4.41920	-57.51230	-0.05050
H	-7.25870	-57.27910	0.84230
H	-5.27880	-55.36780	4.07680
H	-4.57820	-53.93980	3.29470

H	-3.99630	-55.35420	1.28930
H	-5.72470	-55.22520	1.61490
H	-1.01770	-56.05580	4.66110
H	-0.99780	-55.15920	6.05540
H	-4.33190	-54.57070	5.83880
H	-2.48440	-55.34000	3.10840
H	-3.95040	-57.59000	2.43360
H	-5.66740	-57.46020	2.76600
H	-7.12090	-59.26980	1.26720
H	-17.24060	-61.13340	-0.65240
H	-16.31720	-62.56350	-0.15800
H	-16.56030	-61.22300	0.97280
H	-15.01590	-61.05090	-1.66280
H	-13.42610	-62.11710	-0.55870
H	-14.66430	-56.76020	-0.23170
H	-13.10950	-59.24140	-0.88580
H	-11.51220	-56.37300	3.25150
H	-10.90180	-55.56150	1.79770
H	-13.39020	-56.08840	1.79980
H	-12.99260	-57.80520	1.78360
H	-9.13490	-55.86290	3.35930
H	-7.70280	-56.67670	3.41840
H	-9.10450	-59.47220	2.08360
H	-10.82290	-58.49060	1.83090
H	-12.27600	-55.71210	-0.33740
H	-11.67170	-57.36200	-0.39210
H	-13.64380	-56.19580	-4.73850
H	-12.44430	-58.10650	-2.74490
H	-8.51520	-52.51580	-6.62760
H	-8.62180	-52.09860	-5.07430
H	-9.82750	-54.47310	-6.29950
H	-10.66620	-52.97730	-5.86290
H	-9.99360	-53.32390	-3.45750
H	-9.23140	-54.85120	-3.89330
H	-12.20380	-54.21090	-4.29750
H	-11.53960	-55.01240	-2.87960
H	-11.30580	-56.07430	-5.73580
H	-10.68610	-56.88040	-4.29990
H	-15.20670	-60.63060	-5.25800
H	-14.54210	-58.74870	-4.22260
H	-15.55700	-58.47150	-7.00400
H	-14.84240	-61.13170	-7.66230
H	-13.75400	-59.75770	-7.82830
H	-10.95210	-62.24360	-4.49770
H	-12.97900	-60.29220	-3.59920
H	-10.23730	-59.46330	-4.72600
H	-8.93180	-59.35950	-3.55980
H	-9.05100	-60.78440	-4.60060
H	-10.57260	-59.17550	-1.81250
H	-9.86610	-61.23440	-2.24690
H	-6.96820	-59.22590	6.93800
H	-7.26250	-58.40510	5.42020
H	-5.17550	-57.60990	6.23720
H	-4.80610	-58.62470	4.83370
H	-1.72710	-59.12900	7.18300
H	-2.30140	-58.61080	5.58500
H	-2.68870	-57.64460	7.03970
H	-12.63690	-59.86350	4.92530
H	-11.37990	-58.69910	4.55470
H	-12.06510	-58.28860	6.80340

H	-10.45050	-59.00450	6.90990
H	-12.25500	-60.62060	9.58600
H	-11.50080	-59.11870	9.21680
H	-13.42490	-64.01350	-2.10100
H	-13.79290	-62.78110	-3.21190
H	-8.37440	-63.17100	-0.51570
H	-8.62330	-63.23800	1.22610
H	-8.32140	-65.64830	-0.62910
H	-8.42000	-65.75590	1.14260

JB_UP001_OPLS_2005_CHCl3_29_10%

C	-9.06660	-60.68400	3.26590
N	-9.55610	-60.59170	4.50240
C	-10.50870	-61.41790	5.24090
C	-11.69110	-61.98080	4.42220
N	-11.54530	-63.19580	3.87810
C	-12.61400	-64.00520	3.29360
C	-13.15350	-63.48230	1.93200
C	-12.14400	-62.85660	0.94540
C	-11.11030	-63.82800	0.38840
C	-7.90920	-59.72930	2.93370
N	-6.87240	-60.53700	2.29900
C	-5.97940	-61.27730	2.96390
C	-5.14480	-62.26960	2.12740
C	-5.69110	-63.70950	2.27090
C	-7.13380	-63.94420	1.78180
N	-9.82650	-63.46990	0.45410
C	-0.10480	-63.48380	3.05020
N	-0.49880	-64.83250	1.03350
C	0.43520	-64.58610	2.13390
C	1.82040	-64.21410	1.59100
O	0.10090	-63.49400	4.26320
C	-1.51710	-61.41140	3.04530
C	-2.90490	-61.25840	2.41560
N	-0.83830	-62.55250	2.44290
O	-3.19430	-60.25270	1.76970
C	0.29340	-60.04700	5.39230
C	0.60650	-60.12540	3.88750
N	-0.43250	-62.99470	7.48290
N	-0.47010	-60.79550	8.15510
C	-0.28390	-61.65220	7.26410
N	0.05730	-61.36010	5.96710
C	-0.64350	-60.13380	2.98500
N	-3.76020	-62.27290	2.58400
C	-12.31650	-65.50560	3.45060
O	-12.38100	-66.01590	4.56650
C	-11.59070	-68.12710	0.89910
C	-11.65990	-67.65430	2.36000
C	-10.32530	-67.99690	3.05410
N	-11.96540	-66.21660	2.36850
O	-10.07460	-69.15210	3.38990
C	-8.37750	-66.97510	4.23020
C	-8.30050	-65.54360	4.78710
N	-9.48990	-66.98570	3.29600
O	-9.08080	-64.66950	4.39090
C	-4.57720	-67.75880	4.07170
C	-6.01360	-68.03160	4.54810
N	-2.92200	-64.47570	4.39930
N	-2.76020	-65.88880	2.60350
C	-3.24660	-65.62110	3.72260

N	-4.13830	-66.41170	4.41440
C	-7.07760	-67.49660	3.56730
C	-7.05560	-63.97910	6.23420
C	-5.56330	-63.84180	6.53250
N	-7.35160	-65.28920	5.68620
O	-4.84070	-64.83720	6.64900
N	-7.55920	-61.90170	10.26730
C	-8.03500	-63.13670	10.86580
C	-8.71400	-64.05920	9.83850
C	-7.80960	-64.52520	8.68070
C	-7.97790	-63.65160	7.42310
C	-3.84660	-62.14200	7.18190
C	-3.86660	-62.24300	8.71980
N	-5.12800	-62.59390	6.66890
O	-4.91610	-62.08630	9.34810
O	-4.89180	-59.96550	6.57110
C	-3.66140	-60.67500	6.71400
C	-2.54340	-62.76580	10.75390
C	-2.16070	-61.45270	11.46650
N	-2.71630	-62.55130	9.31990
O	-2.40120	-61.29760	12.65810
C	-1.83750	-65.22110	10.43760
O	-0.22640	-63.47340	10.25410
C	-1.42370	-63.82820	10.93650
O	-5.84060	-61.20150	4.18840
O	-9.45530	-61.48680	2.42050
O	-12.72890	-61.32920	4.31350
C	-8.39690	-58.66260	1.93010
C	-9.31270	-57.60260	2.56320
S	-9.81900	-56.30390	1.40500
C	-10.86910	-55.30890	2.49870
C	-10.96880	-60.62510	6.49700
C	-9.95470	-60.47050	7.66330
C	-8.65000	-59.73320	7.33480
N	-7.57050	-60.05180	8.03380
O	-8.59540	-58.91310	6.42230
N	-1.56880	-60.49060	10.76420
C	-7.36220	-63.69810	0.28300
C	-8.73820	-64.19530	-0.18960
O	-11.47910	-64.89830	-0.09170
H	-9.14180	-59.85580	5.07400
H	-9.94930	-62.28500	5.58870
H	-10.65190	-63.66290	4.01780
H	-13.46120	-63.88560	3.97350
H	-13.89430	-62.71170	2.14880
H	-13.71820	-64.26950	1.43000
H	-11.66420	-61.99240	1.39980
H	-12.69330	-62.46050	0.09120
H	-7.51630	-59.25020	3.83370
H	-6.95220	-60.67950	1.30540
H	-5.15840	-61.97280	1.07720
H	-5.63180	-64.01200	3.31780
H	-5.03110	-64.39410	1.73640
H	-7.82740	-63.35830	2.37900
H	-7.40390	-64.97630	1.98290
H	-9.60580	-62.63480	0.99610
H	-0.20740	-65.62440	0.48040
H	-1.39300	-65.12960	1.44590
H	0.51600	-65.49870	2.72790
H	1.78160	-63.31340	0.97680

H	2.52210	-64.02780	2.40540
H	2.23560	-65.01650	0.98120
H	-1.72570	-61.63590	4.09040
H	-1.00730	-62.76110	1.46420
H	-0.55750	-59.39220	5.58430
H	1.14820	-59.61580	5.91530
H	1.24550	-60.98060	3.66610
H	1.20530	-59.25240	3.62380
H	-0.21330	-63.28570	8.44380
H	0.09480	-63.59290	6.85870
H	-0.25660	-59.86440	7.80780
H	0.15600	-62.14680	5.30920
H	-1.25320	-59.26620	3.24330
H	-0.33640	-59.96920	1.95120
H	-3.45030	-63.08860	3.11020
H	-11.39530	-69.19900	0.84230
H	-12.53070	-67.93980	0.37970
H	-10.79900	-67.61750	0.34760
H	-12.45780	-68.19030	2.87760
H	-11.90150	-65.73440	1.47360
H	-8.64170	-67.63150	5.06220
H	-9.82500	-66.06470	3.04490
H	-4.49190	-67.93540	2.99920
H	-3.89070	-68.45550	4.55480
H	-6.14610	-67.62200	5.54800
H	-6.15890	-69.10640	4.66970
H	-1.93290	-64.25150	4.33920
H	-3.18600	-64.47340	5.37890
H	-3.10060	-66.77210	2.24040
H	-4.37120	-66.10100	5.36010
H	-6.63570	-66.70300	2.96920
H	-7.31220	-68.29170	2.85820
H	-7.26170	-63.24230	5.45320
H	-6.71120	-66.02120	5.95140
H	-7.33420	-61.26940	11.02700
H	-6.63500	-62.10480	9.86360
H	-8.75270	-62.88980	11.65010
H	-7.20210	-63.64710	11.35300
H	-9.62440	-63.59610	9.45520
H	-9.05320	-64.94450	10.37850
H	-8.06830	-65.55270	8.42300
H	-6.77030	-64.54910	9.01130
H	-9.01710	-63.69620	7.09920
H	-7.79530	-62.61870	7.69830
H	-3.03970	-62.76200	6.78980
H	-5.79980	-61.84700	6.56230
H	-5.25670	-60.20680	5.71790
H	-3.15370	-60.66080	5.74950
H	-3.01410	-60.12900	7.40160
H	-3.46830	-63.13400	11.20370
H	-1.87560	-62.61510	8.74860
H	-2.04070	-65.22390	9.36620
H	-1.05360	-65.95490	10.62740
H	-2.73910	-65.56560	10.94480
H	0.10460	-62.66620	10.63230
H	-1.19010	-63.90980	12.00050
H	-8.90920	-59.13010	1.08700
H	-7.53090	-58.14970	1.50970
H	-8.80650	-57.13220	3.40760
H	-10.21500	-58.07010	2.96000

H	-11.27410	-54.45410	1.95670
H	-11.70230	-55.90300	2.87520
H	-10.29530	-54.93710	3.34790
H	-11.85020	-61.11670	6.91200
H	-11.32030	-59.63860	6.18970
H	-9.72710	-61.45340	8.07300
H	-10.43530	-59.92150	8.47350
H	-6.67120	-59.64840	7.78980
H	-7.63780	-60.71650	8.81120
H	-1.34610	-59.62110	11.21760
H	-1.31200	-60.65480	9.78410
H	-6.59170	-64.21880	-0.28770
H	-7.25280	-62.63920	0.04830
H	-8.82720	-64.06990	-1.26990
H	-8.83640	-65.26490	0.00770

JB_UP001_OPLS_2005_WATER_34_7%

C	-9.00280	-60.88150	4.93060
N	-9.59650	-60.53900	3.78000
C	-10.93830	-59.97780	3.61810
C	-12.03080	-61.06940	3.59610
N	-11.63610	-62.33720	3.45390
C	-12.46050	-63.53800	3.52690
C	-13.21360	-63.82160	2.19500
C	-12.42360	-63.66850	0.87280
C	-11.16900	-64.53260	0.78470
C	-7.49370	-61.20340	4.88590
N	-6.94860	-61.09800	3.52850
C	-5.96340	-61.84020	3.01890
C	-5.54340	-61.53070	1.57910
C	-5.20620	-62.76280	0.70490
C	-6.28330	-63.86870	0.68100
N	-10.07980	-63.95030	0.27420
C	-4.09580	-57.63990	3.08480
N	-2.89500	-56.60190	4.96530
C	-4.06160	-57.40770	4.60390
C	-5.34510	-56.74670	5.13200
O	-4.99430	-57.16340	2.38930
C	-2.95210	-58.71650	1.15620
C	-4.01830	-59.74670	0.73100
N	-3.08260	-58.35000	2.57170
O	-4.48820	-59.72370	-0.40680
C	0.33140	-60.95940	1.29840
C	-1.09900	-60.51980	1.64000
N	1.93300	-63.58910	3.33960
N	2.90810	-61.61550	2.70410
C	1.92090	-62.38370	2.69090
N	0.71740	-62.13190	2.06910
C	-1.52440	-59.24810	0.87550
N	-4.41730	-60.61680	1.66370
C	-11.65580	-64.68540	4.16460
O	-10.48430	-64.50720	4.50620
C	-12.98140	-67.98180	5.28840
C	-11.83220	-66.96730	5.17270
C	-10.57790	-67.69810	4.65370
N	-12.29840	-65.84390	4.35760
O	-9.91680	-68.38030	5.43780
C	-9.01790	-68.16850	2.76920
C	-7.79020	-67.25330	3.00680
N	-10.20890	-67.55630	3.37290

O	-7.07570	-66.87970	2.07620
C	-8.69220	-69.59960	-0.90400
C	-8.34740	-69.39070	0.57860
N	-9.14120	-70.92650	-3.38710
N	-7.18790	-72.09780	-3.19480
C	-7.98470	-71.25510	-2.72570
N	-7.81870	-70.58830	-1.52490
C	-9.29670	-68.38680	1.26030
C	-6.58880	-65.90330	4.76420
C	-5.15150	-66.30930	4.38950
N	-7.57590	-66.86410	4.27050
O	-4.76280	-67.46490	4.57540
N	-9.67810	-64.43330	10.11820
C	-9.54660	-64.42820	8.67260
C	-8.24140	-65.12350	8.26300
C	-8.07740	-65.17200	6.73890
C	-6.73900	-65.79200	6.29580
C	-3.04350	-65.58620	3.29160
C	-2.14260	-64.34970	3.48490
N	-4.37500	-65.36530	3.84940
O	-1.29840	-64.05100	2.63520
O	-1.91410	-66.57220	1.30860
C	-3.15860	-66.16040	1.85330
C	-1.44540	-62.54630	5.04190
C	-0.62420	-63.13140	6.20800
N	-2.32090	-63.62680	4.59740
O	-1.13710	-63.35000	7.30490
C	-1.51040	-60.10470	5.88260
O	-3.18850	-60.98130	4.43460
C	-2.32280	-61.35070	5.49840
O	-5.35590	-62.69510	3.66960
O	-9.61820	-60.91420	5.99530
O	-13.21700	-60.76020	3.71560
C	-6.64690	-60.34420	5.85650
C	-6.56100	-60.89050	7.28880
S	-5.50280	-62.35490	7.45820
C	-5.45120	-62.48580	9.26630
C	-10.98300	-59.22990	2.25890
C	-10.14600	-57.92690	2.17950
C	-8.62830	-58.11170	2.05670
N	-7.86440	-57.05890	2.30900
O	-8.13610	-59.20110	1.76590
N	0.64410	-63.44450	5.97590
C	-7.69070	-63.38100	0.29150
C	-8.73610	-64.50190	0.35720
O	-11.17710	-65.68650	1.21580
H	-9.01460	-60.50930	2.95240
H	-11.15770	-59.28550	4.43340
H	-10.63860	-62.50400	3.45630
H	-13.23410	-63.34180	4.27280
H	-14.06550	-63.14310	2.14240
H	-13.65830	-64.81680	2.22720
H	-12.15760	-62.62010	0.73180
H	-13.06890	-63.92880	0.03350
H	-7.41000	-62.24920	5.18530
H	-7.31840	-60.36060	2.93120
H	-6.36210	-60.99570	1.09600
H	-4.26580	-63.19490	1.04740
H	-5.02440	-62.43470	-0.31890
H	-6.32770	-64.34410	1.66110

H	-5.97070	-64.65440	-0.00810
H	-10.17620	-63.01540	-0.09880
H	-2.04400	-57.03570	4.62620
H	-2.92420	-55.71240	4.47820
H	-3.97270	-58.38570	5.07780
H	-5.48040	-55.74240	4.72860
H	-6.22640	-57.32830	4.85870
H	-5.33210	-56.67230	6.21950
H	-3.10380	-57.81680	0.55610
H	-2.42420	-58.74750	3.22610
H	1.02160	-60.14060	1.50120
H	0.41790	-61.19240	0.23640
H	-1.16900	-60.33830	2.71140
H	-1.78100	-61.34180	1.42060
H	1.43590	-64.33840	2.86880
H	2.86560	-63.93240	3.54760
H	2.75550	-60.75030	2.18480
H	-0.02570	-62.83630	2.16600
H	-0.81030	-58.45160	1.08730
H	-1.43250	-59.43920	-0.19500
H	-3.96520	-60.54650	2.57090
H	-12.70070	-68.82460	5.92190
H	-13.86660	-67.52770	5.73420
H	-13.26390	-68.38350	4.31420
H	-11.59990	-66.58560	6.16910
H	-13.24380	-65.90540	4.00880
H	-8.82540	-69.13700	3.23520
H	-10.74680	-66.92150	2.79010
H	-8.61190	-68.65880	-1.45070
H	-9.72740	-69.93390	-0.98830
H	-7.31570	-69.04890	0.66550
H	-8.39950	-70.34560	1.10400
H	-9.13040	-71.09180	-4.38850
H	-9.49280	-69.99160	-3.21550
H	-6.35890	-72.29640	-2.62870
H	-6.91100	-70.66780	-1.06940
H	-9.26870	-67.43790	0.72460
H	-10.31450	-68.76050	1.14080
H	-6.81170	-64.93510	4.31170
H	-8.20870	-67.25620	4.95690
H	-8.95430	-63.84660	10.52270
H	-10.54320	-63.96410	10.37410
H	-9.56320	-63.39930	8.30690
H	-10.40310	-64.94000	8.22960
H	-8.22180	-66.14160	8.65570
H	-7.38860	-64.60380	8.70010
H	-8.16110	-64.16420	6.33200
H	-8.90890	-65.73780	6.32040
H	-5.92420	-65.18520	6.69300
H	-6.62040	-66.77770	6.74920
H	-2.55940	-66.35490	3.89760
H	-4.75990	-64.42680	3.75130
H	-1.34360	-65.80620	1.31160
H	-3.60360	-65.42040	1.18830
H	-3.82760	-67.02210	1.84400
H	-0.77270	-62.21280	4.25300
H	-3.00180	-63.95570	5.26730
H	-0.90660	-59.74320	5.05030
H	-2.16050	-59.29270	6.20600
H	-0.83360	-60.31330	6.71140

H	-3.78620	-61.71990	4.31710
H	-2.93830	-61.64040	6.35070
H	-7.05910	-59.33490	5.88800
H	-5.63010	-60.22660	5.48300
H	-7.55250	-61.11440	7.68330
H	-6.14680	-60.11150	7.93000
H	-4.88200	-63.36570	9.56630
H	-4.97620	-61.60620	9.70150
H	-6.45760	-62.57110	9.67650
H	-12.01690	-58.94740	2.05280
H	-10.70870	-59.90250	1.44400
H	-10.36600	-57.30060	3.04500
H	-10.46730	-57.35950	1.30530
H	-6.84840	-57.12850	2.25360
H	-8.27890	-56.16910	2.54750
H	1.21260	-63.84660	6.70590
H	1.04780	-63.32510	5.04370
H	-7.67140	-62.94520	-0.70850
H	-8.00330	-62.58740	0.96980
H	-8.57730	-65.23640	-0.43380
H	-8.63700	-65.02240	1.30910

JB_UP001_OPLS_CHCl3_47_12%

C	-8.64540	-59.83820	4.22430
N	-9.58770	-60.76250	4.04300
C	-10.86370	-60.46880	3.40640
C	-11.87510	-61.57540	3.71140
N	-11.45500	-62.83780	3.60730
C	-12.32320	-64.00260	3.71460
C	-12.83280	-64.51180	2.34450
C	-12.29770	-63.85160	1.05620
C	-10.84960	-64.19860	0.71080
C	-7.30780	-60.34540	4.73180
N	-6.61930	-60.77590	3.53420
C	-5.90610	-61.89010	3.41460
C	-5.46870	-62.23770	1.99410
C	-5.89990	-63.65960	1.59220
C	-7.38640	-63.79580	1.23280
N	-10.23460	-63.43260	-0.19110
C	-0.61820	-63.72760	-0.58320
N	-1.09850	-63.14480	-2.87210
C	-0.62130	-64.24640	-2.01330
C	0.75400	-64.75070	-2.45380
O	0.12980	-64.17700	0.28220
C	-1.83540	-62.10670	0.85750
C	-3.34730	-61.97290	0.79070
N	-1.47510	-62.73370	-0.39540
O	-3.88740	-61.80540	-0.30250
C	0.89180	-61.25130	2.53080
C	0.37480	-60.72770	1.18320
N	0.10710	-62.84060	4.93060
N	-0.15250	-64.62040	3.49480
C	0.25000	-63.42740	3.66200
N	0.77870	-62.69830	2.57760
C	-1.15790	-60.73450	1.02650
N	-4.02720	-62.12650	1.92470
C	-11.51990	-65.03610	4.49290
O	-11.86910	-65.38720	5.61650
C	-8.25040	-66.60090	3.84510
C	-9.22100	-65.82590	4.73220

C	-8.60790	-64.55560	5.33000
N	-10.36370	-65.40640	3.95210
O	-8.78470	-63.46500	4.77120
C	-7.59240	-63.62990	7.40970
C	-6.14120	-63.88350	7.81220
N	-7.97660	-64.69090	6.49330
O	-5.82520	-64.03130	8.99210
C	-10.95750	-64.15070	9.01720
C	-9.95130	-63.20080	8.36340
N	-10.01040	-66.87250	9.90890
N	-10.72220	-67.65360	7.87220
C	-10.60310	-66.65730	8.65340
N	-11.04170	-65.37930	8.24240
C	-8.50890	-63.64380	8.64230
C	-3.93340	-64.58250	6.97410
C	-2.85930	-63.51670	6.80310
N	-5.25460	-63.97470	6.82020
O	-1.66670	-63.78230	6.93340
N	-7.54700	-67.23660	7.55650
C	-6.47570	-68.21540	7.28860
C	-5.10060	-67.54080	7.25620
C	-4.89720	-66.75550	5.95020
C	-3.74470	-65.73820	5.97760
C	-2.50260	-61.10010	6.40460
C	-3.05080	-60.05430	7.37240
N	-3.31110	-62.29370	6.54730
O	-3.28750	-58.89970	7.02050
O	-2.37440	-61.82000	4.10500
C	-2.52920	-60.66640	4.93690
C	-4.11760	-59.94400	9.64590
C	-3.51870	-58.70570	10.33030
N	-3.28500	-60.49430	8.60540
O	-3.54150	-58.55660	11.54640
C	-5.72420	-61.32780	11.05530
O	-3.87300	-62.31740	9.80680
C	-4.28560	-61.16190	10.56670
O	-5.61410	-62.60840	4.37280
O	-8.78180	-58.66240	3.90060
O	-13.03610	-61.28710	3.98020
C	-6.47020	-59.28440	5.45640
C	-7.14100	-58.71040	6.70920
S	-7.65390	-59.94670	7.93220
C	-8.28840	-58.83930	9.21810
C	-10.65870	-60.34900	1.88600
C	-11.87410	-59.75030	1.15890
C	-11.82870	-59.97500	-0.35040
N	-12.51670	-59.13740	-1.11240
O	-11.20940	-60.91310	-0.84330
N	-2.97370	-57.79060	9.54220
C	-7.77860	-63.06020	-0.05770
C	-8.94720	-63.72270	-0.80250
O	-10.28760	-65.13850	1.26620
H	-9.39400	-61.70150	4.38570
H	-11.25630	-59.52690	3.79160
H	-10.46530	-63.02920	3.52550
H	-13.19500	-63.77260	4.32750
H	-13.90670	-64.32440	2.34210
H	-12.70220	-65.59260	2.28210
H	-12.42850	-62.77260	1.10010
H	-12.91030	-64.21620	0.23190

H	-7.48690	-61.18680	5.38610
H	-6.80500	-60.22860	2.70540
H	-5.90580	-61.50670	1.31430
H	-5.67610	-64.34580	2.40930
H	-5.32600	-63.98970	0.72680
H	-8.01700	-63.45900	2.05590
H	-7.55600	-64.86000	1.07690
H	-10.71920	-62.60210	-0.54360
H	-0.38050	-62.43590	-2.79710
H	-1.04660	-63.48870	-3.82070
H	-1.33270	-65.06930	-2.08430
H	1.48800	-63.95040	-2.35540
H	1.05570	-65.59890	-1.83880
H	0.70500	-65.06740	-3.49570
H	-1.62320	-62.77240	1.68750
H	-2.06650	-62.51520	-1.19310
H	0.33370	-60.77360	3.33490
H	1.94490	-60.98840	2.62740
H	0.84210	-61.28330	0.37070
H	0.69360	-59.68830	1.10510
H	-0.29530	-63.39550	5.69690
H	0.62770	-62.00670	5.16080
H	-0.03100	-64.89950	2.52610
H	0.84320	-63.16490	1.66880
H	-1.59190	-60.23370	1.89180
H	-1.39850	-60.14260	0.14310
H	-3.49660	-62.18250	2.80390
H	-7.52530	-67.12540	4.46220
H	-8.80350	-67.33310	3.25690
H	-7.73080	-65.91740	3.17870
H	-9.54420	-66.48990	5.53290
H	-10.20190	-65.22140	2.95920
H	-7.65530	-62.65680	6.94300
H	-7.85250	-65.64720	6.85710
H	-11.93800	-63.67560	8.99200
H	-10.68860	-64.33120	10.05750
H	-10.08510	-62.20210	8.77970
H	-10.15880	-63.15320	7.29530
H	-9.93070	-66.11440	10.57020
H	-9.78940	-67.81380	10.20100
H	-11.21620	-67.37630	7.03050
H	-11.45510	-65.27140	7.30950
H	-8.12070	-62.95760	9.39440
H	-8.49060	-64.64960	9.05930
H	-3.79660	-64.98540	7.97600
H	-5.57360	-63.78190	5.87050
H	-8.41740	-67.76130	7.64420
H	-7.40040	-66.94610	8.51890
H	-6.66290	-68.71640	6.33880
H	-6.48590	-68.95600	8.08810
H	-4.32130	-68.29940	7.32900
H	-5.04110	-66.89230	8.12810
H	-5.81020	-66.23060	5.68740
H	-4.69570	-67.47460	5.15620
H	-2.81610	-66.26140	6.20710
H	-3.66290	-65.30880	4.97820
H	-1.46850	-61.29450	6.68600
H	-4.30570	-62.20160	6.40770
H	-1.53510	-62.25910	4.36780
H	-1.72870	-59.95280	4.74270

H	-3.48750	-60.21220	4.69050
H	-5.07510	-59.67320	9.20480
H	-3.17040	-61.49040	8.85000
H	-6.38950	-61.45860	10.20360
H	-5.78970	-62.20580	11.69760
H	-6.02570	-60.44410	11.61770
H	-4.62030	-62.94550	9.76240
H	-3.61690	-61.09560	11.42450
H	-6.22240	-58.47110	4.77400
H	-5.54840	-59.76610	5.77260
H	-8.01050	-58.11660	6.43010
H	-6.42150	-58.04660	7.18880
H	-8.65610	-59.43480	10.05360
H	-7.48640	-58.18730	9.56450
H	-9.10210	-58.23690	8.81440
H	-10.42620	-61.34290	1.51570
H	-9.80880	-59.70640	1.65950
H	-11.91140	-58.68060	1.36620
H	-12.80010	-60.20190	1.51050
H	-12.50580	-59.27270	-2.11100
H	-13.03260	-58.37590	-0.69940
H	-2.56070	-56.96240	9.93390
H	-2.99270	-57.97370	8.53730
H	-6.92650	-63.07670	-0.73730
H	-8.02550	-62.02170	0.16540
H	-8.97250	-63.30130	-1.80720
H	-8.78920	-64.79760	-0.89610

JB_UP001_OPLS3e_WATER_82_6%

C	-8.77970	-60.43850	5.10300
N	-9.56510	-61.41220	5.58200
C	-10.99560	-61.31340	5.89060
C	-11.85270	-60.98700	4.65110
N	-11.54170	-61.63910	3.51980
C	-12.28130	-61.55100	2.25160
C	-11.65300	-62.42250	1.14120
C	-11.52100	-63.93170	1.48180
C	-10.57170	-64.66810	0.53630
C	-7.35920	-60.85640	4.69910
N	-7.23520	-60.85750	3.24210
C	-7.83630	-61.73450	2.42260
C	-7.59350	-61.55050	0.90730
C	-6.30700	-62.28440	0.45390
C	-6.32940	-63.82360	0.58790
N	-9.31430	-64.84410	0.95390
C	-5.88180	-57.54080	1.29370
N	-4.16720	-56.96030	-0.48680
C	-4.42890	-57.05710	0.97210
C	-4.07710	-55.75190	1.70080
O	-6.15060	-57.96760	2.42000
C	-8.22210	-57.76970	0.42100
C	-8.54430	-59.25800	0.65490
N	-6.78980	-57.46590	0.30830
O	-9.69490	-59.57950	0.94770
C	-9.25120	-54.73940	-0.04820
C	-8.77830	-55.72080	-1.13690
N	-7.51450	-54.40740	3.18100
N	-9.47410	-53.30450	2.56750
C	-8.51440	-54.07630	2.29580
N	-8.34010	-54.70490	1.08010

C	-8.97740	-57.22080	-0.81220
N	-7.53540	-60.13340	0.54270
C	-13.79330	-61.83080	2.37450
O	-14.57660	-61.20980	1.65750
C	-15.61080	-64.15810	4.67160
C	-15.57900	-62.98990	3.67290
C	-16.34910	-61.77990	4.24040
N	-14.18500	-62.69980	3.31860
O	-17.57370	-61.74160	4.11910
C	-16.16600	-59.54070	5.31570
C	-15.90920	-58.39900	4.30940
N	-15.62930	-60.81420	4.82720
O	-16.24430	-57.25530	4.61650
C	-15.12550	-59.83370	9.11690
C	-15.63690	-60.30310	7.74650
N	-15.00610	-62.02640	12.08820
N	-14.24920	-59.83830	11.96570
C	-14.78750	-60.84000	11.42490
N	-15.22170	-60.88610	10.11240
C	-15.51610	-59.19950	6.67880
C	-14.74610	-57.79120	2.15050
C	-13.55980	-56.95750	2.68770
N	-15.29350	-58.71220	3.15630
O	-13.30400	-55.86380	2.18200
N	-20.07550	-56.87760	-1.21020
C	-19.12430	-57.73920	-0.53180
C	-18.01120	-56.89580	0.10600
C	-16.97420	-57.76550	0.83110
C	-15.85070	-56.93380	1.47990
C	-11.67340	-56.93300	4.35070
C	-12.02180	-55.64240	5.13020
N	-12.83680	-57.50850	3.67430
O	-13.15970	-55.49470	5.58720
O	-9.25090	-56.72950	4.02020
C	-10.49640	-56.81030	3.35200
C	-11.20870	-53.47740	6.02540
C	-11.98590	-52.41250	5.20930
N	-11.03730	-54.76020	5.33250
O	-12.77440	-51.66280	5.77850
C	-9.22670	-53.62310	7.63230
O	-8.86640	-53.01340	5.39590
C	-9.83360	-52.88320	6.43020
O	-8.56200	-62.65120	2.81780
O	-9.13480	-59.27110	4.93960
O	-12.79750	-60.19870	4.74140
C	-6.29670	-59.92510	5.32030
C	-4.84920	-60.33470	4.99470
S	-3.58180	-59.35490	5.84630
C	-3.71580	-57.79100	4.93760
C	-11.27210	-60.37140	7.08710
C	-10.59090	-60.81780	8.39590
C	-11.16700	-60.07040	9.59590
N	-11.69910	-60.79430	10.57080
O	-11.15220	-58.84240	9.63830
N	-11.80450	-52.32420	3.90080
C	-7.26210	-64.53670	-0.42380
C	-8.25790	-65.52750	0.20620
O	-10.97800	-65.07120	-0.55210
H	-9.17110	-62.34080	5.65150
H	-11.30860	-62.31510	6.18910

H	-10.69930	-62.20140	3.50820
H	-12.19710	-60.51090	1.92790
H	-10.68220	-62.00010	0.88320
H	-12.25830	-62.31530	0.23770
H	-12.49100	-64.42470	1.41090
H	-11.17440	-64.08360	2.50390
H	-7.17260	-61.87090	5.05830
H	-6.74280	-60.06960	2.83880
H	-8.44110	-61.98300	0.37510
H	-5.46150	-61.89900	1.02740
H	-6.08720	-62.03120	-0.58460
H	-6.56460	-64.11170	1.61390
H	-5.31400	-64.19420	0.43310
H	-9.04250	-64.39070	1.82050
H	-4.13500	-55.98310	-0.76210
H	-3.22020	-57.27450	-0.68070
H	-3.78380	-57.83920	1.37590
H	-4.65940	-54.91040	1.32310
H	-4.27090	-55.82230	2.77170
H	-3.02160	-55.50640	1.58230
H	-8.59240	-57.26550	1.31280
H	-6.42940	-57.16050	-0.58740
H	-10.25750	-54.98780	0.29110
H	-9.29470	-53.73060	-0.45970
H	-7.73850	-55.51830	-1.39730
H	-9.34220	-55.50550	-2.04580
H	-7.49540	-55.40080	3.37050
H	-7.63560	-53.94890	4.07500
H	-10.04310	-53.19040	1.73280
H	-7.54390	-55.32320	1.00680
H	-10.04500	-57.41250	-0.68830
H	-8.68260	-57.79930	-1.68930
H	-6.63370	-59.76510	0.27550
H	-16.63740	-64.42040	4.93150
H	-15.14720	-65.05200	4.25390
H	-15.09390	-63.91090	5.59980
H	-16.09340	-63.30200	2.76140
H	-13.47450	-63.16820	3.86110
H	-17.24730	-59.61110	5.45320
H	-14.61700	-60.89560	4.85960
H	-15.69890	-58.97350	9.46430
H	-14.08530	-59.51570	9.04430
H	-16.67890	-60.61610	7.82830
H	-15.07150	-61.18200	7.43340
H	-14.96600	-61.93300	13.09180
H	-15.87730	-62.47460	11.84690
H	-14.18510	-59.06710	11.30690
H	-15.60510	-61.76420	9.79030
H	-15.97720	-58.28770	7.06330
H	-14.46220	-58.95270	6.53660
H	-14.31500	-58.42550	1.37440
H	-15.11750	-59.69210	2.96220
H	-20.79100	-57.45490	-1.64820
H	-20.59790	-56.34390	-0.51920
H	-18.70040	-58.44420	-1.24950
H	-19.64340	-58.32760	0.22740
H	-18.43970	-56.18580	0.81580
H	-17.50930	-56.30360	-0.66150
H	-16.53890	-58.47470	0.12550
H	-17.47960	-58.36210	1.59150

H	-15.39430	-56.30670	0.71210
H	-16.27650	-56.24010	2.20590
H	-11.40110	-57.67200	5.10450
H	-13.11160	-58.42210	4.02380
H	-9.06740	-57.62550	4.37140
H	-10.62070	-55.94810	2.69510
H	-10.46600	-57.69280	2.71260
H	-11.78770	-53.66110	6.93260
H	-10.11980	-54.93990	4.93690
H	-9.07760	-54.68350	7.42480
H	-8.26190	-53.20020	7.91470
H	-9.87700	-53.54880	8.50450
H	-9.26550	-52.80300	4.52740
H	-9.93510	-51.82460	6.68320
H	-6.42640	-59.90690	6.40380
H	-6.47150	-58.90080	4.98790
H	-4.66560	-60.27550	3.92100
H	-4.69920	-61.37970	5.27050
H	-2.98350	-57.07230	5.30620
H	-3.52910	-57.95080	3.87590
H	-4.70670	-57.35090	5.04930
H	-12.35050	-60.33090	7.25490
H	-10.97890	-59.34790	6.85170
H	-10.72050	-61.89120	8.53740
H	-9.51640	-60.63870	8.34730
H	-12.21700	-60.34580	11.32240
H	-11.69990	-61.80290	10.52890
H	-12.32120	-51.63720	3.37750
H	-11.06830	-52.85960	3.43760
H	-6.64450	-65.08350	-1.13840
H	-7.81200	-63.82010	-1.03630
H	-8.64910	-66.20630	-0.55680
H	-7.72730	-66.17440	0.90820

JB_UP001_OPLS3e_WATER_88_34%

C	-9.00730	-60.45580	5.39900
N	-9.60460	-59.86770	4.35150
C	-11.04750	-59.76060	4.12480
C	-11.79350	-61.11100	4.07930
N	-11.08910	-62.19110	3.71610
C	-11.57710	-63.56490	3.80320
C	-11.18470	-64.40750	2.57340
C	-11.80540	-63.90810	1.23400
C	-10.84170	-63.82810	0.04670
C	-7.48560	-60.64890	5.29620
N	-7.00840	-60.78880	3.91500
C	-7.38630	-61.73130	3.03830
C	-6.82280	-61.63410	1.60060
C	-6.10530	-62.93410	1.13620
C	-7.03210	-64.08580	0.68810
N	-9.92810	-64.79880	-0.07100
C	-3.01460	-57.82510	3.31510
N	-0.48160	-58.07300	3.24130
C	-1.64830	-57.67550	4.07250
C	-1.43820	-56.28180	4.68490
O	-4.05280	-57.40280	3.83450
C	-4.17940	-58.82610	1.35100
C	-4.75080	-60.18770	1.77610
N	-3.00060	-58.45510	2.13330
O	-4.06130	-60.97840	2.42320

C	-5.02860	-56.75770	-1.23190
C	-3.70010	-57.38590	-0.76270
N	-7.49550	-56.58410	1.52360
N	-7.26940	-58.32070	0.00530
C	-6.92150	-57.18390	0.42540
N	-5.91420	-56.41670	-0.13030
C	-3.83420	-58.79970	-0.15680
N	-6.01200	-60.42610	1.39020
C	-11.02910	-64.21820	5.07660
O	-11.81000	-64.78750	5.83770
C	-7.47660	-64.60190	6.24090
C	-8.98970	-64.83510	6.37080
C	-9.47040	-64.54680	7.80480
N	-9.70770	-64.12070	5.30490
O	-9.23950	-65.37540	8.68490
C	-10.88650	-63.09960	9.24850
C	-12.03440	-64.07900	9.58240
N	-10.16450	-63.42000	8.01430
O	-12.39410	-64.17870	10.75660
C	-12.73510	-59.85600	7.92680
C	-12.40480	-61.34830	8.05340
N	-14.88560	-58.53720	5.23720
N	-14.22350	-57.38720	7.14240
C	-14.24120	-58.43830	6.45030
N	-13.63160	-59.62320	6.81040
C	-11.39600	-61.64050	9.18400
C	-13.74050	-65.66800	8.71600
C	-13.56980	-67.02150	8.00160
N	-12.59590	-64.76280	8.57200
O	-14.22410	-67.98230	8.40940
N	-15.90520	-65.25210	3.39800
C	-15.73690	-64.34160	4.51590
C	-15.35570	-65.12950	5.77610
C	-15.13790	-64.21130	6.98920
C	-15.05790	-64.93250	8.35700
C	-12.46690	-68.32760	6.21100
C	-11.03090	-68.25750	5.66840
N	-12.70900	-67.11180	6.98100
O	-10.78340	-67.60960	4.64850
O	-13.49850	-69.72520	4.45740
C	-13.55710	-68.47070	5.11820
C	-8.68450	-68.99730	6.01080
C	-8.47710	-69.96280	4.81520
N	-10.09430	-68.87860	6.39220
O	-9.07390	-71.03500	4.74690
C	-7.79060	-68.50810	8.36610
O	-8.47370	-70.70380	7.77500
C	-7.87770	-69.53340	7.22460
O	-8.21620	-62.60120	3.31630
O	-9.60440	-60.91000	6.37300
O	-12.98620	-61.13830	4.38520
C	-6.73040	-59.50930	6.01250
C	-5.19780	-59.65150	5.99640
S	-4.55020	-61.12730	6.82870
C	-2.80080	-60.96880	6.37850
C	-11.31190	-58.92070	2.85380
C	-10.60490	-59.41550	1.56680
C	-11.03150	-58.61450	0.33680
N	-10.08420	-58.15470	-0.46780
O	-12.22070	-58.41460	0.09740

N	-7.59100	-69.62190	3.89220
C	-7.70490	-63.83540	-0.68400
C	-8.83230	-64.82700	-1.04080
O	-10.92470	-62.89450	-0.74870
H	-9.01080	-59.52740	3.61070
H	-11.45580	-59.21310	4.97680
H	-10.10340	-62.08300	3.49880
H	-12.66640	-63.57400	3.87690
H	-11.48780	-65.44490	2.73650
H	-10.09480	-64.45400	2.52050
H	-12.23590	-62.91510	1.36800
H	-12.65970	-64.52550	0.95690
H	-7.25510	-61.58090	5.80680
H	-6.31570	-60.12320	3.60630
H	-7.68880	-61.47020	0.96040
H	-5.46490	-63.28820	1.94640
H	-5.42280	-62.70720	0.31600
H	-7.78070	-64.27770	1.45630
H	-6.44880	-65.00600	0.62270
H	-9.97140	-65.56180	0.58860
H	-0.04780	-57.24190	2.84960
H	0.25010	-58.44110	3.84290
H	-1.71430	-58.39720	4.88790
H	-1.36610	-55.51090	3.91690
H	-2.26230	-56.00830	5.34530
H	-0.52690	-56.24470	5.28220
H	-4.95520	-58.09030	1.55640
H	-2.09550	-58.80120	1.84060
H	-5.54180	-57.41400	-1.93530
H	-4.81750	-55.83560	-1.77440
H	-3.19530	-56.71780	-0.06340
H	-3.03800	-57.45070	-1.62750
H	-7.45450	-55.57610	1.50730
H	-8.45510	-56.85710	1.67000
H	-6.76730	-58.53520	-0.85300
H	-5.72700	-55.52560	0.30990
H	-4.57120	-59.35990	-0.73470
H	-2.89740	-59.33870	-0.30580
H	-6.50280	-59.67710	0.88340
H	-6.92570	-65.31570	6.85430
H	-7.13220	-64.72390	5.21370
H	-7.18970	-63.60980	6.58280
H	-9.17510	-65.89450	6.19490
H	-9.13200	-63.62040	4.63150
H	-10.16700	-63.16920	10.06750
H	-10.23700	-62.74590	7.25940
H	-11.82550	-59.27760	7.76040
H	-13.19480	-59.48570	8.84360
H	-12.02270	-61.71510	7.10100
H	-13.32880	-61.89640	8.24000
H	-14.49610	-59.23650	4.62100
H	-14.94720	-57.66170	4.73990
H	-13.70040	-57.53260	8.00120
H	-13.62740	-60.35210	6.10210
H	-10.53700	-60.97490	9.08620
H	-11.85640	-61.38110	10.13910
H	-13.82460	-65.94800	9.76840
H	-12.24510	-64.62260	7.62660
H	-16.11830	-64.71250	2.56300
H	-16.74600	-65.80480	3.53960

H	-16.66310	-63.78600	4.67440
H	-14.96280	-63.61050	4.27690
H	-14.44400	-65.68850	5.57670
H	-16.13290	-65.85990	6.00650
H	-15.97850	-63.51780	7.03820
H	-14.26020	-63.58620	6.82360
H	-15.23150	-64.18590	9.13350
H	-15.89640	-65.62510	8.44920
H	-12.53640	-69.18940	6.87870
H	-12.24960	-66.27400	6.62060
H	-14.19320	-69.73740	3.78110
H	-13.47050	-67.67050	4.37980
H	-14.55090	-68.37340	5.55840
H	-8.31120	-68.01070	5.73240
H	-10.34770	-69.39990	7.21970
H	-8.77320	-68.23810	8.75480
H	-7.19890	-68.88940	9.19890
H	-7.31310	-67.58780	8.02810
H	-8.47580	-71.39210	7.08980
H	-6.86180	-69.78140	6.90570
H	-7.06860	-59.45260	7.04900
H	-7.00130	-58.55000	5.56750
H	-4.75090	-58.77250	6.46360
H	-4.83360	-59.65670	4.96960
H	-2.22420	-61.79650	6.79240
H	-2.67850	-60.97780	5.29440
H	-2.38450	-60.03790	6.76440
H	-11.03090	-57.88350	3.04070
H	-12.39000	-58.89410	2.68090
H	-10.84750	-60.46060	1.37120
H	-9.52100	-59.36490	1.67310
H	-10.32230	-57.63100	-1.29640
H	-9.08870	-58.31630	-0.27090
H	-7.43110	-70.24600	3.11670
H	-7.08990	-68.74870	3.93950
H	-6.94500	-63.87050	-1.46600
H	-8.11310	-62.82620	-0.73640
H	-9.19620	-64.65580	-2.05730
H	-8.42820	-65.84140	-1.02900

Peptide 2

JB_UP002_MMFF_CHCl3_16_9%

C	30.81110	20.09700	30.83480
N	30.54530	19.77140	29.52350
C	31.15620	18.60500	28.89450
C	32.57680	19.02610	28.45790
N	33.59300	18.35630	29.09000
C	35.01070	18.57700	28.82250
C	35.65990	19.41660	29.93040
C	35.06140	20.80750	30.13190
C	35.52240	21.81750	29.10250
C	30.24480	21.45510	31.31130
N	30.95350	22.57760	30.69050
C	30.82200	22.96480	29.37270
C	31.49300	24.32360	29.04860
C	31.63220	24.56540	27.53720
C	32.62700	23.61850	26.85550
N	34.86880	21.75400	27.89630
C	28.97500	25.90330	33.62670

N	30.82250	26.07860	35.25350
C	29.36320	25.85340	35.11750
C	28.60500	26.92410	35.88880
O	28.07160	25.22460	33.14650
C	29.73680	26.77020	31.44890
C	30.74960	25.69980	30.98150
N	29.72320	26.81210	32.90390
O	31.55560	25.15590	31.73890
C	27.69580	28.80620	30.12410
C	28.95040	29.16350	30.93110
N	29.15550	27.80300	27.00710
N	28.22460	26.33660	28.46960
C	28.41620	27.55100	28.10180
N	27.98700	28.67970	28.70360
C	30.09750	28.14360	30.87250
N	30.67200	25.37750	29.64450
C	35.65370	17.17210	28.73890
O	36.55120	16.79090	29.48790
C	36.85760	14.56250	27.54400
C	35.38180	14.93100	27.62030
C	34.62000	14.14970	28.71260
N	35.10670	16.36300	27.76370
O	33.67670	13.40180	28.45210
C	34.19370	14.06130	31.13930
C	33.27170	15.27290	31.42510
N	35.05270	14.37520	30.00380
O	33.47110	16.40180	30.97140
C	34.68530	11.14840	32.30600
C	35.66820	12.32550	32.33880
N	32.74020	12.05180	35.15880
N	31.98360	12.30400	33.00960
C	32.83910	11.82070	33.84030
N	33.90760	11.05940	33.52840
C	35.03910	13.72740	32.37380
C	31.24050	16.03280	32.66270
C	31.81220	16.81290	33.86850
N	32.21870	15.01610	32.26820
O	31.30450	16.81010	34.98880
N	29.00300	11.79780	32.63130
C	28.06630	12.81100	33.10480
C	27.93990	13.98750	32.13210
C	29.24510	14.71750	31.79940
C	29.90020	15.37710	33.01720
C	33.65080	18.34820	34.59390
C	34.05160	17.61140	35.89170
N	33.00990	17.45600	33.61550
O	34.24800	18.17340	36.96510
O	32.75570	20.42690	33.71270
C	32.83750	19.61200	34.88570
C	34.45790	15.32930	36.81640
C	35.33920	14.16710	36.31210
N	34.31530	16.27090	35.70440
O	35.09670	12.97300	36.46650
C	32.44010	15.78570	38.33480
O	32.18310	14.56300	36.27190
C	33.09710	14.82060	37.35150
O	30.21800	22.28180	28.54790
O	31.44940	19.35520	31.58580
O	32.79250	19.94650	27.66950
C	28.71820	21.56510	31.17440

C	27.92110	20.44700	31.85030
S	28.23710	20.21770	33.63100
C	27.61920	21.80320	34.25260
C	30.31980	18.14400	27.68780
C	30.04760	16.63620	27.71200
C	31.30740	15.81050	27.58360
N	31.14690	14.48860	27.86230
O	32.39550	16.26900	27.25290
N	36.50720	14.55470	35.70880
C	34.09340	23.96410	27.14800
C	35.02560	22.77930	26.88250
O	36.41330	22.62710	29.34740
H	30.35080	20.56940	28.90960
H	31.21810	17.81380	29.64900
H	33.36480	17.64180	29.78620
H	35.14070	19.05510	27.84630
H	36.73560	19.50640	29.73140
H	35.56680	18.87550	30.88220
H	35.38340	21.18930	31.10950
H	33.96780	20.79230	30.16790
H	30.50670	21.53530	32.37280
H	31.34170	23.27830	31.32540
H	32.47630	24.34020	29.53100
H	31.93620	25.60370	27.35230
H	30.65200	24.44430	27.05750
H	32.47430	23.67240	25.77010
H	32.40380	22.58670	27.14030
H	34.06290	21.13010	27.80220
H	31.07260	26.00390	36.24070
H	31.31200	25.30640	34.79160
H	29.12260	24.85590	35.50100
H	28.83620	27.93020	35.52020
H	27.52400	26.78040	35.78320
H	28.84710	26.88790	36.95630
H	28.75270	26.43620	31.11230
H	30.60350	27.02900	33.37350
H	26.97340	29.62260	30.24100
H	27.20180	27.91490	30.52160
H	28.65970	29.32040	31.97700
H	29.33740	30.12570	30.57150
H	29.33020	26.94160	26.49730
H	28.73160	28.48120	26.38460
H	27.56340	26.33770	29.25260
H	28.43620	29.51340	28.33620
H	30.44860	28.05590	29.83890
H	30.95000	28.55660	31.42890
H	29.82250	25.64890	29.13440
H	36.96840	13.50430	27.28260
H	37.36870	15.15850	26.78080
H	37.38310	14.70720	28.49210
H	34.92170	14.65240	26.66370
H	34.16810	16.63560	27.45370
H	33.53700	13.23220	30.86380
H	35.67910	15.17470	30.11570
H	35.26610	10.22270	32.21680
H	34.04280	11.18010	31.42160
H	36.32540	12.25280	31.46360
H	36.31470	12.22230	33.21990
H	32.11500	12.82470	35.39620
H	33.61920	12.18940	35.65840

H	32.19170	11.88210	32.09620
H	34.45300	10.76790	34.33410
H	34.45390	13.84070	33.29130
H	35.85060	14.46260	32.46430
H	31.10300	16.74020	31.83860
H	32.11070	14.07520	32.66890
H	28.95660	10.99130	33.25600
H	29.96700	12.13830	32.76930
H	28.36550	13.15540	34.10000
H	27.07970	12.34710	33.21620
H	27.22220	14.70700	32.54550
H	27.50350	13.62370	31.19280
H	29.02520	15.49400	31.05590
H	29.93910	14.02130	31.31550
H	29.21580	16.13150	33.42520
H	30.06070	14.63690	33.80990
H	34.59300	18.64430	34.11420
H	33.22530	17.61290	32.63020
H	32.28710	19.89310	33.03430
H	33.31630	20.21700	35.66210
H	31.82040	19.38770	35.22170
H	35.01530	15.81210	37.62900
H	33.97500	15.87750	34.83310
H	32.15140	16.72410	37.85210
H	31.51560	15.34790	38.72750
H	33.10220	16.00840	39.17680
H	31.76820	15.42340	36.03360
H	33.23860	13.86960	37.87710
H	28.42930	21.56620	30.11690
H	28.39280	22.53890	31.56180
H	28.13620	19.49130	31.36060
H	26.84910	20.62680	31.71390
H	27.58590	21.76890	35.34490
H	28.29060	22.61200	33.95770
H	26.61040	21.99850	33.88040
H	30.80980	18.40930	26.74300
H	29.34570	18.64980	27.68650
H	29.39130	16.37410	26.87420
H	29.53210	16.36600	28.64080
H	31.98250	13.90160	27.89380
H	30.30080	14.10120	28.25750
H	37.18370	13.84800	35.45220
H	36.79130	15.52690	35.69210
H	34.22100	24.30120	28.18180
H	34.38720	24.80270	26.50500
H	34.81640	22.32220	25.91010
H	36.06590	23.12200	26.88470

JB_UP002_OPLS_2005_WATER_24_6%

C	30.01210	20.62140	31.22520
N	30.58950	20.12310	30.12760
C	31.51390	19.00420	30.06440
C	32.28780	19.13190	28.74830
N	33.59710	18.85730	28.76300
C	34.46820	18.92110	27.58330
C	35.94240	18.84180	28.04470
C	36.41690	20.05580	28.88000
C	36.36140	21.37340	28.10220
C	29.21150	21.93180	31.08150
N	29.52970	22.61550	29.82500

C	30.58710	23.41280	29.62030
C	30.77270	24.01710	28.21320
C	31.62530	23.07240	27.32930
C	33.05610	22.75010	27.80430
N	35.78800	22.41940	28.71160
C	27.76730	23.84980	24.66040
N	27.17960	22.57520	22.64880
C	27.74580	22.46710	23.99290
C	29.16300	21.88220	23.92350
O	28.15220	24.83390	24.02360
C	27.35900	25.12800	26.75330
C	28.68470	25.22120	27.54080
N	27.33080	23.92260	25.91820
O	28.98950	26.26550	28.11830
C	23.65620	25.15680	28.10880
C	24.81020	25.14580	27.09540
N	20.17450	25.84830	27.11230
N	21.11150	26.31990	29.15070
C	21.22380	25.86490	27.99050
N	22.36930	25.32380	27.44840
C	26.19390	25.06520	27.76630
N	29.47910	24.14510	27.54700
C	34.17540	17.78510	26.57950
O	34.45450	17.92800	25.39020
C	32.68890	14.43330	27.23350
C	33.08880	15.55150	26.25660
C	31.87450	15.87760	25.36000
N	33.54400	16.69770	27.04570
O	31.56460	15.11580	24.44360
C	30.13510	17.53920	24.74920
C	30.78510	18.41540	23.66450
N	31.18930	17.00220	25.60980
O	30.45410	18.29210	22.48580
C	27.91690	18.50720	27.81070
C	28.36880	17.64700	26.62370
N	25.98440	21.59900	27.26890
N	28.26480	21.43210	27.45480
C	27.14260	20.88060	27.39050
N	26.93970	19.51940	27.44210
C	29.20060	18.43300	25.59360
C	32.41870	20.24040	23.18800
C	33.29780	19.55700	22.11980
N	31.73880	19.27490	24.05330
O	33.41380	20.06240	21.00210
N	35.87470	25.43540	24.45530
C	35.14930	24.57880	23.53550
C	34.58460	23.36380	24.28400
C	33.81510	22.41930	23.35120
C	33.25920	21.18090	24.08050
C	34.70440	17.57820	21.53520
C	33.97260	17.01430	20.29280
N	33.86580	18.38280	22.42580
O	34.62960	16.49570	19.38850
O	34.41190	15.59740	22.99280
C	35.35970	16.45980	22.37800
C	31.83800	16.75950	19.04690
C	31.85490	17.88200	17.97620
N	32.63710	17.12900	20.21800
O	31.44140	17.66570	16.83790
C	30.31440	15.28440	20.52610

O	29.65410	17.53120	20.00170
C	30.38470	16.42260	19.49490
O	31.38530	23.67870	30.51910
O	30.12730	20.07570	32.32400
O	31.69470	19.45100	27.71480
C	27.70600	21.59180	31.07820
C	26.82750	22.83320	31.28820
S	25.04670	22.51510	31.16120
C	24.42290	24.16070	31.60290
C	30.76000	17.65710	30.21160
C	31.62910	16.47730	30.69890
C	32.03830	16.51820	32.17830
N	31.59970	17.50160	32.95810
O	32.77090	15.64250	32.62970
N	32.36020	19.07470	18.29260
C	33.93970	23.97960	28.09690
C	35.45520	23.69230	28.07080
O	36.80000	21.42420	26.95250
H	30.47530	20.64140	29.26810
H	32.22550	19.13240	30.88180
H	34.02220	18.60100	29.64270
H	34.28900	19.85930	27.05580
H	36.58970	18.74840	27.17030
H	36.10260	17.92770	28.61790
H	37.44910	19.89820	29.19420
H	35.83130	20.13540	29.79640
H	29.44630	22.57020	31.93560
H	28.93330	22.41470	29.02360
H	31.25820	24.99110	28.29840
H	31.69720	23.51330	26.33400
H	31.09860	22.12780	27.18550
H	33.53110	22.15140	27.02750
H	33.02160	22.10970	28.68600
H	35.48710	22.29830	29.66950
H	26.22310	22.90830	22.71150
H	27.65430	23.32930	22.16200
H	27.11790	21.79230	24.57910
H	29.84760	22.53540	23.38080
H	29.57540	21.73530	24.92260
H	29.16140	20.91260	23.42680
H	27.26110	26.02610	26.14010
H	27.07300	23.04010	26.36170
H	23.80280	25.97420	28.81540
H	23.64220	24.23080	28.68530
H	24.76350	26.04560	26.48000
H	24.68530	24.30330	26.41370
H	20.40350	26.06250	26.14660
H	19.39220	26.43830	27.37880
H	21.97210	26.26210	29.69670
H	22.24480	24.71180	26.64420
H	26.28150	25.89910	28.46520
H	26.26480	24.16320	28.37340
H	29.09160	23.30460	27.12610
H	32.37260	13.53830	26.69590
H	33.52700	14.14620	27.86890
H	31.86460	14.73560	27.88110
H	33.90830	15.20460	25.62500
H	33.36460	16.67460	28.03720
H	29.55930	16.74050	24.27660
H	31.49470	17.56920	26.38860

H	28.77130	18.96870	28.30310
H	27.45560	17.86370	28.56100
H	27.51220	17.18310	26.13310
H	28.95600	16.82070	27.02580
H	25.12890	21.07500	27.40440
H	25.93160	22.39250	27.89410
H	28.98640	20.71420	27.44730
H	26.00410	19.15920	27.27580
H	28.51050	18.97340	24.94310
H	29.78140	19.20570	26.09420
H	31.65490	20.82610	22.67310
H	31.97040	19.29340	25.03540
H	36.23910	26.23530	23.94430
H	36.70810	24.95020	24.77380
H	35.81740	24.25940	22.73340
H	34.34030	25.14730	23.07300
H	33.91950	23.70070	25.07970
H	35.39410	22.80980	24.76270
H	34.47710	22.11030	22.54160
H	32.99300	22.96400	22.88470
H	34.08050	20.62700	24.53850
H	32.63290	21.52800	24.90270
H	35.50130	18.22360	21.16030
H	33.75250	18.02650	23.36940
H	33.96450	15.10300	22.31470
H	35.98490	16.90270	23.15460
H	36.02960	15.86360	21.75560
H	32.26340	15.86430	18.58770
H	32.15540	17.56890	20.98900
H	30.77520	15.55880	21.47590
H	29.28180	14.99990	20.73080
H	30.82790	14.39570	20.15780
H	30.00100	17.77360	20.86460
H	29.84740	16.08640	18.60580
H	27.48150	20.88170	31.87610
H	27.43320	21.09070	30.14790
H	27.08520	23.60190	30.55920
H	27.03420	23.26090	32.27010
H	23.33530	24.18650	31.53300
H	24.70650	24.41770	32.62380
H	24.82870	24.91920	30.93340
H	30.32620	17.38530	29.24890
H	29.90230	17.75790	30.87720
H	32.52750	16.38630	30.08870
H	31.07360	15.55070	30.54860
H	31.86520	17.52520	33.93270
H	30.99570	18.23860	32.60460
H	32.39170	19.81530	17.60800
H	32.70380	19.25030	19.23140
H	33.66670	24.39070	29.06950
H	33.73190	24.76860	27.37240
H	35.79890	23.71080	27.03540
H	35.99400	24.49630	28.57350

JB_UP002_OPLS_2005_WATER_32_9%

C	29.98210	20.19680	30.80200
N	30.79900	19.89430	29.79020
C	31.55440	18.65980	29.61500
C	32.85950	19.01140	28.89200
N	33.88800	18.16750	29.02060

C	35.20830	18.30680	28.39680
C	36.23270	18.95620	29.36570
C	35.96550	20.43090	29.73550
C	36.18120	21.39820	28.56940
C	29.53730	21.66290	30.93870
N	29.88490	22.50950	29.78950
C	30.39210	23.74880	29.86380
C	30.47450	24.58850	28.57070
C	31.94340	24.80360	28.13420
C	32.74460	23.54630	27.73960
N	35.09700	21.71890	27.85980
C	29.25170	24.62840	24.17180
N	30.40580	25.42680	22.14470
C	30.41650	24.40560	23.19190
C	31.76550	24.42430	23.92630
O	28.73100	25.74350	24.26140
C	27.80780	23.64740	25.93260
C	28.40400	24.11860	27.27510
N	28.84600	23.58250	24.90030
O	27.66940	24.60750	28.13300
C	26.82610	21.13550	23.82800
C	26.18460	21.83160	25.03850
N	24.85310	19.99550	20.93630
N	26.88300	19.15280	21.57930
C	25.93890	19.95270	21.76860
N	25.84610	20.88110	22.78270
C	27.17970	22.24800	26.14000
N	29.72920	24.01280	27.45020
C	35.66580	16.97320	27.77820
O	36.85810	16.66670	27.74930
C	33.62100	14.23380	26.27460
C	34.96200	14.95170	26.49640
C	35.60630	15.26160	25.12850
N	34.71370	16.18160	27.25990
O	36.41960	14.48810	24.62290
C	35.93060	17.12280	23.47770
C	35.72620	18.61800	23.75630
N	35.27490	16.43030	24.57340
O	34.65270	19.01410	24.22290
C	35.47760	16.79350	19.56020
C	36.07410	17.27190	20.89190
N	36.49040	18.28560	16.34090
N	35.93880	16.08930	16.69370
C	36.10460	17.24580	17.14270
N	35.92200	17.62920	18.45400
C	35.38710	16.63560	22.11500
C	36.70160	20.91360	23.65050
C	35.62830	21.63880	22.80570
N	36.72640	19.45630	23.45760
O	35.23850	22.76220	23.12030
N	41.91820	22.22430	20.21230
C	40.58290	21.66690	20.32770
C	40.05770	21.86260	21.75660
C	38.64600	21.28630	21.93820
C	38.11090	21.48560	23.37330
C	34.08790	21.41800	20.83680
C	32.66740	21.27290	21.43580
N	35.12800	20.97260	21.75960
O	31.69720	21.67730	20.79450
O	35.50400	20.38970	19.10670

C	34.16960	20.49490	19.59710
C	31.25870	20.44770	23.31440
C	31.40260	20.70620	24.82610
N	32.52760	20.69260	22.63650
O	30.55020	21.35090	25.43490
C	31.44150	17.85260	23.33590
O	29.30710	18.92460	23.23850
C	30.66030	19.08540	22.85410
O	30.75550	24.24670	30.93030
O	29.60610	19.35150	31.61430
O	32.92090	20.03860	28.21100
C	28.06900	21.88140	31.37090
C	27.02560	21.43510	30.33250
S	25.30290	21.76710	30.79960
C	25.20330	23.54150	30.42810
C	30.72280	17.52340	28.96450
C	30.38260	17.66800	27.45670
C	29.10980	18.44490	27.10570
N	28.29990	18.84540	28.07690
O	28.84070	18.66870	25.93020
N	32.50640	20.28710	25.43820
C	34.15640	23.90770	27.23440
C	34.99380	22.69870	26.78710
O	37.30130	21.85330	28.33620
H	31.10190	20.64970	29.18860
H	31.84700	18.31980	30.61100
H	33.75360	17.35060	29.60150
H	35.11660	18.95540	27.52370
H	37.23280	18.90150	28.93200
H	36.28330	18.36400	30.27970
H	36.64910	20.72270	30.53350
H	34.96600	20.55760	30.15130
H	30.13790	21.99990	31.78580
H	29.61210	22.18890	28.87140
H	30.05010	25.56830	28.80080
H	32.47370	25.31730	28.93790
H	31.94960	25.50330	27.29720
H	32.22000	22.98810	26.96490
H	32.81760	22.88010	28.59950
H	34.23720	21.22580	28.09660
H	29.54190	25.35720	21.61650
H	30.32160	26.33670	22.58770
H	30.28290	23.43500	22.71430
H	31.94880	25.38240	24.41440
H	31.80990	23.65620	24.69690
H	32.59210	24.23320	23.24110
H	27.02880	24.35760	25.64770
H	29.36910	22.71360	24.83290
H	27.25410	20.18890	24.15360
H	27.63500	21.72880	23.40280
H	25.46780	21.13670	25.47900
H	25.59790	22.69570	24.72350
H	23.95520	20.12680	21.39130
H	24.76740	19.19440	20.31880
H	27.66080	19.20220	22.25880
H	25.21420	21.66210	22.62110
H	26.61090	22.26430	27.07130
H	27.94420	21.48280	26.28300
H	30.25710	23.57100	26.71100
H	33.76310	13.30460	25.72110

H	33.15140	13.97530	27.22390
H	32.91840	14.84830	25.70970
H	35.63210	14.30250	27.06370
H	33.75900	16.49850	27.33980
H	37.00080	16.91110	23.52920
H	34.60170	16.99760	25.07110
H	34.38810	16.83450	19.59160
H	35.75300	15.75290	19.38720
H	36.00150	18.35650	20.94570
H	37.13960	17.03790	20.91540
H	36.93240	18.01460	15.46840
H	37.06210	18.99940	16.78030
H	35.64430	15.41660	17.40330
H	35.80050	18.63930	18.63200
H	34.31570	16.82910	22.06350
H	35.49850	15.55260	22.05060
H	36.46170	21.10030	24.69910
H	37.58080	19.05910	23.09430
H	41.86880	23.23490	20.31220
H	42.25100	22.09170	19.25990
H	40.61070	20.60500	20.07610
H	39.92220	22.15090	19.60580
H	40.04380	22.92540	22.00520
H	40.73370	21.38380	22.46750
H	38.66090	20.22510	21.68740
H	37.97650	21.76010	21.21920
H	38.81220	21.04500	24.08310
H	38.10400	22.55280	23.60210
H	34.25840	22.45860	20.55440
H	35.55990	20.09030	21.53250
H	35.78190	21.26040	18.83680
H	33.52440	20.87400	18.80200
H	33.80810	19.49240	19.83610
H	30.55430	21.20890	22.97750
H	33.36590	20.40160	23.12050
H	31.43390	17.76100	24.42200
H	31.02320	16.93400	22.92350
H	32.48130	17.90330	23.01740
H	29.25770	18.87190	24.19870
H	30.65750	19.07240	21.76310
H	27.92650	22.93660	31.60320
H	27.88550	21.35000	32.30570
H	27.11400	20.36030	30.17630
H	27.21910	21.90360	29.36800
H	25.93550	24.10590	31.00540
H	25.38640	23.72200	29.36880
H	24.21160	23.92370	30.67050
H	29.82930	17.32110	29.55500
H	31.31010	16.60970	29.06320
H	30.24010	16.66760	27.04630
H	31.21660	18.08870	26.89620
H	27.43370	19.30950	27.84990
H	28.52070	18.62640	29.03670
H	32.60400	20.40940	26.44210
H	33.23020	19.76940	24.94240
H	34.69500	24.44620	28.01550
H	34.07440	24.59950	26.39470
H	34.52060	22.22070	25.92890
H	35.97820	23.02800	26.44980

JB_UP002_OPLS_2005_WATER_33_9%

C	30.29540	20.74660	31.21950
N	30.53470	20.00770	30.13110
C	31.45500	18.88730	30.00740
C	32.24740	19.04410	28.70420
N	33.56710	18.82930	28.76470
C	34.47500	18.87580	27.61490
C	35.93480	18.82730	28.12430
C	36.37190	20.06280	28.94650
C	36.33340	21.36100	28.13720
C	29.29750	21.91090	31.06640
N	29.55760	22.59850	29.80180
C	30.51870	23.51080	29.61530
C	30.74150	24.04190	28.19160
C	31.63650	23.16150	27.29050
C	33.03230	22.79480	27.82400
N	35.76990	22.42610	28.72080
C	26.55600	27.10800	27.05570
N	26.74750	29.23570	25.77030
C	26.03390	28.54010	26.85200
C	24.52550	28.52890	26.56810
O	26.37320	26.50800	28.11450
C	27.85120	25.27250	25.96260
C	29.18350	25.32340	26.73360
N	27.20620	26.58150	26.01580
O	29.94100	26.28990	26.60810
C	27.95120	22.35180	24.63350
C	28.78470	23.57020	24.19270
N	27.39320	22.08980	27.45860
N	29.11750	20.63510	27.71070
C	28.43540	21.35800	26.95780
N	28.66740	21.53980	25.60930
C	28.11120	24.93090	24.47310
N	29.47410	24.27000	27.50170
C	34.23250	17.69770	26.65000
O	34.43040	17.83340	25.44150
C	32.99390	14.25300	27.38540
C	33.35090	15.37760	26.40130
C	32.14550	15.65060	25.48290
N	33.73620	16.56420	27.16600
O	32.09350	15.14520	24.36280
C	30.01140	16.88010	25.18080
C	30.33760	18.00380	24.17730
N	31.20060	16.48530	25.93650
O	29.67600	18.10430	23.14560
C	27.21370	16.83530	27.95260
C	28.33210	16.29680	27.04690
N	25.20700	14.56440	30.05680
N	24.40240	16.35630	28.87550
C	25.36400	15.63010	29.21260
N	26.67320	15.79100	28.81180
C	28.93890	17.39750	26.16070
C	31.86890	19.84470	23.51890
C	32.63460	19.17210	22.36070
N	31.36670	18.82500	24.44310
O	32.34980	19.44000	21.19310
N	35.52210	25.03730	24.05710
C	34.65690	24.14880	23.30350
C	34.17780	22.99570	24.19650
C	33.23910	22.04150	23.44360

C	32.79590	20.82300	24.28110
C	34.41710	17.55440	21.70500
C	33.68410	16.46740	20.87840
N	33.56650	18.26040	22.67250
O	34.29560	15.85070	20.00350
O	35.32080	16.09160	23.49840
C	35.65110	16.98080	22.44300
C	31.48440	15.42510	20.30270
C	30.26260	16.22630	19.79620
N	32.37550	16.27660	21.09180
O	29.28310	15.65840	19.31540
C	32.40390	13.22850	21.32860
O	30.50320	14.32550	22.28970
C	31.16920	14.10180	21.05660
O	31.20100	23.94160	30.54630
O	30.84440	20.53880	32.30150
O	31.66650	19.29640	27.64300
C	27.86190	21.33790	31.02250
C	27.36790	20.78680	32.37210
S	25.68420	20.10640	32.33950
C	25.96960	18.56520	31.41990
C	30.67600	17.55160	30.03560
C	29.99530	17.20130	31.37730
C	30.97460	16.94640	32.52700
N	30.51690	17.08750	33.76370
O	32.14140	16.62340	32.31310
N	30.30490	17.55670	19.85640
C	33.94290	24.00420	28.11390
C	35.44960	23.68530	28.04910
O	36.77700	21.38040	26.98830
H	30.03450	20.27170	29.28000
H	32.17230	18.91290	30.82950
H	33.96720	18.60880	29.66560
H	34.29730	19.79510	27.05430
H	36.60990	18.72040	27.27270
H	36.08840	17.92890	28.72380
H	37.39390	19.92070	29.29900
H	35.75410	20.15860	29.84000
H	29.39790	22.60580	31.90270
H	29.11580	22.20500	28.97110
H	31.24790	25.00070	28.31850
H	31.75420	23.66070	26.32890
H	31.11330	22.23330	27.06860
H	33.50930	22.16120	27.07620
H	32.94550	22.17270	28.71570
H	35.46450	22.32930	29.68000
H	26.43120	30.19660	25.69370
H	27.73090	29.32580	26.00920
H	26.20900	29.08510	27.78190
H	24.29470	28.00080	25.64190
H	23.98010	28.03250	27.37230
H	24.12760	29.54060	26.48600
H	27.18280	24.53690	26.40970
H	27.31800	27.22320	25.24020
H	27.76670	21.71750	23.76520
H	26.96230	22.64030	24.98800
H	28.95530	23.49750	23.11770
H	29.78220	23.54440	24.63350
H	26.95640	21.66580	28.26810
H	26.65650	22.25660	26.78510

H	29.91110	20.14450	27.28260
H	29.39120	20.96760	25.18630
H	28.73010	25.71170	24.02800
H	27.16500	24.96270	23.93160
H	28.79080	23.51380	27.52710
H	32.71370	13.34320	26.85270
H	33.84220	14.00430	28.02340
H	32.15790	14.52610	28.03090
H	34.19510	15.05720	25.78850
H	33.61430	16.53490	28.16740
H	29.61570	16.02710	24.62520
H	31.33700	16.89470	26.84840
H	26.41850	17.25270	27.33470
H	27.58420	17.64890	28.57780
H	27.93380	15.50350	26.41270
H	29.11450	15.83900	27.65350
H	25.73530	13.73160	29.81780
H	24.24260	14.28200	30.19890
H	24.66790	17.11580	28.24810
H	27.37970	15.30370	29.35720
H	28.12970	17.86640	25.59720
H	29.35560	18.18040	26.79060
H	31.02340	20.40190	23.10950
H	31.85100	18.69690	25.32100
H	34.97500	25.51030	24.76970
H	35.83350	25.79030	23.44890
H	35.20200	23.75860	22.44200
H	33.80600	24.71310	22.91680
H	33.65970	23.39910	25.06460
H	35.03630	22.43740	24.57470
H	33.74840	21.70580	22.53960
H	32.35760	22.59080	23.10970
H	33.67930	20.29980	24.64820
H	32.27360	21.18360	25.16690
H	34.78320	18.28730	20.98300
H	33.74530	18.08840	23.65590
H	35.00000	16.62470	24.22680
H	36.25270	17.79550	22.84880
H	36.29610	16.45710	21.73540
H	31.99330	15.13740	19.38050
H	31.94160	16.80820	21.83120
H	33.10590	13.71340	22.00800
H	32.12250	12.27240	21.77090
H	32.93890	13.01390	20.40290
H	31.14400	14.66820	22.91850
H	30.48990	13.51930	20.43140
H	27.79190	20.56330	30.25810
H	27.17580	22.12590	30.70990
H	27.38810	21.58790	33.11200
H	28.03670	20.01130	32.74650
H	25.04820	17.98610	31.35550
H	26.30670	18.77100	30.40440
H	26.72140	17.95040	31.91500
H	31.34590	16.73500	29.76070
H	29.91570	17.57160	29.25570
H	29.39640	16.29940	31.24710
H	29.29630	17.98970	31.65620
H	31.12780	16.93240	34.55360
H	29.55650	17.35260	33.93200
H	29.52150	18.10490	19.53410

H	31.12000	18.03170	20.22840
H	33.69420	24.41070	29.09510
H	33.73740	24.80390	27.40070
H	35.75720	23.66110	27.00160
H	36.02190	24.49180	28.50910

JB_UP002_OPLS_2005_WATER_41_13%

C	30.96780	20.23030	30.80960
N	30.64760	20.00320	29.52910
C	31.02950	18.83300	28.74770
C	32.49610	19.00210	28.30780
N	33.41300	18.40960	29.07750
C	34.84230	18.31100	28.78320
C	35.64740	19.01430	29.90320
C	35.39130	20.53230	30.04260
C	35.78830	21.34530	28.80740
C	30.57500	21.59460	31.41380
N	30.41610	22.60570	30.36430
C	31.42450	23.27880	29.78830
C	31.08300	24.37630	28.74730
C	31.24820	23.85980	27.29100
C	32.58080	23.16960	26.93530
N	35.01500	22.39150	28.50980
C	26.86930	23.47010	28.97840
N	25.22230	23.88520	27.15330
C	26.02760	22.86630	27.84370
C	26.93380	22.13750	26.84060
O	27.46770	22.74940	29.77500
C	27.64860	25.67970	29.95220
C	29.18850	25.55590	29.88840
N	26.96130	24.80570	28.98670
O	29.90040	26.07250	30.75050
C	25.10600	25.67880	32.97800
C	25.60330	25.82990	31.53230
N	21.71970	26.68160	34.03180
N	23.57910	26.58700	35.37010
C	23.05100	26.44690	34.24450
N	23.70080	26.03930	33.10000
C	27.09860	25.49510	31.39040
N	29.70140	24.83530	28.88280
C	35.27680	16.83790	28.65190
O	36.45030	16.56590	28.39600
C	33.42780	13.68690	29.39320
C	34.63460	14.44210	28.81340
C	34.89790	13.94480	27.38600
N	34.35440	15.87700	28.81500
O	35.87470	13.23210	27.15530
C	34.26470	14.06560	24.98270
C	35.57230	14.65380	24.40430
N	34.08970	14.38320	26.40780
O	36.12120	14.13850	23.43150
C	31.62900	16.48210	23.23370
C	32.91210	16.07790	23.97060
N	31.15500	19.81850	21.73180
N	30.01870	17.90540	21.17650
C	30.88720	18.48350	21.86890
N	31.66360	17.88220	22.83610
C	33.03130	14.55290	24.18090
C	37.25460	16.49490	24.66360
C	38.51330	15.97180	25.39320

N	36.06930	15.72580	25.03220
O	39.59850	16.52450	25.21010
N	33.04220	19.90440	24.44110
C	34.25250	20.62650	24.08410
C	35.47740	20.02990	24.79650
C	35.77380	18.58370	24.37750
C	37.00540	17.96770	25.07310
C	39.46930	14.27130	26.95700
C	39.43840	12.72930	26.90090
N	38.39040	14.90410	26.19560
O	40.14970	12.06580	27.65760
O	38.43000	14.78160	29.13690
C	39.60500	14.92350	28.35240
C	38.53950	10.71710	25.70690
C	38.64480	10.41470	24.19440
N	38.66070	12.15480	25.97180
O	38.30540	9.32630	23.73270
C	37.40600	10.15360	27.97940
O	36.09990	10.73400	26.05890
C	37.31280	10.10790	26.44630
O	32.59990	23.05720	30.08560
O	31.57640	19.40320	31.48900
O	32.78580	19.66000	27.30950
C	29.21910	21.45380	32.14320
C	29.31610	20.80560	33.53440
S	27.72620	20.63650	34.39920
C	27.36080	22.37360	34.78630
C	30.06380	18.73620	27.53550
C	30.36680	17.68900	26.43660
C	30.63630	16.27440	26.95270
N	29.71780	15.34630	26.72550
O	31.69070	16.00350	27.52260
N	39.13890	11.35940	23.39660
C	33.84100	24.04170	27.12470
C	35.12180	23.21190	27.30900
O	36.77040	21.02890	28.13450
H	30.18410	20.75540	29.04220
H	30.92910	17.93390	29.35950
H	33.08640	17.97020	29.92770
H	35.07310	18.77400	27.82320
H	36.71490	18.85930	29.73540
H	35.43510	18.53420	30.85920
H	35.96040	20.91750	30.88900
H	34.34130	20.71020	30.27970
H	31.34470	21.91880	32.11730
H	29.46740	22.83290	30.08320
H	31.74680	25.22530	28.91760
H	31.09860	24.69400	26.60440
H	30.44740	23.15140	27.07120
H	32.54340	22.83260	25.89880
H	32.65440	22.25090	27.51390
H	34.22720	22.58520	29.12470
H	24.68230	23.46650	26.40370
H	24.51300	24.25130	27.78130
H	25.34950	22.13840	28.29360
H	27.63360	22.82150	26.35850
H	27.52180	21.36120	27.33320
H	26.35070	21.64980	26.05920
H	27.43030	26.70260	29.64260
H	26.44470	25.23610	28.22710

H	25.69700	26.31940	33.63370
H	25.24030	24.65360	33.32610
H	25.42830	26.85030	31.18800
H	25.02000	25.18030	30.87860
H	21.48550	27.13290	33.15290
H	21.25870	27.19980	34.77360
H	24.57720	26.36980	35.38400
H	23.11650	25.65430	32.36010
H	27.65370	26.14300	32.07050
H	27.28490	24.47980	31.74040
H	29.02800	24.47980	28.21980
H	33.62290	12.61430	29.43030
H	33.21550	14.01110	30.41200
H	32.52330	13.83320	28.80230
H	35.51330	14.24030	29.42970
H	33.38390	16.15980	28.86960
H	34.32160	12.97880	24.89160
H	33.30360	14.96460	26.68080
H	30.74770	16.29510	23.84710
H	31.52440	15.87550	22.33360
H	32.95840	16.58660	24.93380
H	33.76140	16.43420	23.38750
H	30.88990	20.22190	20.83970
H	32.10600	20.09980	21.94650
H	29.92700	16.91290	21.39630
H	32.21680	18.50120	23.45440
H	32.12520	14.18210	24.66160
H	33.05360	14.07570	23.20000
H	37.42800	16.43630	23.58720
H	35.55360	16.04930	25.83700
H	32.99340	19.86060	25.46440
H	32.24600	20.48620	24.19680
H	34.13700	21.67070	24.37880
H	34.38740	20.61630	23.00140
H	35.33620	20.08530	25.87610
H	36.34700	20.65160	24.57900
H	35.89850	18.53300	23.29520
H	34.89400	17.99070	24.61320
H	37.88190	18.56760	24.82300
H	36.89250	18.04490	26.15520
H	40.40690	14.49530	26.44420
H	37.46120	14.52990	26.34000
H	37.76590	15.37460	28.77640
H	39.84140	15.98400	28.25430
H	40.44070	14.47360	28.89050
H	39.41990	10.21610	26.11480
H	38.10710	12.75780	25.38200
H	37.40560	11.17490	28.36120
H	36.56880	9.62940	28.44110
H	38.32130	9.67360	28.32710
H	36.10370	11.62060	26.42850
H	37.23690	9.05740	26.15950
H	28.50910	20.89830	31.52780
H	28.78830	22.44670	32.26850
H	29.99990	21.37250	34.16750
H	29.74120	19.80550	33.44600
H	26.45450	22.44270	35.38830
H	28.17840	22.82480	35.34900
H	27.20380	22.95380	33.87750
H	30.00680	19.70870	27.04420

H	29.05930	18.54470	27.91480
H	31.23990	18.00370	25.86570
H	29.54710	17.67270	25.71740
H	29.86200	14.39890	27.04380
H	28.85720	15.57710	26.24890
H	39.22680	11.19790	22.40390
H	39.46650	12.22850	23.79010
H	33.73730	24.69230	27.99210
H	33.95190	24.70910	26.26900
H	35.28850	22.58150	26.43410
H	35.98840	23.86850	27.39910

JB_UP002_OPLS_2005_WATER_43_15%

C	31.02130	20.18610	30.79780
N	30.84700	20.12710	29.47340
C	30.80690	18.94770	28.61250
C	32.22350	18.44940	28.23670
N	33.24450	18.74580	29.05250
C	34.66450	18.50320	28.77610
C	35.51740	19.11090	29.91570
C	35.40490	20.64310	30.08590
C	35.81240	21.43440	28.84120
C	30.80880	21.56080	31.47590
N	30.52400	22.58820	30.46350
C	31.45370	23.29150	29.80090
C	30.99770	24.24510	28.66510
C	31.25880	23.64090	27.25400
C	32.65180	23.04140	26.97070
N	35.11040	22.53880	28.58310
C	26.76040	23.42000	30.94480
N	25.17350	24.05670	32.78560
C	25.99300	22.98100	32.20950
C	25.11660	21.75190	31.93130
O	27.39690	22.59990	30.27700
C	27.39850	25.36440	29.51200
C	28.92850	25.24770	29.67140
N	26.70240	24.71990	30.62530
O	29.50520	25.71170	30.65600
C	28.26710	28.82140	30.27640
C	27.16140	27.78630	30.54860
N	29.05690	31.53070	32.64710
N	29.30110	31.61760	30.36920
C	28.96020	30.98730	31.39490
N	28.46800	29.70050	31.41940
C	26.91220	26.82770	29.36060
N	29.56900	24.56600	28.71560
C	35.01000	17.00910	28.62140
O	35.87760	16.65580	27.82190
C	33.93560	14.08360	30.59900
C	34.46650	14.67860	29.28420
C	33.64720	14.06400	28.13110
N	34.31000	16.13240	29.35190
O	33.95080	12.95830	27.68350
C	31.72220	14.34850	26.57310
C	32.33300	14.73410	25.21270
N	32.61800	14.77270	27.64860
O	32.35040	13.92050	24.28900
C	27.95360	15.42200	26.10320
C	29.23300	14.61190	25.84840
N	25.53740	16.83600	25.66250

N	24.69060	15.14230	24.37890
C	25.62960	15.60210	25.06650
N	26.83540	14.96180	25.29060
C	30.37530	15.08420	26.76450
C	33.50770	16.49190	23.89940
C	34.96710	16.00910	23.77970
N	32.86290	15.96000	25.10040
O	35.51850	16.00610	22.67950
N	30.75580	22.21190	23.70270
C	30.70610	20.76170	23.68540
C	32.10840	20.17710	23.90980
C	32.10190	18.64330	23.85760
C	33.51040	18.03380	23.98930
C	36.92260	15.02550	25.07190
C	38.04020	16.08110	25.21590
N	35.58300	15.60470	24.89970
O	39.22700	15.74890	25.22480
O	37.49080	14.16960	22.79840
C	37.20030	13.81280	24.14120
C	38.52740	18.52310	25.45980
C	39.04530	18.71800	26.90510
N	37.65960	17.35660	25.34550
O	39.98100	19.48220	27.13530
C	37.38950	19.69860	23.47380
O	36.43510	19.81900	25.65040
C	37.68680	19.74000	24.98120
O	32.65120	23.17620	30.06670
O	31.36020	19.20040	31.45000
O	32.39770	17.79150	27.21130
C	29.58500	21.47500	32.41750
C	29.87840	20.84350	33.78930
S	28.46670	20.86310	34.93220
C	27.44690	19.54330	34.21430
C	30.05690	19.35820	27.31660
C	28.68990	20.05630	27.52140
C	27.69190	19.23530	28.33950
N	27.03740	19.85280	29.31400
O	27.51060	18.04370	28.10280
N	38.48980	18.01200	27.88740
C	33.82920	24.02850	27.12590
C	35.17410	23.32020	27.35340
O	36.71650	21.03200	28.10780
H	30.63020	21.00780	29.03450
H	30.27920	18.13650	29.11760
H	33.00270	19.21930	29.91200
H	34.90820	18.98060	27.82600
H	36.56680	18.86180	29.75230
H	35.25580	18.63450	30.86160
H	36.04430	20.96390	30.90870
H	34.38720	20.91000	30.37340
H	31.70080	21.82920	32.04550
H	29.55160	22.75100	30.22700
H	31.55740	25.17560	28.77270
H	31.06120	24.41030	26.50620
H	30.52680	22.85440	27.06270
H	32.67280	22.64940	25.95430
H	32.78410	22.16100	27.59920
H	34.37400	22.79130	29.23900
H	25.74570	24.83840	33.08680
H	24.52270	24.43080	32.10300

H	26.74340	22.69880	32.94990
H	24.36310	21.95660	31.16980
H	25.71580	20.91160	31.58160
H	24.60180	21.42120	32.83370
H	27.10580	24.83210	28.60430
H	26.16180	25.30320	31.24410
H	27.99510	29.41970	29.40610
H	29.21270	28.33170	30.04220
H	26.23430	28.32440	30.75280
H	27.37760	27.22790	31.46000
H	28.29640	31.31200	33.28340
H	29.18730	32.53760	32.66140
H	29.18280	31.08760	29.50390
H	28.55670	29.19980	32.30180
H	25.83920	26.80070	29.16620
H	27.34150	27.25130	28.45140
H	29.00210	24.21390	27.95750
H	34.04410	12.99810	30.61070
H	34.48840	14.47010	31.45550
H	32.87950	14.31000	30.75350
H	35.51850	14.41330	29.15960
H	33.62430	16.50700	29.99180
H	31.56400	13.26870	26.61670
H	32.48450	15.70030	28.02500
H	28.14840	16.47270	25.88300
H	27.66700	15.36300	27.15430
H	29.52170	14.71530	24.80160
H	29.04820	13.55010	26.01830
H	26.14570	16.99540	26.46180
H	24.59900	17.14660	25.88940
H	24.85130	14.22410	23.95710
H	26.90140	13.98140	25.02240
H	30.50770	16.15640	26.62660
H	30.05080	14.95800	27.79830
H	32.96500	16.17500	23.00640
H	32.76320	16.59330	25.89150
H	31.39850	22.54020	22.98800
H	31.15350	22.53290	24.57840
H	30.30700	20.42890	22.72550
H	30.01740	20.41160	24.45580
H	32.50330	20.49590	24.87570
H	32.79370	20.55560	23.14930
H	31.65890	18.31700	22.91580
H	31.45360	18.26080	24.64660
H	34.13530	18.44190	23.19250
H	33.97250	18.35870	24.92370
H	36.86870	14.59270	26.07220
H	35.04240	15.69300	25.74860
H	36.79690	14.77770	22.52760
H	36.34860	13.13120	24.15600
H	38.04820	13.24580	24.52850
H	39.39670	18.40550	24.80920
H	36.67080	17.56490	25.36590
H	36.79550	18.82670	23.19760
H	36.84100	20.58580	23.15610
H	38.31350	19.66310	22.89610
H	36.59800	20.16760	26.53160
H	38.23820	20.65920	25.19020
H	28.76750	20.94480	31.92750
H	29.21810	22.48450	32.60530

H	30.69310	21.38980	34.26600
H	30.22090	19.81400	33.68600
H	26.53760	19.40730	34.80000
H	27.15770	19.78310	33.19200
H	27.99100	18.59860	34.20470
H	29.90180	18.47550	26.69460
H	30.68960	20.02200	26.72560
H	28.24010	20.24850	26.54680
H	28.82640	21.03690	27.97830
H	26.36490	19.34050	29.86470
H	27.19150	20.83900	29.51430
H	38.82680	18.09690	28.83360
H	37.70180	17.40490	27.68010
H	33.65950	24.70600	27.96230
H	33.89010	24.66440	26.24160
H	35.40560	22.68100	26.49870
H	35.98040	24.05060	27.43390

JB_UP002_OPLS3e_WATER_2_69_18%

C	29.96150	20.74850	31.22550
N	30.78040	20.27880	30.28520
C	31.93100	19.40000	30.41490
C	32.60050	19.38000	29.03250
N	33.79530	18.78600	28.94170
C	34.53640	18.65280	27.68080
C	36.05930	18.55750	27.93150
C	36.67270	19.78130	28.66940
C	36.44770	21.12980	27.97590
C	28.80550	21.61080	30.69650
N	29.21820	22.42420	29.54680
C	30.06460	23.46370	29.60210
C	30.36640	24.18130	28.27380
C	31.59310	23.59230	27.53460
C	32.91630	23.56750	28.33350
N	35.81360	22.08290	28.67010
C	24.88950	25.20900	28.10610
N	24.51930	27.63020	28.81460
C	23.90440	26.28500	28.67350
C	22.58850	26.32810	27.88320
O	24.71990	24.02230	28.39590
C	26.90880	24.77750	26.70400
C	28.21360	25.08840	27.45960
N	25.84730	25.61870	27.26340
O	28.33610	26.16490	28.05250
C	25.07260	23.74600	24.13930
C	25.76330	25.10590	24.34960
N	23.22510	21.94080	26.78570
N	23.37510	21.32720	24.55440
C	23.67070	22.10850	25.49480
N	24.45800	23.23520	25.35170
C	27.06250	25.08050	25.19300
N	29.17010	24.15400	27.42720
C	34.03780	17.48620	26.80320
O	34.36660	17.44340	25.61690
C	32.24650	14.35450	27.68400
C	32.58890	15.46430	26.67330
C	31.34850	15.96200	25.90940
N	33.24400	16.56940	27.37490
O	30.21460	15.67220	26.30240
C	30.53910	17.35100	24.00460

C	29.60510	16.39990	23.23440
N	31.57750	16.70890	24.82190
O	28.64270	16.87500	22.63320
C	31.12880	20.18200	25.01850
C	31.87860	19.58480	23.81370
N	27.65860	21.42650	24.89710
N	29.02300	21.34150	26.77040
C	28.84040	21.14750	25.53920
N	29.79620	20.64730	24.67530
C	31.15370	18.43070	23.08520
C	28.81620	14.06010	22.98380
C	27.50990	14.18420	23.80360
N	29.80850	15.08110	23.34280
O	26.46460	13.72380	23.34590
N	31.54180	12.04730	18.63490
C	31.78340	12.01480	20.06580
C	30.51110	12.41900	20.82430
C	30.71590	12.38590	22.34770
C	29.43050	12.65270	23.15980
C	26.45340	15.13710	25.85100
C	25.58690	16.33590	25.39190
N	27.58600	14.82130	24.98090
O	24.53170	16.54720	25.99400
O	27.88590	16.35210	27.47010
C	26.97670	15.27340	27.29870
C	25.26200	18.22560	23.78600
C	23.83830	17.85260	23.28890
N	26.00730	17.08720	24.35870
O	23.63350	16.81110	22.66870
C	25.44300	20.12900	21.97620
O	27.35850	19.17200	22.97410
C	26.03580	18.84640	22.58450
O	30.56240	23.88530	30.64680
O	30.07580	20.52500	32.43040
O	32.02080	19.89150	28.07140
C	27.63320	20.69310	30.28620
C	26.41260	21.42470	29.70510
S	25.04970	20.31880	29.24540
C	25.73390	19.57340	27.73740
C	31.49160	17.99460	30.90330
C	30.42910	17.28490	30.02830
C	29.95710	15.95960	30.62610
N	28.88730	15.39780	30.08390
O	30.55210	15.42770	31.56110
N	22.85230	18.70650	23.51740
C	34.15500	23.49350	27.41560
C	35.50520	23.42170	28.15870
O	36.85020	21.30130	26.82640
H	30.61690	20.55540	29.32240
H	32.62680	19.84370	31.13030
H	34.22690	18.39960	29.76930
H	34.35370	19.54810	27.08170
H	36.58330	18.40840	26.98410
H	36.27560	17.65350	28.50360
H	37.75140	19.64500	28.74890
H	36.32860	19.81080	29.70380
H	28.48160	22.27750	31.49840
H	28.90780	22.10900	28.62680
H	30.60710	25.21760	28.52200
H	31.73510	24.17380	26.62250

H	31.37180	22.57810	27.20420
H	32.91230	22.71770	29.01920
H	32.98950	24.46060	28.95710
H	35.53630	21.87660	29.61930
H	24.49900	28.16340	27.95050
H	25.49230	27.58450	29.10710
H	23.64440	25.93290	29.67350
H	22.74190	26.68200	26.86310
H	22.12550	25.34200	27.82180
H	21.86740	26.99230	28.36000
H	26.68730	23.71700	26.84360
H	25.94740	26.61120	27.11330
H	25.77640	23.01640	23.74280
H	24.28320	23.85410	23.39490
H	25.05070	25.82620	24.75310
H	26.02270	25.49590	23.36420
H	23.12550	20.97330	27.05380
H	23.79140	22.41030	27.48160
H	23.77250	21.67150	23.68690
H	24.63750	23.77120	26.19220
H	27.75990	24.36440	24.75530
H	27.54580	26.05280	25.07980
H	28.99130	23.25590	26.97230
H	31.77420	13.50710	27.18520
H	33.14140	13.97870	28.18010
H	31.55660	14.70010	28.45360
H	33.28320	15.04340	25.94260
H	33.00610	16.69000	28.34690
H	29.86260	17.84800	24.70260
H	32.54070	16.94490	24.60820
H	31.69780	21.01300	25.43530
H	31.03580	19.44000	25.80950
H	32.85560	19.23880	24.15270
H	32.08870	20.37820	23.09500
H	27.43350	20.75270	24.17230
H	26.87140	21.48840	25.52350
H	29.95130	20.99750	27.00790
H	29.50610	20.50440	23.71720
H	30.36400	18.86170	22.46700
H	31.84440	17.96610	22.38000
H	28.54450	14.20660	21.93690
H	30.61690	14.76770	23.85900
H	32.38580	11.74830	18.14980
H	31.42990	13.01260	18.33410
H	32.60720	12.68790	20.31150
H	32.09330	11.00910	20.35560
H	29.69300	11.74750	20.55730
H	30.20520	13.42100	20.52000
H	31.49910	13.09080	22.62860
H	31.09520	11.40160	22.62660
H	28.68390	11.90060	22.89660
H	29.64450	12.48440	24.21710
H	25.78410	14.27380	25.84040
H	28.49320	15.14280	25.29930
H	28.74900	16.10420	27.07960
H	27.46210	14.34640	27.60880
H	26.13790	15.41770	27.98200
H	25.16120	18.97520	24.57290
H	26.86750	16.84280	23.88870
H	25.36470	20.91850	22.72080

H	26.06890	20.50500	21.16620
H	24.45100	19.96590	21.55490
H	27.89720	18.36360	22.85120
H	26.10490	18.09870	21.79040
H	27.31690	20.10910	31.15220
H	27.98800	19.96400	29.55600
H	26.69100	22.00280	28.82270
H	26.03650	22.14540	30.43250
H	24.99030	18.93550	27.25990
H	26.02630	20.34410	27.02490
H	26.60950	18.96180	27.95650
H	31.10940	18.07980	31.92150
H	32.36860	17.35170	30.99150
H	30.81230	17.09720	29.02550
H	29.55520	17.92570	29.90720
H	28.52960	14.52250	30.43800
H	28.42190	15.83580	29.29080
H	21.92330	18.48910	23.19790
H	23.02690	19.60420	23.98320
H	34.16520	24.38040	26.77990
H	34.06650	22.64810	26.73040
H	36.30490	23.80100	27.51570
H	35.49060	24.11420	29.00320

JB_UP002_OPLS3e_WATER_2_71_2%

C	31.99630	19.70910	30.60310
N	31.00390	20.14140	29.82090
C	30.66000	19.59620	28.51590
C	31.42670	20.39160	27.45100
N	32.45990	19.77050	26.86910
C	33.28810	20.34610	25.80560
C	34.53500	19.47780	25.51380
C	35.57630	19.43920	26.66560
C	36.15430	20.81230	27.01680
C	32.28140	20.52900	31.87360
N	32.60490	21.93490	31.58310
C	31.80160	22.89160	31.09320
C	32.46390	24.25050	30.81150
C	32.89710	24.37670	29.33220
C	33.88970	23.32730	28.78520
N	35.98460	21.24830	28.26920
C	30.01230	28.94030	31.37660
N	28.40110	30.17880	29.92420
C	29.76260	29.59610	29.97950
C	30.82790	30.64240	29.61590
O	30.16800	29.65610	32.37400
C	30.20760	26.80090	32.63980
C	31.25840	25.69460	32.43530
N	29.99370	27.60170	31.42940
O	31.79550	25.19500	33.42540
C	26.75910	24.83970	32.69910
C	28.04540	25.44100	32.12150
N	24.49720	22.72840	30.67920
N	24.23070	23.27330	32.91890
C	24.88150	23.35350	31.84470
N	26.05200	24.07160	31.69090
C	28.85900	26.24170	33.15710
N	31.55770	25.33920	31.17830
C	32.52530	20.67130	24.50870
O	32.97750	21.54540	23.76640

C	29.32550	19.30720	23.09840
C	30.45020	20.35080	23.18000
C	29.86680	21.77640	23.24300
N	31.36600	20.03070	24.28110
O	29.42530	22.27740	22.20840
C	29.63730	23.87610	24.57730
C	30.77910	24.76750	24.03180
N	29.93680	22.44820	24.40410
O	31.23420	25.68050	24.72330
C	27.92260	23.62110	28.14500
C	28.17330	23.33490	26.65610
N	27.56930	27.25100	28.71490
N	29.47240	25.98120	29.13020
C	28.26960	26.06740	28.76230
N	27.49790	24.99460	28.35890
C	29.33090	24.16690	26.06690
C	32.33730	25.11900	22.06460
C	33.64950	25.27260	22.86730
N	31.25970	24.45880	22.81720
O	34.34270	26.28140	22.71860
N	28.58890	28.82250	18.48910
C	28.94470	27.53530	19.05800
C	30.17650	27.68100	19.96280
C	30.59880	26.33970	20.57950
C	31.83640	26.46860	21.48900
C	35.03460	24.31950	24.74220
C	35.01340	25.55970	25.68600
N	33.93470	24.31300	23.76390
O	36.03040	25.89080	26.29830
O	37.46190	23.71220	24.79890
C	36.37040	24.09530	23.97750
C	33.68320	27.52970	26.45030
C	32.58140	27.48220	27.53450
N	33.86100	26.23650	25.78300
O	32.77110	28.01520	28.62290
C	33.16230	30.04500	25.95490
O	34.28220	28.65750	24.37180
C	33.30240	28.61470	25.40240
O	30.60780	22.72760	30.82930
O	32.69030	18.72570	30.34650
O	31.06640	21.53330	27.16350
C	31.13990	20.39030	32.91060
C	31.39890	21.13290	34.23430
S	30.00210	21.11010	35.39070
C	28.89430	22.28800	34.56610
C	29.12070	19.65640	28.33770
C	28.54450	19.20610	26.97420
C	29.08500	17.86460	26.48200
N	28.34140	16.78580	26.68050
O	30.17730	17.80310	25.92200
N	31.46450	26.81610	27.28090
C	35.23850	23.26820	29.53890
C	36.37970	22.56800	28.77450
O	36.71820	21.47650	26.14910
H	30.58120	21.03450	30.06960
H	30.97010	18.54940	28.47510
H	32.67850	18.82550	27.15130
H	33.61910	21.32090	26.16790
H	35.02850	19.82160	24.60070
H	34.21870	18.45970	25.27980

H	36.41810	18.81390	26.36740
H	35.16000	18.94360	27.54350
H	33.18120	20.10050	32.31930
H	33.57230	22.17780	31.74030
H	33.35150	24.34500	31.44020
H	33.34270	25.36040	29.19190
H	32.00620	24.36450	28.70120
H	34.07900	23.56690	27.73850
H	33.42530	22.34000	28.76460
H	35.57740	20.60920	28.93680
H	28.37780	31.05530	30.43620
H	27.73160	29.58090	30.39930
H	29.83940	28.80750	29.22930
H	30.79470	31.50370	30.28390
H	31.83350	30.22600	29.67350
H	30.68650	31.00530	28.59780
H	30.61870	27.43940	33.42470
H	29.81280	27.07870	30.56630
H	26.10200	25.62760	33.06890
H	26.98510	24.18610	33.54110
H	27.78010	26.09270	31.28990
H	28.66360	24.64460	31.70360
H	23.53200	22.43510	30.68490
H	24.65630	23.27910	29.84890
H	24.68670	23.80670	33.65380
H	26.49790	24.03230	30.78370
H	28.25160	27.06800	33.52790
H	29.05060	25.61020	34.02570
H	31.01720	25.74680	30.42030
H	28.73380	19.44920	22.19300
H	29.72110	18.29180	23.06900
H	28.63950	19.37990	23.94090
H	31.02990	20.28690	22.25660
H	31.06730	19.30560	24.92810
H	28.74520	24.12020	23.99660
H	30.34030	21.98980	25.21490
H	28.81570	23.40550	28.73250
H	27.14020	22.96140	28.52080
H	27.26160	23.51370	26.08500
H	28.39040	22.27320	26.54890
H	26.97090	27.33880	27.90770
H	28.15700	28.07200	28.76290
H	29.74220	25.00080	29.07850
H	26.54510	25.20010	28.09160
H	29.09860	25.22720	26.17970
H	30.22900	24.00230	26.66470
H	32.57870	24.46240	21.22750
H	30.80170	23.68590	22.34210
H	28.22810	29.42690	19.22370
H	27.78760	28.70160	17.87240
H	29.14980	26.82910	18.25120
H	28.09780	27.14030	19.62260
H	29.97050	28.39310	20.76410
H	31.01030	28.09090	19.38970
H	30.80780	25.62550	19.78180
H	29.76120	25.93070	21.14590
H	32.64010	26.93430	20.91560
H	31.61920	27.15710	22.30760
H	34.85860	23.45170	25.37860
H	33.34480	23.48460	23.78210

H	37.22720	22.87060	25.24420
H	36.64810	24.99620	23.42800
H	36.24000	23.30820	23.23290
H	34.60550	27.82950	26.95070
H	33.03300	25.88890	25.30660
H	34.06700	30.36400	26.47350
H	32.96700	30.76110	25.15590
H	32.33350	30.12750	26.65890
H	34.27170	27.80490	23.89640
H	32.35380	28.33790	24.93550
H	30.99040	19.33130	33.12830
H	30.19870	20.72610	32.47620
H	31.66180	22.17570	34.05120
H	32.26070	20.68870	34.73450
H	28.01530	22.47710	35.18260
H	29.40120	23.23800	34.39550
H	28.55290	21.90790	33.60320
H	28.77530	20.67320	28.52840
H	28.66300	19.04120	29.11320
H	28.78190	19.95460	26.21900
H	27.45530	19.17630	27.02210
H	28.66130	15.87920	26.36890
H	27.44490	16.84840	27.14330
H	30.75760	26.70210	28.01060
H	31.30590	26.38990	26.37270
H	35.10360	22.77850	30.50300
H	35.56710	24.28270	29.77140
H	36.68460	23.22210	27.95180
H	37.25470	22.45490	29.41860

6S35_X-ray_76_16%

N	25.55480	22.50950	22.29070
C	26.94200	22.92770	22.47050
C	27.04230	23.82560	23.66790
O	26.92500	25.04730	23.64960
C	27.40310	23.66820	21.25030
N	27.27890	23.13740	24.78520
C	27.43050	23.71200	26.11880
C	28.78950	23.37490	26.65690
O	29.42990	22.36440	26.38230
C	26.37890	23.14910	27.02830
C	25.01990	23.48620	26.49010
C	23.96830	22.92330	27.39960
N	24.11990	23.49780	28.73320
C	23.41600	24.72840	29.08220
N	22.54630	25.43400	28.14550
N	23.57190	25.22250	30.29230
N	29.49340	22.38590	29.32070
C	29.09610	21.49220	30.41300
C	30.09300	20.36350	30.70010
O	29.68570	19.22170	30.89780
C	27.76820	20.88400	30.07110
C	26.76340	21.25420	31.12150
S	25.18770	20.53250	30.71570
C	24.42360	21.22710	32.16560
N	31.39040	20.67550	30.69820
C	32.48210	19.71590	30.78720
C	32.95770	19.27660	29.39890
O	32.43280	19.71930	28.37540
C	32.07600	18.51060	31.58250

C	32.97620	18.37140	32.77420
C	32.86410	19.59710	33.63140
O	31.83240	20.00010	34.16060
N	34.03580	20.22000	33.76150
N	34.00000	18.43710	29.37130
C	34.67040	17.95900	28.16680
C	34.75680	16.46410	28.25520
O	34.33250	15.78340	29.18440
C	36.08850	18.54870	28.01080
C	36.14260	20.08040	27.80680
C	36.01240	20.89520	29.10500
O	36.55170	20.49700	30.13250
N	35.35960	15.93830	27.18850
C	35.60130	14.51570	26.96660
C	36.80790	14.08580	27.74740
O	36.86520	14.18370	28.97110
C	35.83770	14.27050	25.50580
N	29.24080	24.31490	27.48790
C	30.52670	24.28920	28.17900
C	30.39060	23.37490	29.39780
O	31.06720	23.56880	30.40440
C	31.62840	23.86820	27.18030
C	32.99960	24.55310	27.37690
C	33.90160	23.93620	28.45820
C	34.51950	22.59520	28.03610
N	35.30410	22.03010	29.12370
H	25.24430	22.01090	23.11230
H	24.97320	23.32340	22.15090
H	25.48720	21.90500	21.48440
H	27.56960	22.04940	22.62130
H	28.43880	23.98050	21.38450
H	27.33020	23.01580	20.38010
H	26.77550	24.54650	21.09940
H	27.38220	22.13450	24.84560
H	27.32040	24.79500	26.06250
H	26.49210	23.57810	28.02390
H	26.48900	22.06610	27.08460
H	24.90670	23.05720	25.49450
H	24.90980	24.56910	26.43380
H	24.07840	21.84030	27.45590
H	22.98070	23.16820	27.00860
H	24.71160	23.04390	29.41440
H	22.07940	26.28280	28.43120
H	22.41170	25.07330	27.21170
H	23.10110	26.07700	30.57840
H	24.16790	24.76440	30.97690
H	29.03840	22.23410	28.43420
H	28.94840	22.06460	31.33030
H	27.86570	19.79920	30.02780
H	27.43590	21.25780	29.10260
H	26.66590	22.33900	31.16470
H	27.09570	20.88040	32.08990
H	23.37330	20.93740	32.19690
H	24.92970	20.85520	33.05650
H	24.49990	22.31380	32.13130
H	31.61760	21.64430	30.50170
H	33.31130	20.18450	31.31700
H	32.15740	17.61990	30.95950
H	31.04520	18.62680	31.91740
H	34.00700	18.25520	32.43930

H	32.68110	17.49560	33.35210
H	34.19560	21.06400	34.29290
H	34.90690	19.92410	33.34470
H	34.40030	18.16520	30.25690
H	34.07960	18.16870	27.27130
H	36.55340	18.07990	27.14340
H	36.70930	18.25590	28.85950
H	35.39250	20.36200	27.06880
H	37.10710	20.34320	27.37400
H	35.72270	16.45990	26.40350
H	34.73340	13.94410	27.29520
H	37.63040	13.68090	27.15780
H	36.01810	13.20840	25.34020
H	34.96090	14.58290	24.93850
H	36.70560	14.84210	25.17720
H	28.74830	25.15960	27.74100
H	30.69070	25.31070	28.52380
H	31.27360	24.13750	26.18490
H	31.73240	22.78250	27.15620
H	32.84540	25.61090	27.59550
H	33.54050	24.53310	26.42970
H	33.34920	23.82350	29.38910
H	34.71000	24.63580	28.67750
H	35.15260	22.73760	27.15790
H	33.73550	21.88880	27.76160
H	35.27180	22.50530	30.01230

Peptide 1 MD analysis (macrocycle)

Peptide 1 cluster1

N	1.15200	0.37800	-4.72100
C	0.81500	1.18800	-3.53500
C	-0.57500	0.74800	-2.91700
C	-0.69100	-0.69600	-2.33600
C	-2.12600	-1.25900	-2.20300
C	-2.12700	-2.67400	-1.50800
N	-1.59600	-2.61400	-0.17900
C	-2.16300	-2.81200	0.94800
C	-1.17500	-2.63300	2.10300
C	-1.73400	-1.85000	3.20400
C	-1.93100	-0.29000	2.88300
N	-0.58000	0.25900	2.85500
C	-0.20700	1.27700	2.06700
C	1.19000	1.86600	2.19500
N	1.73800	2.06900	0.85100
C	3.02500	1.74100	0.51200
C	3.46500	2.26000	-0.87600
N	2.29500	2.08800	-1.77900
C	2.06800	1.03300	-2.57500
O	-3.37800	-3.14600	1.09100
C	-2.73800	0.31100	4.03800
O	-3.96500	0.42400	3.93000
N	-1.99700	0.76500	5.04900
O	-0.93300	1.77100	1.18200
C	1.32200	3.07900	3.10400
O	3.76700	0.90600	1.04900
C	3.62800	3.82400	-0.82700
O	2.83400	0.08900	-2.71000
H	1.94100	-0.19900	-4.48100

H	0.62000	2.20300	-3.87300
H	-0.79500	1.34900	-2.04000
H	-1.37300	1.03600	-3.60100
H	-0.23400	-0.77700	-1.35200
H	-0.09000	-1.48900	-2.80100
H	-2.56900	-1.46300	-3.17000
H	-2.68000	-0.60400	-1.53100
H	-1.45800	-3.37400	-2.00800
H	-3.14800	-3.06300	-1.44700
H	-0.59900	-2.45600	-0.09800
H	-0.25400	-2.17800	1.74900
H	-0.93700	-3.66800	2.36600
H	-1.16800	-1.99900	4.11300
H	-2.72000	-2.25400	3.44500
H	-2.41300	0.00800	1.94600
H	0.10800	-0.03400	3.52600
H	1.83300	1.12100	2.66400
H	1.13000	2.40600	0.11400
H	4.30600	1.69000	-1.28200
H	1.65100	2.86200	-1.79200
H	-0.98800	0.74300	4.96100
H	0.87300	2.93200	4.07600
H	2.38700	3.07100	3.31900
H	3.76200	4.17000	-1.84800
H	2.72000	4.26600	-0.41300

Peptide 1 cluster2

N	0.78100	0.52900	-4.91900
C	0.76100	1.31500	-3.68200
C	-0.55600	0.99100	-2.89400
C	-0.49800	-0.28400	-2.01500
C	-1.86700	-0.91900	-1.67900
C	-1.66800	-2.36500	-1.25200
N	-1.35600	-2.51800	0.09600
C	-2.11700	-2.95800	1.11200
C	-1.56500	-2.91000	2.55000
C	-2.09800	-1.72500	3.35800
C	-2.12900	-0.30800	2.68800
N	-0.76200	0.16000	2.68600
C	-0.30700	1.06700	1.83300
C	1.15300	1.54000	1.98600
N	1.78500	1.83300	0.67300
C	3.06500	1.67700	0.31400
C	3.49500	2.07200	-1.12500
N	2.32900	2.03300	-2.03300
C	2.00400	1.04700	-2.85200
O	-3.28100	-3.29900	1.04100
C	-2.93200	0.66400	3.58900
O	-2.38900	1.59600	4.22900
N	-4.27200	0.52700	3.65400
O	-0.98900	1.43000	0.85200
C	1.11400	2.81700	2.87000
O	3.83200	1.18300	1.11600
C	4.01700	3.53200	-0.97700
O	2.58100	-0.04500	-2.83700
H	-0.06100	-0.01400	-5.05100
H	0.81700	2.37300	-3.95100
H	-0.72600	1.80900	-2.20000
H	-1.37700	0.92700	-3.60100
H	0.01500	-0.02700	-1.09200

H	0.02900	-1.04500	-2.59700
H	-2.42800	-0.86500	-2.61200
H	-2.38700	-0.28200	-0.96000
H	-1.03600	-2.90400	-1.95100
H	-2.63800	-2.87000	-1.28900
H	-0.44100	-2.24800	0.42100
H	-0.47300	-2.85600	2.49300
H	-1.79200	-3.81300	3.11500
H	-1.51900	-1.81000	4.28200
H	-3.11500	-2.05500	3.57100
H	-2.42300	-0.47000	1.65800
H	-0.14900	-0.19200	3.40400
H	1.63400	0.68200	2.44400
H	1.08400	2.17100	0.02300
H	4.30800	1.42600	-1.45600
H	1.78100	2.88800	-2.04500
H	-4.64900	-0.33300	3.28600
H	0.77200	3.62900	2.22800
H	0.36400	2.61100	3.62700
H	4.01800	4.04700	-1.94000
H	3.39600	4.16200	-0.34600

Peptide 1 cluster3

N	1.48400	1.43200	-3.85500
C	0.70600	0.96800	-2.75700
C	-0.21500	-0.15000	-3.23700
C	-1.42200	-0.43000	-2.45900
C	-2.06900	-1.64900	-2.81100
C	-2.95600	-2.33800	-1.73500
N	-2.22800	-2.92700	-0.65400
C	-2.26100	-2.49400	0.59600
C	-1.11500	-2.87100	1.51000
C	-0.18300	-1.73600	1.91000
C	-0.84800	-0.53800	2.59500
N	-0.03100	0.62000	2.35700
C	-0.24500	1.91800	2.53100
C	0.86800	2.94500	2.41900
N	1.65900	2.61500	1.20400
C	2.62100	1.72200	1.20400
C	3.34000	1.55300	-0.07400
N	2.45800	1.71800	-1.24500
C	1.61100	0.73900	-1.62900
O	-3.28800	-2.10800	1.10300
C	-0.92500	-0.77300	4.15300
O	0.06900	-0.56600	4.84000
N	-2.09100	-0.99300	4.65500
O	-1.36500	2.27900	2.62700
C	0.34300	4.39600	2.32200
O	2.80500	0.97700	2.10000
C	4.55300	2.52100	-0.14900
O	1.58400	-0.33600	-1.02100
H	2.17800	0.83000	-4.27200
H	0.12800	1.79500	-2.35000
H	-0.60100	-0.06900	-4.25400
H	0.53600	-0.93600	-3.33900
H	-2.10400	0.41900	-2.54100
H	-1.12000	-0.36400	-1.41500
H	-1.29100	-2.33100	-3.18200
H	-2.70700	-1.45400	-3.67500
H	-3.59200	-3.16500	-2.06500

H	-3.66700	-1.57300	-1.42800
H	-1.38100	-3.41000	-0.90700
H	-0.56000	-3.66300	0.99600
H	-1.55200	-3.42200	2.34700
H	0.12600	-1.44100	0.90300
H	0.70700	-2.11600	2.41300
H	-1.85500	-0.39500	2.19000
H	0.96900	0.48700	2.32400
H	1.51600	2.94500	3.28900
H	1.45100	3.20300	0.41600
H	3.71300	0.52800	-0.04300
H	2.23600	2.57900	-1.72400
H	-2.81100	-1.20300	3.97700
H	-0.07800	4.54300	1.32600
H	-0.44900	4.63000	3.04000
H	5.13500	2.40700	-1.05800
H	4.13800	3.52600	-0.22400

Peptide 1 cluster4

N	2.04900	0.73200	-5.14300
C	1.05100	0.83200	-4.05800
C	0.57400	-0.58800	-3.74000
C	-0.58900	-0.70700	-2.72400
C	-0.90300	-2.19400	-2.71900
C	-2.04600	-2.55100	-1.68900
N	-1.60400	-2.28800	-0.35800
C	-2.19100	-1.53200	0.52900
C	-1.83300	-1.85800	1.96700
C	-2.09800	-0.72200	2.90800
C	-1.12000	-0.53800	4.05400
N	0.22800	-0.25200	3.55900
C	0.59600	0.97900	3.21100
C	2.05800	1.21000	2.68400
N	2.01900	1.92700	1.36300
C	1.34500	1.65400	0.28700
C	1.54400	2.75400	-0.82300
N	0.99300	2.22600	-2.08000
C	1.70400	1.38000	-2.78400
O	-3.23700	-0.94400	0.26000
C	-1.00400	-1.72700	5.03900
O	-0.13700	-2.57200	4.90000
N	-1.87200	-1.82700	6.03400
O	-0.12400	1.98500	3.28200
C	2.91400	2.11200	3.59700
O	0.60200	0.66500	0.26900
C	0.66100	3.93900	-0.37200
O	2.85700	0.98900	-2.47400
H	2.46400	-0.19000	-5.19100
H	0.24000	1.48000	-4.37800
H	0.28200	-1.00200	-4.70400
H	1.38500	-1.20500	-3.35400
H	-1.40100	-0.08100	-3.09900
H	-0.43900	-0.25300	-1.74600
H	-0.00300	-2.81400	-2.69600
H	-1.25400	-2.41300	-3.73200
H	-2.38200	-3.58100	-1.80300
H	-2.98300	-2.03600	-1.93200
H	-0.94800	-2.92800	0.06200
H	-0.81500	-2.24300	2.05000
H	-2.40400	-2.74700	2.25200

H	-3.12400	-0.86500	3.28100
H	-2.08800	0.19900	2.33100
H	-1.44700	0.26100	4.71700
H	0.82700	-1.07000	3.52600
H	2.62300	0.27300	2.64800
H	2.72900	2.63600	1.24200
H	2.57000	3.08900	-0.99300
H	0.11300	2.58000	-2.43500
H	-2.61400	-1.15200	5.88900
H	3.85400	2.29500	3.07500
H	2.42000	3.07500	3.74600
H	-0.37600	3.61800	-0.28500
H	0.95400	4.19200	0.64300

Peptide 1 cluster5

N	1.38000	0.38800	-4.98500
C	0.91600	0.64300	-3.68900
C	-0.33100	-0.19600	-3.41100
C	-1.27000	0.34100	-2.25600
C	-2.52200	-0.52100	-2.03200
C	-2.27100	-2.03600	-1.64900
N	-1.25000	-2.20200	-0.67100
C	-1.46800	-2.45500	0.62900
C	-0.30200	-2.15900	1.53900
C	-0.56600	-1.89000	2.99600
C	-1.33500	-0.68100	3.40300
N	-0.49000	0.44800	3.17900
C	-0.63000	1.45200	2.32100
C	0.53700	2.47700	2.21000
N	1.21700	2.11500	0.99900
C	2.33600	1.36300	0.90500
C	2.98900	1.18000	-0.48300
N	2.17400	1.38200	-1.66900
C	1.99200	0.44900	-2.62600
O	-2.52700	-2.80100	1.14100
C	-1.76100	-0.74300	4.88700
O	-0.92700	-0.80000	5.80400
N	-3.03900	-0.76500	5.16100
O	-1.57700	1.51500	1.54500
C	-0.00200	3.95900	2.30600
O	2.85700	0.82700	1.88400
C	4.38400	1.89100	-0.53600
O	2.67400	-0.54900	-2.67500
H	1.12600	-0.44400	-5.50300
H	0.58400	1.68300	-3.71100
H	-0.95900	-0.24000	-4.31200
H	-0.02100	-1.20600	-3.13200
H	-1.48100	1.34300	-2.65200
H	-0.60800	0.33800	-1.38100
H	-3.18400	-0.51800	-2.90700
H	-3.13600	-0.05300	-1.27000
H	-2.03400	-2.62900	-2.53200
H	-3.22500	-2.37700	-1.23300
H	-0.28600	-2.23900	-0.97700
H	0.24600	-1.28400	1.17900
H	0.24400	-3.09900	1.48500
H	0.39900	-1.69700	3.44700
H	-1.13100	-2.68200	3.49200
H	-2.24600	-0.55100	2.83100
H	0.31800	0.55400	3.77200

H	1.25200	2.34200	3.02200
H	0.75700	2.52900	0.19800
H	3.15500	0.10100	-0.43500
H	1.46400	2.09800	-1.70800
H	-3.70100	-0.69900	4.40500
H	-0.70000	4.12600	1.49000
H	-0.65300	4.11900	3.15900
H	4.12800	2.93100	-0.75100
H	4.83300	1.74200	0.44300

Peptide 2 MD analysis (macrocycle)

Peptide 2 cluster1

N	-0.36100	2.01400	-3.12100
C	0.56700	0.89200	-3.22900
C	-0.19100	-0.45700	-3.46600
C	-1.30800	-0.68700	-2.44000
C	-1.98900	-2.05100	-2.69800
C	-3.03600	-2.49500	-1.64300
N	-2.28400	-2.81400	-0.40300
C	-2.59900	-2.26500	0.77300
C	-1.74300	-2.64100	1.93100
C	-0.56200	-1.72900	2.25600
C	-0.86600	-0.37000	2.85500
N	0.28600	0.52200	2.65000
C	0.25300	1.82700	2.95000
C	1.64700	2.42700	2.89700
N	2.31100	2.08900	1.57800
C	2.14200	2.55400	0.39300
C	3.12700	2.13500	-0.72000
N	2.24700	1.94200	-1.84700
C	1.57500	0.83200	-2.16800
O	-3.43400	-1.42100	0.85700
C	-1.31000	-0.53000	4.31900
O	-0.49100	-0.56200	5.27900
N	-2.57500	-0.68800	4.43500
O	-0.81300	2.41100	3.23900
C	1.66300	3.94400	3.10200
O	1.18500	3.30800	0.07900
C	4.17500	3.28600	-1.11800
O	1.96900	-0.25700	-1.63500
H	-0.77100	2.14900	-2.20600
H	1.17000	0.94900	-4.14300
H	-0.49400	-0.42900	-4.50900
H	0.52300	-1.28200	-3.40200
H	-1.93800	0.18000	-2.27600
H	-0.78600	-0.81900	-1.48900
H	-1.28900	-2.87200	-2.81600
H	-2.54100	-2.04200	-3.64000
H	-3.68800	-3.30900	-1.94300
H	-3.71000	-1.67900	-1.39900
H	-1.46900	-3.40500	-0.46200
H	-1.35600	-3.65400	1.82500
H	-2.45900	-2.72300	2.75600
H	-0.02200	-1.58400	1.32300

H	0.10800	-2.24200	2.95700
H	-1.65500	0.22600	2.38100
H	1.12600	0.07800	2.32400
H	2.19600	1.94400	3.70700
H	3.03400	1.39100	1.54700
H	3.66300	1.23700	-0.42700
H	2.11100	2.78200	-2.39400
H	-3.10800	-0.62500	3.57900
H	0.93500	4.23600	3.85000
H	1.22700	4.45000	2.24200
H	4.66100	3.66900	-0.22300
H	3.64900	4.14300	-1.54000

Peptide 2 cluster2

N	-0.36400	1.70100	-4.33700
C	0.80800	0.80800	-4.10500
C	0.27200	-0.67600	-3.91800
C	-0.72700	-0.95100	-2.76600
C	-1.42200	-2.32200	-2.84800
C	-2.36800	-2.62300	-1.68200
N	-1.75500	-2.39600	-0.41800
C	-2.25500	-1.56700	0.56900
C	-1.50900	-1.54200	1.92900
C	-1.92500	-0.43700	2.93400
C	-0.98000	-0.31800	4.09900
N	0.40300	-0.03000	3.65000
C	0.84900	1.15900	3.27100
C	2.28600	1.18200	2.72800
N	2.20500	2.01000	1.49700
C	1.62900	1.62700	0.31800
C	1.61800	2.61600	-0.86500
N	0.99900	2.02000	-1.98200
C	1.56300	1.17400	-2.86800
O	-3.38500	-1.07600	0.43900
C	-1.03700	-1.56100	4.98800
O	-2.03100	-1.64000	5.71500
N	-0.14000	-2.58600	4.84400
O	0.27600	2.24300	3.58800
C	3.18700	1.91100	3.75100
O	1.24400	0.46700	0.16100
C	0.86900	3.92200	-0.57000
O	2.70400	0.78100	-2.72500
H	-0.80500	2.04900	-3.49700
H	1.47400	0.79700	-4.96500
H	-0.25600	-1.01400	-4.80900
H	1.08800	-1.40200	-3.83400
H	-1.42500	-0.11800	-2.74900
H	-0.08600	-0.90800	-1.87800
H	-0.58100	-3.01000	-2.92400
H	-2.02000	-2.46000	-3.73800
H	-2.68000	-3.66300	-1.70200
H	-3.32300	-2.10400	-1.76100
H	-0.88500	-2.88400	-0.28900
H	-0.47700	-1.40700	1.61300
H	-1.62200	-2.58900	2.23600

H	-2.93300	-0.51700	3.34600
H	-1.87700	0.45100	2.30000
H	-1.31500	0.53700	4.69500
H	0.89700	-0.85500	3.34300
H	2.65400	0.19000	2.46700
H	2.50400	2.97200	1.51100
H	2.66900	2.81900	-1.06700
H	0.17800	2.54400	-2.23900
H	0.58600	-2.40600	4.17100
H	3.13800	1.37200	4.69300
H	2.87000	2.95300	3.88700
H	0.96500	4.24800	0.46600
H	-0.21100	3.77700	-0.70500

Peptide 2 cluster3

N	0.88600	0.80100	-4.33700
C	1.11600	-0.23700	-3.34500
C	-0.03900	-1.14600	-3.14600
C	-1.36300	-0.38200	-2.86200
C	-2.62700	-1.24100	-2.95200
C	-2.79700	-2.33000	-1.87200
N	-2.39900	-1.90900	-0.61100
C	-1.92500	-2.66000	0.34300
C	-1.78200	-2.00700	1.67600
C	-0.32100	-1.55600	1.77700
C	0.08600	-0.96800	3.16500
N	0.81500	0.33100	3.02200
C	0.64500	1.49300	3.61600
C	1.34400	2.76300	3.08200
N	1.91500	2.65700	1.76700
C	1.33400	2.31600	0.61000
C	2.25000	2.40400	-0.70900
N	1.77600	1.65600	-1.90500
C	1.62300	0.36200	-2.03400
O	-1.61100	-3.84500	0.14200
C	0.87200	-1.93800	4.01400
O	2.07300	-2.17400	3.73000
N	0.15900	-2.55300	4.95500
O	-0.24900	1.61000	4.41600
C	0.33700	3.93800	3.14400
O	0.18400	1.94800	0.52300
C	2.30400	3.93500	-1.07800
O	1.85700	-0.34000	-1.05500
H	0.12700	1.44700	-4.17700
H	1.91800	-0.85500	-3.74200
H	-0.08200	-1.50300	-4.18000
H	0.14700	-1.86400	-2.35100
H	-1.55100	0.38800	-3.61000
H	-1.36300	0.07100	-1.86600
H	-2.94100	-1.53200	-3.95200
H	-3.46700	-0.55400	-2.81100
H	-2.13100	-3.16300	-2.13800
H	-3.79200	-2.76800	-1.86100
H	-2.88500	-1.06600	-0.35000
H	-1.85200	-2.74300	2.47100

H	-2.58100	-1.28200	1.82500
H	-0.01000	-0.93000	0.93900
H	0.32300	-2.43100	1.61000
H	-0.85800	-0.76700	3.67700
H	1.63800	0.22400	2.44600
H	2.11500	2.99600	3.81100
H	2.90500	2.84700	1.74100
H	3.24100	2.04900	-0.45900
H	1.45800	2.30200	-2.61600
H	-0.85200	-2.58200	4.98100
H	-0.13200	4.07600	4.11600
H	-0.52900	3.83000	2.49900
H	2.87800	3.87200	-2.00300
H	2.86800	4.54100	-0.36700

Peptide 2 cluster4

N	-0.51700	2.16300	-1.94200
C	0.27700	1.33700	-2.89400
C	-0.60600	0.12400	-3.29600
C	-1.33200	-0.61800	-2.10000
C	-1.75500	-1.95200	-2.58500
C	-2.57000	-2.62800	-1.50100
N	-1.81700	-2.78300	-0.27400
C	-2.32900	-2.54800	0.91000
C	-1.36200	-2.53800	2.08400
C	-1.59900	-1.47000	3.17200
C	-1.65300	-0.03800	2.69800
N	-0.32700	0.58300	2.79700
C	0.23200	1.41500	1.90200
C	1.49700	2.08700	2.28200
N	2.25500	2.33500	1.07400
C	3.11100	1.54800	0.52200
C	3.53500	1.77200	-0.95600
N	2.33500	1.98900	-1.72300
C	1.70100	1.03200	-2.42300
O	-3.52800	-2.45800	1.09900
C	-2.56800	0.72700	3.54000
O	-3.66500	1.21800	3.09600
N	-2.18100	1.15400	4.75800
O	-0.41400	1.76500	0.88400
C	1.12300	3.51400	2.73300
O	3.54400	0.54400	1.05100
C	4.45600	2.98300	-1.09700
O	2.13500	-0.13200	-2.43700
H	-0.22100	1.98300	-0.99200
H	0.42000	1.95400	-3.77900
H	-1.40900	0.42300	-3.98400
H	0.09000	-0.45200	-3.89600
H	-2.26000	-0.16500	-1.74800
H	-0.65200	-0.66900	-1.25300
H	-0.99400	-2.68700	-2.83100
H	-2.38800	-1.71900	-3.44500
H	-2.77100	-3.59100	-1.96800
H	-3.54700	-2.20700	-1.28000
H	-0.84000	-2.98100	-0.10700

H	-0.29100	-2.53800	1.86600
H	-1.51700	-3.46900	2.64300
H	-0.87600	-1.60600	3.97100
H	-2.62000	-1.63300	3.52200
H	-2.06000	0.11900	1.70200
H	0.29400	0.56100	3.59500
H	2.06100	1.52800	3.02800
H	1.96700	3.16700	0.59500
H	4.20600	0.96600	-1.25800
H	1.76100	2.77000	-1.44200
H	-1.19400	0.98200	4.91200
H	0.64300	3.40400	3.71300
H	0.51000	4.03100	1.99300
H	3.89400	3.85600	-0.77200
H	4.65500	3.17600	-2.15400

Peptide 2 cluster5

N	-1.18100	1.00500	-1.87300
C	0.01100	0.46300	-2.44700
C	-0.06700	-1.04200	-2.54100
C	-1.28000	-1.39700	-3.43800
C	-1.76000	-2.84300	-3.19400
C	-2.85700	-3.09900	-2.07300
N	-2.44900	-2.80600	-0.69100
C	-2.78800	-1.74900	-0.03300
C	-2.17300	-1.61800	1.40000
C	-0.85500	-0.80400	1.42300
C	-0.24300	-0.57300	2.82600
N	0.76900	0.45900	2.62600
C	1.22100	1.26300	3.58900
C	2.17600	2.36500	3.21000
N	2.61000	2.35500	1.76900
C	2.00900	2.92900	0.75900
C	2.56600	2.74000	-0.65000
N	1.57200	2.10700	-1.50400
C	1.12500	0.90100	-1.39700
O	-3.22400	-0.73600	-0.65400
C	0.40100	-1.90400	3.33600
O	-0.30100	-2.61800	4.05800
N	1.62100	-2.25800	2.91200
O	0.96700	1.02500	4.77000
C	1.80100	3.76600	3.76200
O	1.01300	3.59000	0.89700
C	2.89700	4.12900	-1.24500
O	1.53500	0.14200	-0.55100
H	-1.72200	0.28100	-1.42000
H	0.26200	0.95200	-3.38800
H	0.82000	-1.43400	-3.03900
H	-0.25200	-1.41700	-1.54100
H	-1.01900	-1.31500	-4.49100
H	-2.17800	-0.79600	-3.36100
H	-0.91300	-3.48400	-2.94300
H	-2.12400	-3.13900	-4.18100
H	-3.11800	-4.15100	-2.04700
H	-3.74400	-2.52400	-2.32700

H	-1.80600	-3.50600	-0.35600
H	-2.04200	-2.54400	1.97500
H	-3.01200	-1.25700	1.99900
H	-0.98300	0.13600	0.89500
H	-0.14600	-1.47600	0.93700
H	-0.99300	-0.21000	3.52000
H	1.13200	0.40000	1.67600
H	3.04200	1.93000	3.70100
H	3.37100	1.71500	1.59900
H	3.49300	2.16300	-0.55200
H	1.04100	2.58500	-2.20900
H	2.23200	-1.61700	2.42000
H	1.85100	3.81700	4.85000
H	0.77700	4.03400	3.48300
H	2.02300	4.78700	-1.18300
H	2.96200	3.96600	-2.31700

Peptide 2 cluster6

N	0.30100	1.91600	-5.19400
C	0.91600	0.91000	-4.25600
C	-0.25400	-0.07800	-3.86500
C	0.09900	-1.19400	-2.76600
C	-1.22100	-1.72400	-2.30000
C	-1.05800	-2.55500	-1.03900
N	-1.91500	-2.03100	0.05200
C	-1.67000	-2.09500	1.35200
C	-2.69700	-1.36500	2.21800
C	-2.21000	-1.11900	3.67300
C	-1.33200	0.17400	3.85100
N	-0.11200	-0.05100	3.15500
C	0.93500	0.81600	2.99300
C	1.99300	0.34400	1.98800
N	2.26700	1.31500	0.93400
C	1.36400	1.72100	0.03700
C	1.74000	2.82100	-0.99200
N	1.19900	2.35300	-2.27400
C	1.71800	1.49500	-3.07100
O	-0.52500	-2.40200	1.78000
C	-1.14000	0.49800	5.38500
O	-1.89900	1.34400	5.87600
N	-0.11900	-0.08200	6.00300
O	0.92400	1.92500	3.51400
C	3.33500	0.09700	2.65300
O	0.24200	1.27400	-0.10500
C	1.12300	4.15300	-0.52800
O	2.86600	1.10000	-2.97100
H	-0.69500	1.98000	-5.34600
H	1.66800	0.33300	-4.78300
H	-1.09400	0.45900	-3.44400
H	-0.64900	-0.52400	-4.77400
H	0.75700	-0.97800	-1.92400
H	0.61500	-2.09500	-3.10700
H	-1.75800	-2.28700	-3.06200
H	-1.85400	-0.87200	-2.02500
H	-0.00100	-2.62800	-0.78100

H	-1.37800	-3.58600	-1.18700
H	-2.70800	-1.48800	-0.27700
H	-3.66400	-1.85500	2.14900
H	-2.90300	-0.41400	1.72900
H	-1.67600	-2.00400	4.02500
H	-2.97800	-1.17000	4.45600
H	-1.98000	0.92900	3.40400
H	-0.12300	-0.90800	2.62700
H	1.67200	-0.53000	1.42400
H	3.10200	1.85900	1.11400
H	2.76800	3.00100	-1.27300
H	0.21400	2.51600	-2.42700
H	0.58700	-0.56200	5.47400
H	3.64800	0.87700	3.34400
H	4.09000	0.12300	1.86900
H	0.22100	3.98300	0.05600
H	0.87300	4.87300	-1.30500

Peptide 2 cluster7

N	0.58600	1.80100	-3.69200
C	1.09000	0.42900	-3.30000
C	-0.08600	-0.50300	-3.33400
C	-1.18600	-0.39700	-2.37000
C	-2.19600	-1.56200	-2.64700
C	-3.11700	-1.93400	-1.39400
N	-2.20200	-2.35600	-0.32200
C	-2.04000	-1.68900	0.77500
C	-1.31400	-2.54300	1.80700
C	0.00300	-2.05400	2.28000
C	0.04300	-0.86400	3.32000
N	0.24900	0.40200	2.55800
C	-0.22100	1.65000	2.86100
C	0.20300	2.71500	1.83400
N	1.03200	2.30200	0.78500
C	2.39400	2.13000	0.83200
C	3.11800	1.90600	-0.47400
N	2.19700	1.61300	-1.51900
C	1.81900	0.43000	-1.94300
O	-2.33500	-0.50500	0.96600
C	1.25700	-1.10100	4.27400
O	2.30500	-0.66400	3.92100
N	1.02900	-1.73800	5.39300
O	-1.00700	1.94400	3.80900
C	-1.19700	3.22800	1.35100
O	3.01700	2.14800	1.85200
C	4.02300	3.01600	-0.92400
O	2.15900	-0.63700	-1.45400
H	0.02900	2.28000	-2.99500
H	1.79700	0.16500	-4.09300
H	-0.54200	-0.43300	-4.31700
H	0.26700	-1.50900	-3.13500
H	-1.67000	0.56800	-2.52700
H	-0.74100	-0.48500	-1.38100
H	-1.54200	-2.40700	-2.82000
H	-2.74900	-1.37100	-3.56300

H	-3.71300	-2.82500	-1.56100
H	-3.79400	-1.14800	-1.07800
H	-1.98300	-3.34100	-0.30000
H	-1.17100	-3.57500	1.48700
H	-2.03500	-2.61000	2.61500
H	0.55100	-1.70400	1.40300
H	0.59400	-2.91700	2.60300
H	-0.93100	-0.89400	3.82200
H	0.94700	0.33100	1.83600
H	0.76600	3.48500	2.35600
H	0.68500	2.32300	-0.17000
H	3.73400	1.00000	-0.45600
H	1.91000	2.43900	-2.02600
H	0.11900	-2.17700	5.47000
H	-1.64600	3.67100	2.24600
H	-1.87500	2.40600	1.10100
H	3.46900	3.95000	-0.83900
H	4.27900	2.92900	-1.97400

References

1. Cicero, D. O.; Barbato, G.; Bazzo, R., NMR Analysis of Molecular Flexibility in Solution: A New Method for the Study of Complex Distributions of Rapidly Exchanging Conformations. Application to a 13-Residue Peptide with an 8-Residue Loop. *J. Am. Chem. Soc.* **1995**, *117* (3), 1027-1033.

2. Nevins, N.; Cicero, D.; Snyder, J. P., A Test of the Single-Conformation Hypothesis in the Analysis of NMR Data for Small Polar Molecules: A Force Field Comparison. *J. Org. Chem.* **1999**, *64* (11), 3979-3986.
